# A host shift as the origin of tomato bacterial canker caused by Clavibacter michiganensis

**DOI:** 10.1099/mgen.0.001309

**Published:** 2024-10-29

**Authors:** Alan Guillermo Yañez-Olvera, Ambar Grissel Gómez-Díaz, Nelly Sélem-Mojica, Lorena Rodríguez-Orduña, José Pablo Lara-Ávila, Vanina Varni, Florencia Alcoba, Valentina Croce, Thierry Legros, Alberto Torres, Alfonso Torres Ruíz, Félix Tarrats, Adriaan Vermunt, Thorben Looije, Angélica Cibrian-Jaramillo, Miryam Valenzuela, María Inés Siri, Francisco Barona-Gomez

**Affiliations:** 1 Evolution of Metabolic Diversity Laboratory, Centro de Investigación y Estudios Avanzados del Instituto Politécnico Nacional (Cinvestav-IPN), Irapuato, Guanajuato, Mexico; 2 Institute of Biology, Leiden University, Leiden, The Netherlands; 3 Laboratorio de Microbiología Molecular, Departamento de Biociencias, Facultad de Química, Universidad de la República, Montevideo, Uruguay; 4 Red Sun Farms, Pénjamo, Guanajuato, Mexico; 5 Departamento de Investigación y Desarrollo, Koppert México, Querétaro, Mexico; 6 Centro Universitario CEICKOR, Bernal, Querétaro, Mexico; 7 Normec Groen Agro Control, Delfgauw, The Netherlands; 8 Valto B.V, De Lier, The Netherlands; 9 Naturalis Biodiversity Center, Leiden, The Netherlands; 10 Universidad Técnica Federico Santa María, Valparaíso, Chile

**Keywords:** bacterial canker, *Clavibacter michiganensis*, host shift, pangenomics, phylogenomics, tomato

## Abstract

The Actinomycetota (formerly Actinobacteria) genus *Clavibacter* includes phytopathogens with devasting effects in several crops. *Clavibacter michiganensis*, the causal agent of tomato bacterial canker, is the most notorious species of the genus. Yet, its origin and natural reservoirs remain elusive, and its populations show pathogenicity profiles with unpredictable plant disease outcomes. Here, we generate and analyse a decade-long genomic dataset of *Clavibacter* from wild and commercial tomato cultivars, providing evolutionary insights that directed phenotypic characterization. Our phylogeny situates the last common ancestor of *C. michiganensis* next to *Clavibacter* isolates from grasses rather than to the sole strain we could isolate from wild tomatoes. Pathogenicity profiling of *C. michiganensis* isolates, together with *C. phaseoli* and *C. californiensis* as sister taxa and the wild tomato strain, was found to be congruent with the proposed phylogenetic relationships. We then identified gene enrichment after the evolutionary event, leading to the appearance of the *C. michiganesis* clade, including known pathogenicity factors but also hitherto unnoticed genes with the ability to encode adaptive traits for a pathogenic lifestyle. The holistic perspective provided by our evolutionary analyses hints towards a host shift event as the origin of *C. michiganensis* as a tomato pathogen and the existence of pathogenic genes that remain to be characterized.

**Impact Statement**

*Clavibacter michiganensis* is the causal agent of the devasting plant disease bacterial canker of tomato. Even though this disease was discovered more than a century ago, its origins have remained unknown. Our search for *Clavibacter* isolates in wild and commercial tomato varieties followed by evolutionary genome analysis provided evidence that this tomato pathogen emerged after a host shift from grasses. Genes that make *C. michiganensis* a successful pathogen during the transition between these hosts were identified. Our results help understand how a bacterial endophyte evolves into a pathogenic bacteria and how new pathogens may arise in the agricultural context.

## Data Summary

The following external software was used for this research:

Trimmomatic. DOI: 10.1093/bioinformatics/btu170. Available from: https://academic.oup.com/bioinformatics/article/30/15/2114/2390096

ONT-Guppy. Available from: https://community.nanoporetech.com/docs/prepare/library_prep_protocols/Guppy-protocol/v/gpb_2003_v1_revax_14dec2018/guppy-software-overview

Fitlong. Available from: https://github.com/rrwick/Filtlong

Unicycler. DOI: 10.1371/JOURNAL.PCBI.1005595. Available from: https://github.com/rrwick/Unicycler

CheckM. DOI: 10.1101/gr.186072.114. Available from: https://github.com/Ecogenomics/CheckM

Anvi’o. DOI: 10.1038 /s41564-020-00834-3. Available from: https://anvio.org/

Blast. Available from: https://blast.ncbi.nlm.nih.gov/Blast.cgi

MCL. DOI: 10.1007/978-1-61779-361-5_15. Available from: https://micans.org/mcl/

PyANI. DOI: 10.1039/C5AY02550H. Available from: https://github.com/widdowquinn/pyani

IQ-TREE 2. DOI: 10.1093/MOLBEV/MSAA015. Available from: http://www.iqtree.org/

UFBoot. DOI: 10.1093/molbev/msx281. Available as part of IQ-TREE2.

ModelFinder. DOI: 10.1038/nmeth.4285. Available as part of IQ-TREE2.

Caper. Available from: https://caper.r-forge.r-project.org/

Agricolae. Available from: https://cran.r-project.org/web/packages/agricolae/index.html

The authors confirm all supporting data, code and protocols have been provided within the article or through supplementary data files

## Introduction

Many aspects of human activity are related to the emergence of new pathogens [1]. Climate change, human invasion of ecosystems and urbanization can break ecological barriers and create paths that potential pathogens can use to reach new niches [2]. Agricultural monocultures and high-density cultivation have been linked to the emergence of new phytopathogens [3] and to increased selective pressures that may favour virulent lineages [4]. The introduction of crops to novel environments is another factor that may cause the emergence of pathogens [5], as these can be transmitted from wild to domesticated hosts, even between unrelated species [6]. During this process, host shifts may take place [7], i.e. a pathogen is transmitted among plants from different species, representing the origin of an emerging disease.

The *Clavibacter* genus, which belongs to the phylum Actinomycetota (formerly Actinobacteria), is mainly known due to its phytopathogenic members capable of generating severe diseases in crops of great economic importance [8,9]. The *Clavibacter* phylogeny originally included six subclades referred to as subspecies, which have now been recognized as species [10], with several new species being proposed [11,12]. Among these, *Clavibacter michiganensis* has received a great deal of attention as it is responsible for bacterial canker disease in tomato, *Solanum lycopersicum* [13], with devastating consequences [9,14]. First isolated more than a century ago from tomato crops in Michigan, USA [15], to date, molecular markers with poor reproducibility are the sole means to tackle this infectious agent [16,17]. The latter may reflect the fact that genetically diverse *C. michiganensis* populations exist around the globe [18,21] and that the origins and natural reservoirs of this phytopathogen remain unknown.

It was not until recently that researchers began to describe genetic features that make this bacterium pathogenic. Meletzus *et al.* [22] found evidence that plasmids harboured by *C. michiganensis* contained sequences that encode virulence factors, including *celA* on the pCM1 plasmid, which encodes an endo-β−1,4-glucanase [23], and *pat-1* on pCM2, which encodes a serine protease [24]. Genome sequencing of the reference strain NCPPB 382 further allowed the identification of a pathogenicity island (PAI) present in the chromosome [25]. More recently, omics analyses showed that carbohydrate-active enzymes (CAZymes) families involved in cellulose and hemicellulose degradation [26,27] are abundant in *C. michiganensis*. These previously identified traits have been unambiguously shown to be related to the development of the disease, but there is also evidence that some of these factors are absent from strains capable of causing symptoms of the disease [19,28] and that virulent strains can infect tomato plants without leading to symptoms [29]. These observations suggest that other unknown factors are required to trigger the disease and the development of symptoms [30,31].

Comparative genomics of *C. michiganensis* has shown that while each *Clavibacter* species has acquired unique characteristics [32], pathogenicity factors are shared amongst *C. michiganensis* and other non-pathogenic *Clavibacter* strains [11]. However, previous genomic comparisons have overlooked the relationship between the origin of isolation (host) and the bacterial genomic changes, leading to a pathogenic lifestyle. To tackle these limitations, here we perform a comprehensive phylogenomic and pangenomic analysis of the *Clavibacter* genus, with an emphasis on *C. michiganensis* strains isolated and sequenced throughout a decade in geographically distant and unrelated sites in North and South America (Mexico and Uruguay, respectively) and in Europe (the Netherlands). Our results provide phylogenomic and phenotypic evidence for * C. michiganensis* species sharing a common ancestor with isolates from different grasses and for an evolutionary bottleneck concomitant with the enrichment of functionally relevant genes. Thus, our study paves the way to further functionally characterize the pathogenicity of *C. michiganensis* in line with its dual symptomatic and asymptomatic behaviour and hints towards a recent host shift as the emergence of tomato bacterial canker.

## Methods

### Bacterial strain isolation and taxonomic identification

A total of 511 tomato (*S. lycopersicum*) plants with symptoms, but also asymptomatic, were collected during the period 2010–2020 from several geographically distant sites in Mexico (Fig. S1, available in the online version of this article), mainly from high-tech greenhouses (States of Aguascalientes, Colima, Guanajuato, Michoacán, Nuevo León, Querétaro and Zacatecas; Table S1) and from 39 plants (*S. lycopersicum* var. *cerasiforme*) in two wild populations in the states of Jalisco and Guanajuato (Table S2). Samples consisted of stems, leaves and fruit when available. Tissue was cut into slices of ~0.5 cm^2^ of surface area and placed directly into Petri dishes with CMM1 semi-selective media [33]. CMM1 Petri dishes were incubated at 28 °C, and growth was monitored for 24–72 h. Grown colonies were selected based on *C. michiganensis* reported morphology [34] and isolated and cultured on fresh Petri dishes with CMM1 media. LB (Lisogeny broth medium, also known as Luria-Bertani) liquid cultures were prepared for each isolate for DNA extraction. DNA extraction was carried out with a modified version of the phenol–chloroform extraction protocol described by Mesquita *et al*. [35], without the addition of proteinase K.

The putative *C. michiganensis* isolates were identified by PCR using the *clvF* marker gene [17]. *clvF* PCR-negative isolates were identified by Sanger sequencing of the PCR-amplified 16S rRNA gene. The analysed 511 plant specimens from greenhouses and 39 from wild populations led to a strain collection of *Clavibacter*-positive isolates, consisting of 148 confirmed *C. michiganensis* strains out of 150 *Clavibacter*-positive isolates, solely from Mexico. The two non-*C. michiganensis* isolates corresponded to *C. capsici*, strain RA1B and a *Clavibacter sp.* that remains to be unambiguously taxonomically identified. This strain collection was complemented by 17 *C*. *michiganensis* isolates from the Netherlands, isolated in 2020 according to EPPO (European and Mediterranean Plant Protection Organization) standards [34] by Groen Agro Control, and by three *C. michiganensis* strains from Uruguay, termed MAI 1001, MAI 1050 and MAI 1009, previously described elsewhere [36].

### Genome sequencing and database construction

Genomic DNA from 150 *Clavibacter* strains from Mexico and 17 strains from the Netherlands was sequenced using Illumina paired-end MiSeq (2×150) or NextSeq 550 (2×150) platforms. Read quality was assessed with FastQC. Sequences were trimmed with Trimmomatic [37]. *De novo* assembly was performed with the smart and auto-assembly strategies in PATRIC [38]. Strains from Uruguay were sequenced using Illumina and Oxford Nanopore Technologies platforms. Library and paired-end short-read sequencing was carried out by Macrogen Inc., Seoul, Korea, with Illumina NovaSeq. Poor-quality sequences were trimmed with Trimmomatic [37]. For long-read sequencing, libraries were prepared with the ligation sequencing kit SQK-LSK109 and the native barcoding expansion kit EXP-NBD104 for multiplexing. The libraries were run on a MinION sequencer with an R9.4.1 flow cell. Long reads were base called with ONT-Guppy v6.1.7 and filtered with Filtlong 0.2.1. Hybrid assembly was performed by combining long and short reads using Unicycler [39].

The quality of assemblies was analysed using CheckM [40]. Strict cut-off values for assembly quality were used to select for genomes subsequently used in our genus-level analysis (N50 ≥2400 and completeness ≥90%). This resulted in 80 genomes (79 confirmed *C. michiganensis* and 1 *C*. *capsici*, i.e. strain RA1B). The RAST tool kit was used for gene calling and function prediction [41], which was checked against the Pfam database. Our 79 *C*. *michiganensis* genomes, together with 40 publicly available * C. michiganensis* genomes, were used to construct a species-level database (sDB). To select Mexican representative strains highlighted after phylogenetic reconstructions and pangenome analysis (to be used for posterior analysis), a presence and frequency matrix of gene families was obtained as follows.

Strains within the same clade were compared with each other using an in-house script that scores them according to their gene family content derived from a presence and frequency matrix. The script used an algorithm that awards a higher score to those genomes that had a number of genes for a given gene family, i.e. the gene copy number, which is similar to the mode of the genome’s respective clade in the most shared gene families amongst the same clade (Supplementary Methods S1). After this analysis, six representative genomes out of the 148 *C*. *michiganensis* strains isolated and sequenced in Mexico were chosen for further experimentation. During the course of our investigation, a first selection analysis was performed in 2019 with a smaller sDB to begin with *in planta* assays, and thus, the final sDB presented in the final version of this manuscript might lead to different scores. To avoid overrepresentation of strain from our collection in the subsequent genus-level analysis, only the six selected genomes from Mexico, plus one randomly selected genome from the Netherlands (all genomes from this location were highly similar) and three genomes from Uruguay were selected. These genomes were complemented with 58 publicly available *Clavibacter* genomes to construct a genomic genus database (gDB) of 69 genomes. The gDB included genomes for the *Clavibacter* species *C. californiensis* (1 genome), *C. capsici* (3 genomes), *C. insidiosus* (4 genomes), *C. michiganensis* (32 genomes), *C. nebraskensis* (4 genomes), *C. phaseoli* (3 genomes), *C. sepedonicus* (3 genomes), *C. tessellarius* (2 genomes), *C. zhanzhiyongii* (1 genome) and *Clavibacter* sp. (16 genomes).

### *Clavibacter* phylogeny and pangenome analysis

For both the sDB and gDB, single-copy core genes were identified using Anvi’o v7.0 pangenome analysis tool [42]. Identical genes were filtered out based on the functional homogeneity index (<0.999 for the sDB and <0.98 for the gDB). A total of 100 and 1231 genes were selected for the species-level phylogeny and genus dataset, respectively. Aligned and concatenated amino acid sequences were obtained with *anvi-get-sequences-for-gene-clusters*. The concatenated sequences were used to infer phylogenetic relationships by generating phylogenetic trees with IQ-TREE 2 [43]. Branch support was assessed using the ultrafast bootstrap approximation from UFBoot [44] with 1000 replicates. The best-fit substitution model was determined using ModelFinder [45], restricting the testing procedure to the WAG [46] and LG [47] models. Branches of the *Clavibacter* genus phylogeny were ordered using a family-level reference tree, which included 15 *Clavibacter* strains and *Rathayibacter toxicus* FH232 as a root (Fig. S5). sDB and gDB were analysed using the program *anvi-pangenome* from Anvi’o, using default parameters unless stated otherwise. Briefly, the program calculated the similarity between amino acid gene sequences with BLASTp and then resolved gene families with the MCL algorithm [48] using an inflation value of 10. Eren *et al.* [42] refer them to as ‘gene clusters’, which we call ‘gene families’ to avoid confusion when referring to biosynthetic gene clusters (BGCs). For the gDB, a pairwise average nucleotide identity (ANI) analysis of all the genomes was performed with *anvi-compute-genome-similarity* using PyANI [49] with blastn and default values.

### Identification of genomic changes within the phylogeny

Gene family enrichment analysis was performed at the genus level using Anvi’o’s *anvi-compute-functional-enrichment* program and the *–include-gc-identity-as-function* option. This program groups the genomes in the dataset according to a categorical variable (in our case, membership to the so-called ‘Broad *C. michiganensis* clade’) and then determines which gene families are enriched in the genomes within our group of interest and absent, or nearly absent, in the rest of the genomes. The statistical approach to determine the enrichment was described elsewhere [50]. Parallelly, we looked for phylogenetic signals in the occurrence of the identified gene families with the D stadistic [51] using caper (v. 1.0.1) R package [52]. D was estimated for each gene family using the *Clavibacter* genus phylogeny and the presence and absence of the gene families in each genome as the binary trait to evaluate. Gene families were considered enriched when their enrichment score was ≤50, their q-adjusted value from the enrichment analysis was <1e-10 and the value of D was <0. For the identification of evolutionary and functionally informative loci, genes from the reference strain NCPPB 382 occurring in the enriched gene families were extracted using Anvi’o pangenome analysis tool.

Since NCPPB 382 is a closed genome, the order of the annotated genes in each genome corresponds to their position in the chromosome of this organism. Only genes with at least three enriched neighbouring genes were considered for further inspection. Genes were still considered neighbours even when there was a gap of maximum two non-enriched genes between them. Genes within the PAI were identified according to their location relative to the genes that limit this region, as reported by Gartemann *et al.* [25]. The putative function of the obtained loci was inferred from the gene annotation per RAST and Pfam. ProteInfer [53] was used for annotation of the hypothetical or genes of unknown function included in locus 8. Functional annotation of BGCs was confirmed with antiSMASH [54].

### Identification of homologous loci outside the *Clavibacter* genus

Genes of the *C. michiganensis* strain NCPPB 382 belonging to the gene families identified in the pangenomic analysis were used as a reference for a search of homologous genes outside the *Clavibater* genus. A BLASTp search was performed using the NCPPB 382 genes as queries and the NCBI non-redundant database with an *e*-value of 0.001. The *Clavibacter* genus was excluded from the search. Resultant hits were clustered and grouped per gene family and the associated OTU (Operational Taxonomic Unit). OTUs with hits for complete sets of gene families of pangenome-identified loci were selected for searches of homologous loci. For locus 4, OTUs hits with five or more gene families were selected, whereas, for the PAI, three or more contiguous gene families were selected. Genomes of the selected OTUs were downloaded from the RefSeq and GenBank databases for loci search using CORASON [55] with default values except for the PAI, where a cluster_radio value of 45 was employed. The genome of NCPPB 382 was used as a reference, and genes from each locus were used as queries. Genes used as a query for each case are specified in Table S5.

### Phenotypic characterization *in planta*

Disease development assays were performed at a greenhouse in July 2020, June 2021 and August 2021. *Clavibacter-*free tomato plantlets with three to four true leaves were provided by a commercial supplier (Plantanova, Mexico) and inoculated with selected strains: *C. californiensis* CFBP 8216 and *C. phaseoli* CFBP 8217 (acquired from the corresponding type strain collection), the wild tomato isolate *C. capsici* RA1B and *C. michiganensis* MX15-115 and MX16-I12A. The latter two strains (out of the six representative Mexican strains) were chosen due to their high degree of pathogenicity. Bacterial inoculation was made by scraping the surface (0.5 cm^2^) of the stem with a needle below the first two true leaves and then puncturing slightly at the centre of the scrapped area. The inoculum consisted of 5 µl of bacterial culture with a concentration of 1.5×10^9^ c.f.u. Mock-inoculated control plants were generated using sterile water. Disease development was monitored in a weekly fashion for 6 weeks after inoculation. Disease progression classification and disease index calculation were performed as previously [21] on a weekly basis to obtain disease progress curves. The area under disease progress curve (AUDPC) was calculated using the *audpc* function from the agricolae package in R [56].

## Results

### *C. michiganensis* isolation and genomic database

Given the known relationship between *C. michiganensis* and tomato crop cultivars, we hypothesized that wild tomato plants could act as a reservoir for this species. If so, these wild isolates could provide valuable insights into the evolution of * C. michiganensis*. Hence, we attempted to isolate *Clavibacter* strains from wild tomato populations in Mexico. After sampling 39 plants from two different populations, we isolated 222 bacterial strains on semi-selective CMM1 medium. Based on the 16S rRNA gene of these isolates, almost all of them did correspond to the *Micrococcales* order (Fig. S2). However, only one isolate, termed RA1B and isolated from a wild plant with no noticeable disease symptoms, belonged to the *Clavibacter* genus. ANI analysis performed after genome sequencing showed that RA1B resembles *C. capsici* and not *C. michiganensis*: RA1B has 90.3% identity when compared with *C. michiganensis* NCPPB 382 and 98.9% when compared with *C. capsici* PF008 (Fig. S3).

**Fig. 1.**
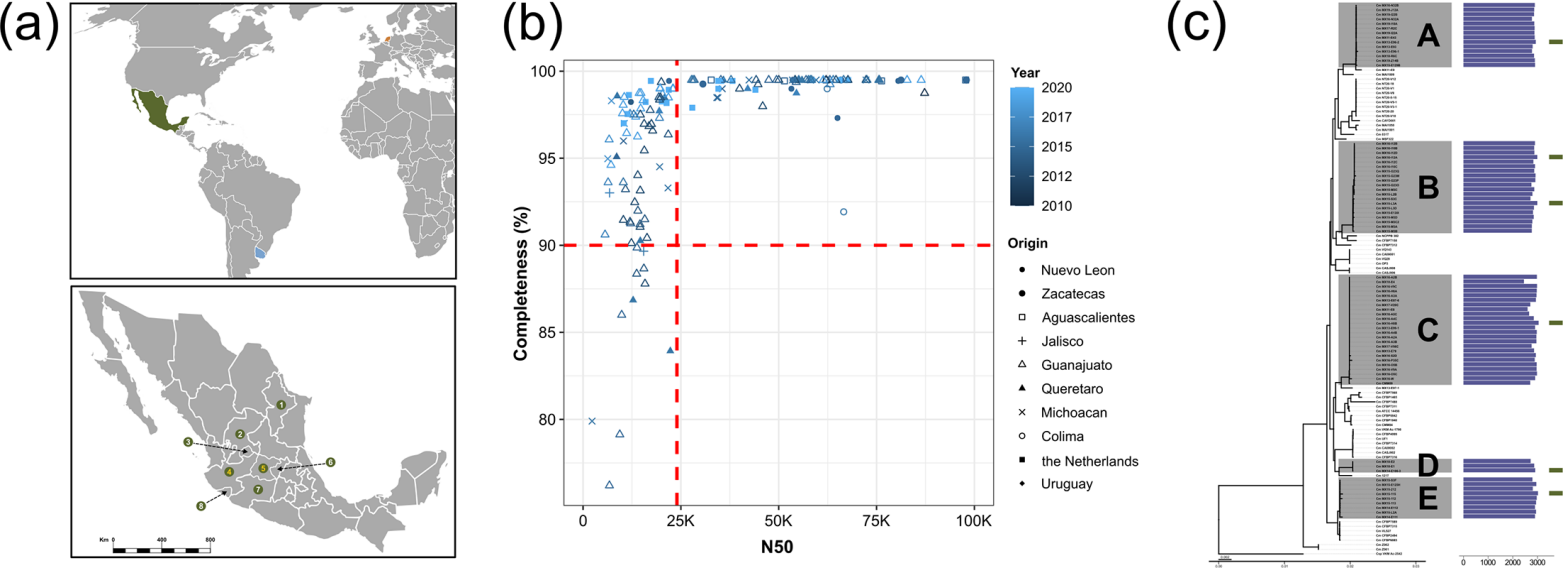
*Clavibacter* isolation and genome selection workflow. (a) Up: *Clavibacter* strains were isolated from tomato plants in three countries: Uruguay, the Netherlands and Mexico. Down: strains from Mexico were isolated from several commercial greenhouses in states throughout the country: 1=Nuevo León, 2=Zacatecas, 3=Aguascalientes, 4=Jalisco, 5=Guanajuato, 6=Querétaro, 7=Michoacán and 8=Colima. Numbers in yellow, 4 and 5, indicate that wild tomato plants were also sampled in such states. (**b)** Genomes from the isolated strains were sequenced and checked for quality. Strict quality cut-off values were established for the genomes to be selected for subsequent analysis (N50 ≥24 000 and completeness ≥90%, indicated with dashed lines in red). (**c)** In the case of the Mexican *C. michiganensis* strains, phylogenetic relationships with other *C. michiganensis* and gene family content were assessed to select representative genomes. The chosen strains were MX13-E96-2 (clade A), MX16-I12A (clade B), MX15-L3A (clade B), MX16-H8B (clade C), MX14-E106-3 (clade D) and MX15-115 (clade E). A detailed tree can be found in Fig. S4.

In addition to sampling wild tomato plants, we surveyed commercial greenhouses from diverse geographical sites in Mexico during a decade-long period, with sampling efforts throughout the year but within the tomato production months where symptoms become noticeable (March–December). After sampling more than 511 plants representing a broad range of commercial cultivars, we isolated 148 *C*. *michiganensis-*positive isolates (based on *clvF* PCR diagnostics) and sequenced their genomes. This collection of genomes was complemented by 17 and 3 genomes of isolates from crops in the Netherlands and Uruguay, respectively (Fig. 1a). Due to the overrepresentation of Mexican *C. michiganensis* genomes in our dataset, we selected representative genomes of our collection. Specifically, to avoid redundancy and enrichment for high-quality genomes, we performed a phylogenomic analysis at the species level, including all genomes, which allowed us to identify subclades (Figs 1b and c and S4, Table S3). Strains that best represented the diversity within each of their respective subclades were identified after profiling for the presence and gene copy number of gene families in their genomes. The previous analysis gave place to the selection of six *C. michiganensis* genomes from the Mexican isolates, together with the three genomes from Uruguay and one from the Netherlands, which were included on a genus-level database, or gDB, complemented with 58 genomes from different *Clavibacter* species, plus the wild tomato *C. capsici* strain RA1B (Table S4).

### *Clavibacter* has undergone multiple host shifts unrelated to wild tomatoes

**Fig. 2.**
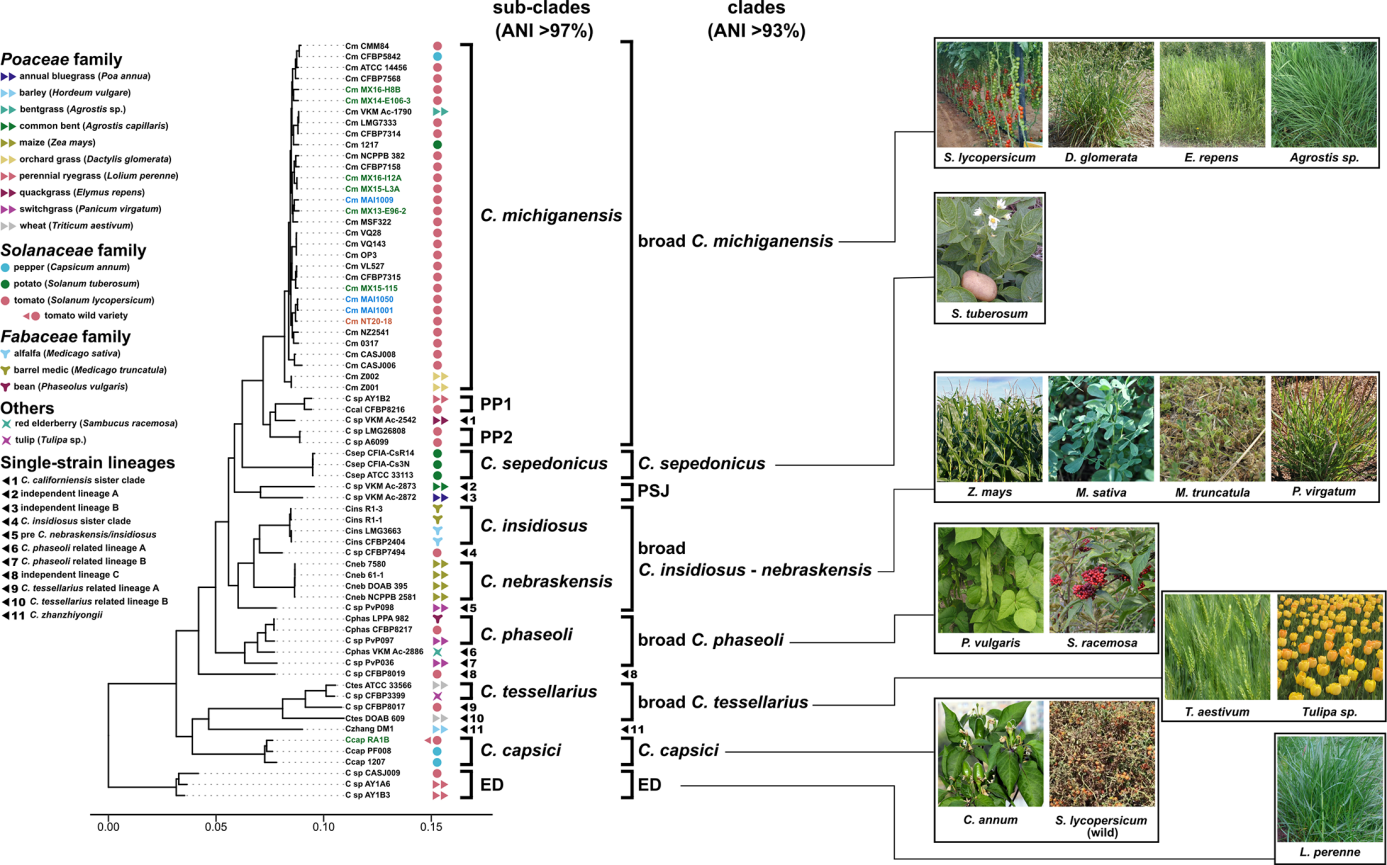
Phylogenomic tree of the *Clavibacter* genus and their hosts of origin. Left. Phylogenomic tree with clades and lineages indicated with brackets and numbers. Each plant host species is indicated by the coloured geometrical shapes next to the bacterial strains’ designation. Non-pathogenic single-strain lineages (SSL) numbers 1, 4, 5, 6, 7 and 10 cluster with pathogenic strains. NMTC strains are distributed throughout the tree. The remaining relevant clades discussed in the text are as follows: ED, early divergent; PP1, pre-phytonotic event-1; PP2, pre-phytonotic event-2; PSJ, pre-*Solanaceae* Jump. *Clavibacter* species included are labelled as follows: *Cm, C. michiganensis; Csp, Clavibacter* sp.; *Ccal, C. californiensis; Csep, C. sepedonicus; Cneb, C. nebraskensis; Cins, C. insidiosus; Cphas*, *C. phaseoli; Czhang, C. zhanzhiyongii; Ccap, C. capsici; Ctes, C. tessellarius*. The tree was inferred from 1231 core proteins. Branch support values are shown in detail in Fig. S5. Right. Representative pictures of plant hosts from which strains were isolated. Complete names of host species are given in the legend (left) and Table S4.

The fact that we managed to isolate a *C. capsici* strain from a wild tomato variety prompted us to analyse the phylogeny of * C. michiganensis* and related species and its host congruence at the genus level. For this purpose, we reconstructed a highly curated and robust phylogenomic tree using 1231 single-copy genes found to be conserved in the 69 genomes of the gDB (Fig. 2). Clades and subclades were defined based on the tree topology and ANI values (Fig. S3). Monophyletic strains with >97% and >93% ANI similarity were considered part of the same subclade or clade, respectively. Thus, *Clavibacter* strains are grouped into 10 different subclades, plus 11 single-strain lineages (SSLs), which remain populated with further isolates and their genome sequences (Figs 2 and S5). The phylogeny revealed that *Clavibacter* strains come from a more diverse and broader host diversity than expected. Moreover, the host–strain relationship seems to be independent of the bacterial evolutionary history as strains obtained from the same plant species or the same plant families are not clustered in the same clades, but rather distributed throughout the tree. The same trend was observed for the strains with known pathogenicity in plants from the same families.

For instance, the *Clavibacter* species that are pathogenic in plants of the same family do not cluster together but are situated in distantly related clades: *C. nebraskensis*, pathogenic in maize (a *Poaceae* plant), and *C. insidiosus*, pathogenic in alfalfa (a *Fabaceae*), are sister clades. In contrast, *C. capsici*, *C. sepedonicus* and *C. michiganensis*, which infect *Solanaceae* plants, are placed at early but also late divergent subclades. Moreover, both *C. michiganensis* and *C. sepedonicus* diverged after a clade of *Poaceae* isolates that we termed the pre-*Solanaceae* jump clade since the diverging lineage precedes both *Solanaceae*-related species. Interestingly, at least six different SSLs with no reported pathogenic activity form monophyletic clades with the pathogenic strains. Along these lines, the case of the so-called ‘broad *C. michiganensis* clade’ (from now onwards BCm clade) drew our attention. The BCm clade includes the *C. michiganensis* pathogenic subclade clustering with three non-pathogenic groups: SSL1 and the so-called pre-phytonotic event 1 and 2 subclades (PP1 and PP2, Fig. 2). The proposed names for these sub-clades highlight the fact that they descend from the *C. michiganensis* lineage. Overall, these phylogenetic relationships made us speculate about the possibility of a lack of, or at least relaxed, host*–Clavibacter* specificity.

Except for the *C. nebraskensis* subclade, all the subclades consist of strains obtained from at least two different host species. While there are cases where the host species are closely related, even belonging to the same plant genus (e.g. *C. insidiosus*), there are several others where strains grouping together come from distantly related hosts, such as in the *C. tessellarius*, * C. phaseoli* and *C. michiganensis* subclades. Notably, several *Clavibacter* strains isolated from *Poaceae* and *Solanaceae* plants (particularly from tomatoes) are distributed throughout several clades. These include a notable group of strains isolated from tomato plants that do not belong to the *C. michiganensis* subclade, which we called not-michiganensis tomato *Clavibacter* (or NMTC), and strains isolated from different plant families but that clustered within the same subclade, including the so-called ‘early divergent clade’, *C. tessellarius, C. phaseoli* and, notably, *C. michiganensis* (Fig. 2). This latter large clade, whose richness reflects the fact that we and others have sampled it thoroughly, includes several strains obtained from plants other than tomato, with many examples of *Poaceae* hosts plants but not our RA1B wild tomato isolate.

### Pathogenicity profiles of selected strains support the phylogeny and the occurrence of host shifts

**Fig. 3.**
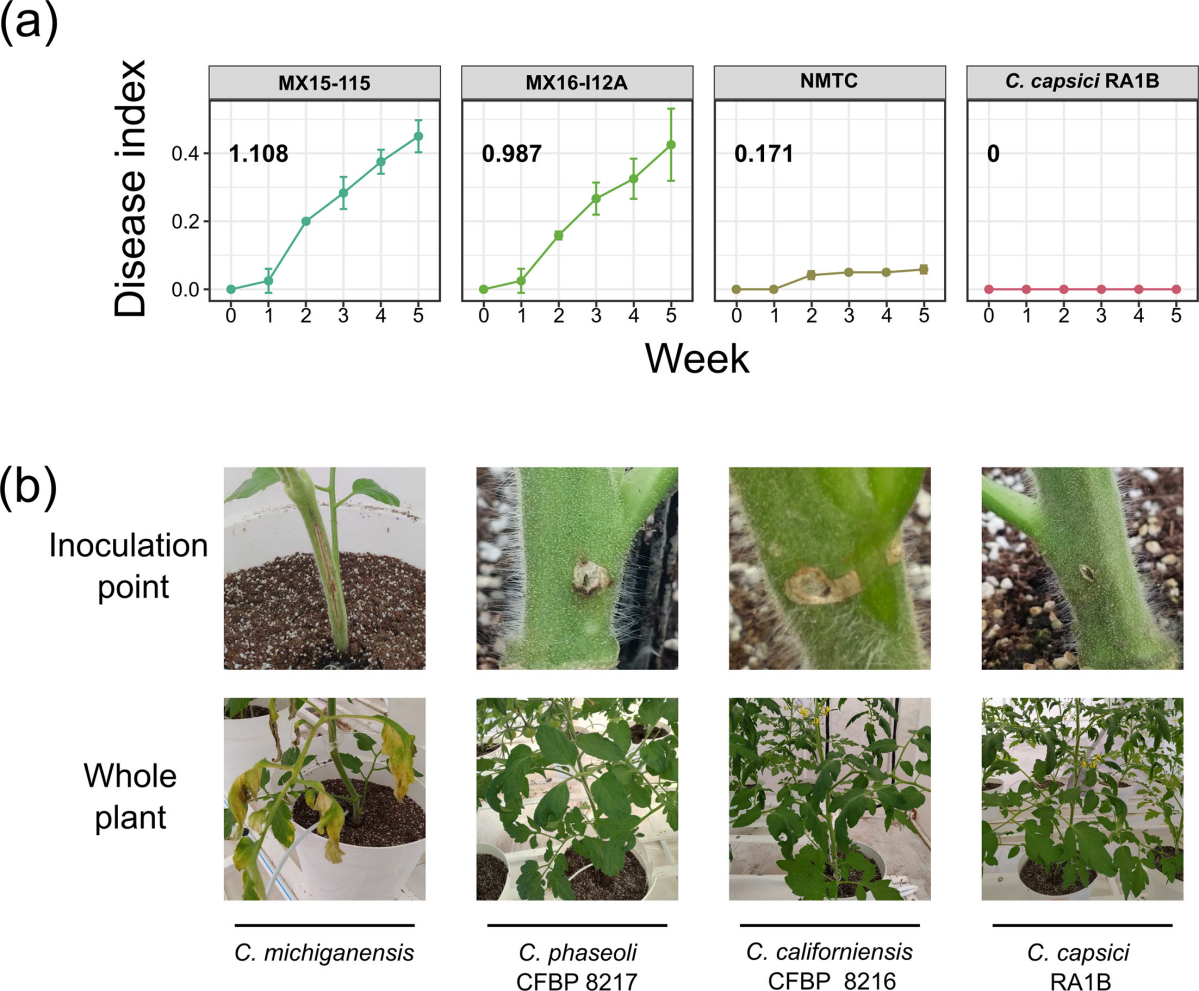
Pathogenicity profiling of selected *Clavibacter* strains. (a) Pathogenicity profiling was quantitatively done for selected strains, by duplicate (two independent experiments), using a disease index calculated for each treatment per week and used to calculate the AUDPC, shown in bold at the top left of each subplot. (**b)** Qualitative comparison of symptoms generated by selected *Clavibacter* strains, *C. michiganensis* (strong symptoms, irrespective of the MX strain used), NMTC strains *C. phaseoli* and *C. californiensis* (mild symptoms only in the site of inoculation) and *C. capsici* RA1B (asymptomatic).

To test the phenotypic implications of our phylogeny, we then assessed the pathogenicity of *C. capsici* RA1B in tomato plants and compared it to the two most pathogenic strains of our *C. michiganensis* collection, strains MX15-115 and MX16-I12A. Moreover, with these experiments, we also aimed at contrasting the pathogenicity profiles of these known pathogens against NMTC strains, namely, *C. californiensis* CFBP 8216 and *C. phaseoli* CFBP 8217, previously reported to be asymptomatic [57]. These two latter strains provided a proxy to the grass strains (not available to us) belonging to the PP1 and *C. phaseoli* subclades (Fig. 2). All *Clavibacter* strains were treated equally and used to infect tomato plants to measure the development of disease symptoms monitored for a period of 6 weeks (Fig. 3). Throughout the period of evaluation, RA1B did not show any sign of being pathogenic in tomato plants. In contrast with previous reports using alternative protocols [57], NMTC-inoculated plants showed the development of mild yet quantifiable and reproducible clear symptoms when compared with the control plants, such as the development of dark tissue around the inoculation site.

### Identification of genetic linkages between grass and tomato crop isolates

When colonizing a new host, pre-adapted pathogens or non-pathogenic endophytes undergo changes that allow them to better thrive in their new environment. To identify these features at the genome level, we compared the gene content of key strains throughout the *Clavibacter* genus and searched for genes that were associated with *C. michiganensis*. To achieve this, we performed a pangenomic analysis at the *Clavibacter* genus level. This analysis identified 11 447 different gene families, of which 1 766 were shared among all the strains in the dataset and 4 183 were unique to one strain (singletons). Since our aim was to identify features common only to *C. michiganensis*, conserved gene families and singletons were discarded, and we continued with analysis with the remaining 5 498 gene families. We then identified gene families acquired at the split of the *C. michiganensis* subclade and its sister clades within the BCm clade, as this would be the time of the proposed host shift taking place. To test this hypothesis in more detail, we employed two complementary approaches, as described next.

**Fig. 4.**
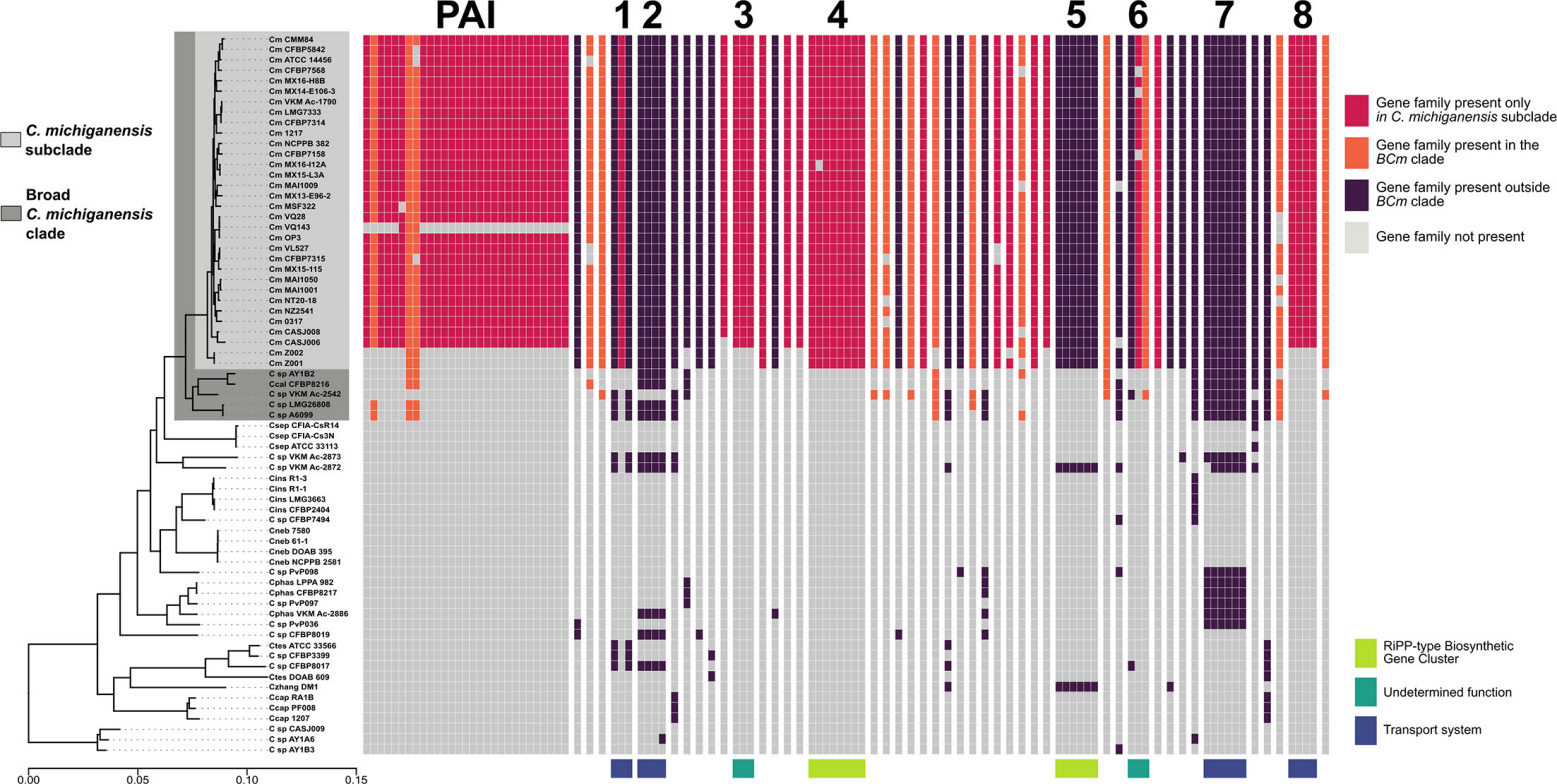
Conserved loci in *C. michiganensis* selected during the proposed host shift. Heat map of characteristic gene families of *C. michiganensis*. Columns represent different gene families in each of the analysed strains throughout the phylogeny. The highlighted clade (dark grey) shows the BCm clade. Purple columns represent gene families found outside the Bcm clade, orange represents gene families found only in the Bcm clade and magenta columns indicate gene families present exclusively in *C. michiganensis* subclade. Columns are ordered according to the occurrence of genes in the genome of *C. michiganensis* NCPPB 382 and clustered when they occur in the same locus. Predicted functions of each locus are indicated with a coloured block below its corresponding columns.

First, a gene enrichment analysis using Anvi’o was adopted, leading to the identification of gene families enriched in the BCm clade and absent outside of it. This allowed us to filter out gene families shared by the strains belonging to the BCm clade from other clades of the entire *Clavibacter* genus. Given that the majority of the BCm clade consists of *C. michiganensis* strains, we expected that the gene families with the highest enrichment scores would be those with a high degree of conservation in this species. Second, we identified gene families whose presence and absence patterns in the genus phylogeny had a phylogenetic signal. This allowed us to discard gene families whose presence, although enriched in the BCm clade, was randomly distributed because of genomic variation. For this, we use the presence and absence of the gene families in the genomes as a binary trait whose phylogenetic signal could be tested under Fritz and Purvis’ D. Conjunctly, these two approaches allowed us to identify 103 gene families that were conserved in the BCm clade (Fig. 4, Table S5). Based on this result, we classified the conserved gene families into three groups with regard to their occurrence outside or inside the BCm clade, plus those exclusively present in the *C. michiganensis* subclade.

Given that operonic gene organization in bacteria is suggestive of functional association, we then searched for evolutionary-relevant genomic regions in *C. michiganensis* by identifying co-locating genes from within the conserved gene families in * C. michiganensis*. Using the genome of strain NCPPB 382 as a reference, we identified nine regions or loci, which were labelled from 1 to 8, plus the PAI as described by Gartemann *et al.* [25] (Fig. 4, left). We found a total of 29 gene families associated with the PAI, of which only three gene families had homologues outside the *C. michiganensis* clade (i.e. a hypothetical protein and the AbiEii toxin/antitoxin complex, also present in the pCM2 plasmid; Table S5). We also found a BGC (loci 5) encoding for the previously reported bacteriocin michiganin, a ribosomally synthesized and post-translationally modified peptide (RiPP) [58], which includes the *clvF* gene used for PCR diagnostics [17]. Despite its use for diagnostic purposes, we found that this BGC occurs outside the *C. michiganensis* clade, albeit at a low frequency. In addition to the PAI and the michiganin BGC, whose identification confirms the validity of the approach adopted, we identified seven additional loci indicative of the evolutionary dynamics leading to *C. michiganensis*, as discussed next.

Three loci included genes exclusively found in *C. michiganensis*, similar to the PAI (i.e. 3, 4 and 8), and four loci included gene families present outside this clade, similar to the michiganin BGC (i.e. 1, 2, 6 and 7). Functional annotation of the corresponding genes predicted their putative roles, which can be associated with the evolution of *C. michiganensis*. These genes could be classified into three different types: (i) transport systems, (ii) RiPP BGC and (iii) undetermined (Fig. 4, right). Four out of the seven newly identified loci (1, 2, 7 and 8) have genes suggestive of transport systems. Genes within loci 2 and 7 were annotated as glycosyl hydrolases, which suggests that the transporters encoded in these loci participate in carbohydrate assimilation. Locus 1 was annotated as a major facilitator-type transporter, but their putative substrates could not be predicted. Functional annotation of locus 8 by ProteInfer hinted towards nitrogen metabolism, including serine metabolism. Also, an unprecedented RiPP BGC, i.e*.* locus 4, could be annotated with antiSMASH. We termed the putative product of this BGC ‘michivionin’, as it shares the same RiPP chemical class III with microvionin from *Microbacterium arborescens* 5913 [59].

### *C.michiganensis* loci are conserved in plant pathogens and endophytes

**Fig. 5.**
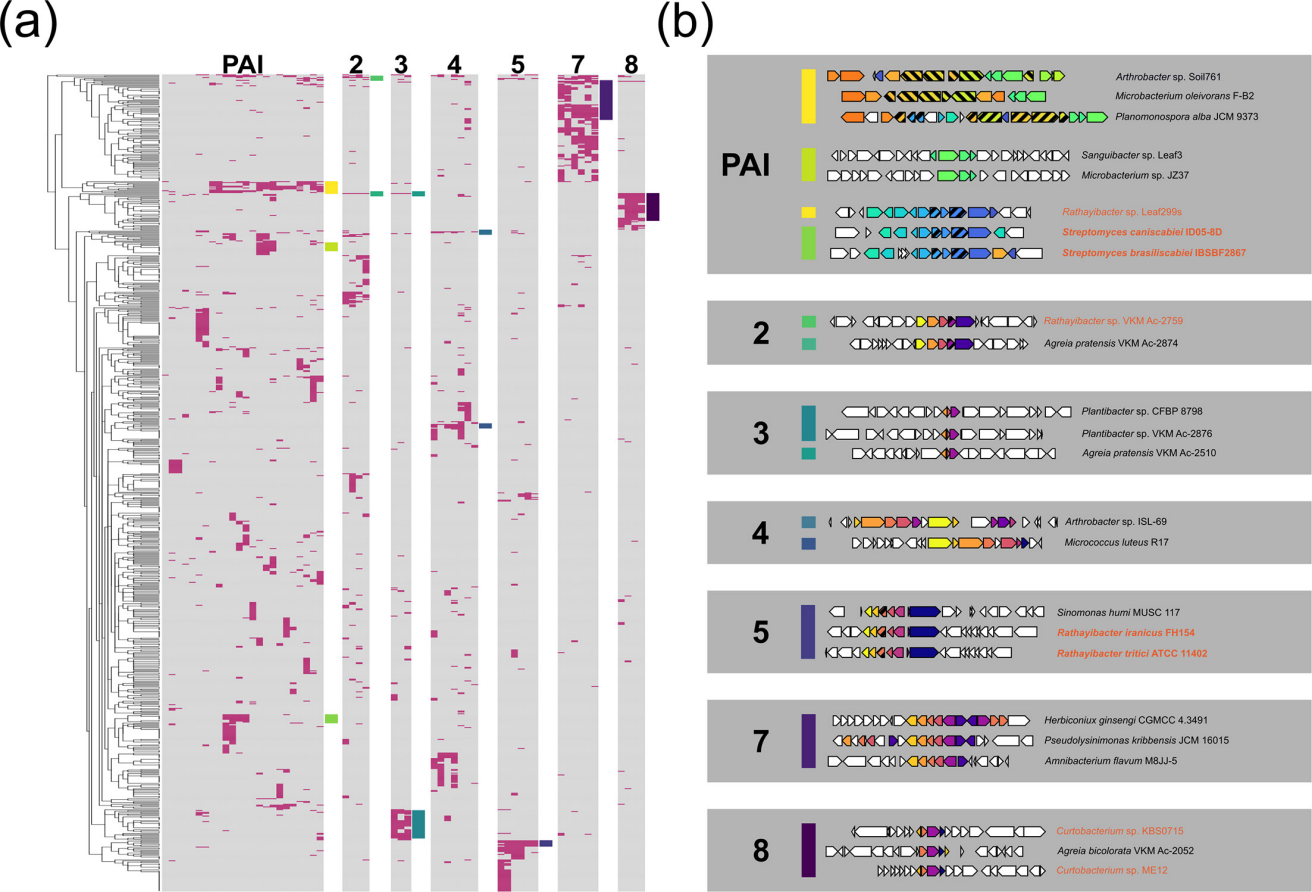
*C. michiganensis* homologous loci found outside the *Clavibacter* genus. (a) *C. michiganensis* conserved gene families in the NCBI non-redundant database. Each row represents a different OTU, while each block of columns is a different set of locus-associated gene families with the corresponding locus indicated on top of the block. The coloured rectangles to the right of each block indicate OTUs or groups of OTUs where homologous loci were found. A detailed version can be found in Fig. S6, which includes non-loci-associated conserved gene families. (b) Representative selection of the *C. michiganensis* homologous loci identified in bacterial genomes outside the *Clavibacter* genus. Locus number or denomination is indicated in bold. The coloured rectangles to the left of the loci correspond to those on the heat map. The stripped pattern over some genes indicates that these and their homologues are not part of the *C. michiganensis* conserved families found in the pangenomic analysis. Names of plant pathogenic strains are shown in bold, while strains from genera with known plant pathogenic members are shown in orange. All homologous loci identified can be found in Figs S7, S8 and S9.

The absence of some gene families outside the *C. michiganensis* subclade suggested the influence of organisms outside this genus in the evolution of this plant pathogen, prompting us to search for homologous genes outside the *Clavibacter* genus. Although not all gene families were exclusive to the *C. michiganensis* subclade, we decided to extend our search to all of its conserved gene families as a means to obtain a general prevalence baseline of these genes in other organisms. We reasoned that this would provide a proxy to determine the relationship between these genes and the lifestyles of micro-organisms similar to *C. michiganensis*. Using the reference strain NCPPB 382, we looked for homologous genes of the *C. michiganensis* conserved gene families in the NCBI non-redundant database (Figs 5a, S6). Except for gene families from locus 6, for which we got no hits, all hits for genes of the conserved gene families were found to be present in actinomycetota OTUs, yet none of them co-occurred in the same OTUs. We then closely examined the gene neighbourhood and genome dynamics of the identified homologues, leading to the confirmation of the presence of homologous loci 2, 3, 4, 5, 7 and 8, and at least partially for the PAI (*tomA* subregion), in several plant actinomycetota genomes (Fig. 5b, S7, S8 and S9). Close comparative inspection of these regions highlighted that many of the species in which these loci occur belong to bacteria from genera with known phytopathogenic members or at least that they were isolated from diverse plant families or plant-derived sources (Table S6). These results provide further evidence of the proposed involvement of the identified conserved loci in the evolution of *C. michiganensis* as a tomato pathogen.

## Discussion

The *Clavibacter* genus has been known for a long time, mainly because of its pathogenic members. With some exceptions, it seems that the available sequenced isolates come from the same hosts where these bacteria can cause disease, in highly relevant crops. However, there are several reports of *Clavibacter* strains being isolated from a variety of sources and asymptomatic plants, including plum [60], coffee [61], poplar [62] and several wild plants from different environments [63,64]. Unfortunately, their genome sequences are not available. Moreover, several of the strains included in our analysis were isolated from sources different from those commonly associated with these pathogenic species. Hence, *Clavibacter* bacteria are not restricted to agricultural systems, and they can thrive in wild plant populations, as evidenced by our analyses and the isolation of RA1B from a wild tomato. Along these lines, our failure to isolate *C. michiganensis* from wild tomatoes using well-validated microbiological methods suggested that wild tomatoes are not a natural reservoir of this pathogen.

Detailed analysis revealed that plants belonging to the *Poaceae* and *Solanaceae* families are the most frequent hosts of *Clavibacter* strains. These plant families are not closely related as shown by previous phylogenetic reconstructions [65], which clearly contrast with the close association between many of the *Clavibacter* strains obtained from such diverse hosts. The occurrence of such relationships throughout the phylogeny, at the subclade and clade levels, and the incongruence between the *Clavibacter* phylogeny and their hosts, indicates the lack of species co-evolution. Host shift is seen as a mechanism used by bacteria for long-term survival, as it allows pathogens to evolve and diversify through radiation and speciation [7,66]. Since host-shifting seems to be a common trend in the *Clavibacter* genus, as shown herein, it is very likely that the known pathogenic species appeared because of such mechanism. Even though *C. michiganensis* is the *Clavibacter* species typically associated with tomato (*S. lycopersicum*) the existence of several tomato isolates not belonging to this species, and their occurrence throughout the genus phylogeny, is indicative of this phenomenon.

As several conditions are required for a successful host shift, our results suggest pre-adaptation as well as the occurrence of a series of events allowing *C. michiganensis* to overcome ecological barriers and colonize tomato plants. The latter could be related to current crop production and commercialization practices, such as the international trade of pathogen-infected seeds [67] and the introduction of crops into novel non-native environments [68] that have the potential to promote the diversification of pathogens [69]. Another factor to be taken into account during the host shift of *C. michiganensis* is the impact of plant domestication and genetic breeding since it is known that domesticated plant varieties assemble and interact differently with microbial communities compared with their wild counterparts [70,71]. This has been shown to hamper the plant’s capacity to assemble a healthy microbiota [72], facilitating the way for pathogens to increase their prevalence in a crop [73]. Any of these factors may have contributed towards the emergence of *C. michiganensis* as a tomato pathogen after a host shift from grasses.

Based on in-depth genomics analyses, we were able to identify genetic signatures of pre-adaptation in the genomic composition of *C. michiganensis*. Our pangenomic analysis highlights that the acquisition of genes represents a key turning point during the evolution of *C. michiganensis* as a tomato pathogen, in turn providing experimentally testable hypotheses. For instance, mutants lacking the PAI, whose genes were identified by our analysis, have been shown to have reduced virulence [25]. Interestingly, *C. michiganensis* strain VQ143, which lacks most of the PAI genes we identified, showed low virulence when tested *in planta* previously by us [21]. Likewise, nutrient-acquiring adaptations are key for bacterial pathogens, and endophytes alike, to thrive in the poor nutrient environment provided by the xylem. In this context, nutrients are not only used for metabolism but also as signals that can trigger environmental-driven specific responses [74]. This is in line with the identification of conserved gene families in *C. michiganensis* encoding for nitrogen and carbohydrate compounds transporter proteins.

The occurrence of RiPP-encoding BGCs as a distinctive feature of *C. michiganensis* and related species suggests that dealing with niche competition with other bacteria was an important strategy to adapt to new hosts [75], such as the tomato plant. It has been reported that the molecules produced by these BGCs have the capacity to affect the growth of closely related bacteria, for instance, michiganin A produced by *C. michiganensis* is capable of inhibiting the growth of *C. sepedonicus in vitro* [58]. However, the compounds produced by RiPP BGCs could have other roles different than antibiosis, as their ability to mediate intra-specific and host–bacteria interactions is well acknowledged [75]. This is a testable hypothesis that warrants further biochemical characterization with potential implications for control and diagnostics of bacterial canker.

The presence of homologous loci conserved in other confirmed plant pathogens and several other plant-isolated *actinomycetota* bacteria indicates that the traits coded in these micro-organisms are mechanisms relevant to a plant–pathogen or plant-associated lifestyle. Moreover, it suggests that some pathogenic or plant colonization capabilities involve the coordinated action of several genes, which contrasts with the many examples of known *C. michiganensis* pathogenicity factors coded in single genes [76]. The most obvious case of the aforementioned are the RiPP BGCs, as discussed above, but also the carbohydrate transport systems, whose association with regulatory genes indicates that these might be Gram-positive polysaccharide utilization loci [77,78], which are tightly regulated mechanisms for complex carbohydrate breakdown. Phylogenetic closeness and niche co-occurrence increase the likelihood of horizontal gene transfer processes to happen [79,80]. While finding homologues of the *C. michiganensis* exclusive gene families in non-*Clavibacter* bacteria is expected, we found it interesting that loci 3, 4, 8 and PAI were not found in other tomato pathogen or tomato isolates. These loci and other *C. michiganensis* exclusive gene families could be more widespread in plant *actinomycetota* bacteria than our analyses suggest. Hence, the potential sources from which *C. michiganensis* could have acquired some of the features highlighted by our research remains to be identified.

Our comprehensive evolutionary genomics analyses, which include new data that doubles the number of *C. michiganensis* genome sequences available, provide insights into the origin of this pathogen. Our findings suggest a host shift as a key event in the evolutionary history of this seed-borne pathogen. Understanding its evolutionary history lightens up several possibilities for its control and diagnosis, which may help prevent the occurrence of similar scenarios involving other *Clavibacter* species, e.g. * C. nebraskensis* [81,82]. Experimental validation of the candidate genes we have identified here will provide a complete picture of the pathogenicity of this endophyte. Our results also point out potential issues during plant breeding and cross-species spillover, which represent hot spots for host shifts [83,84]. In this respect, incorporating strategies such as plant rewilding [85] and microbiome engineering [86,87] might offer more sustainable solutions than treating this phytopathogen as an opportunist with the high costs associated with more traditional methods based on disinfectants and the so-called *Clavibacter*-free certified seeds.

## Repositories

The following genome sequences from *Clavibacter* isolates obtained in Mexico, Uruguay and the Netherlands used for this research will be released on GenBank and NCBI SRA repositories as part of the BioProject PRJNA996097:

**Table:** 

No.	Strain name	Accession number	BioSample
1	Cm_MX10-E2	CP132105	SAMN36730301
2	Cm_MX10-E1	CP132104	SAMN36730302
3	Cm_MX10-E4	CP132103	SAMN36730303
4	Cm_MX11-E8	JAVCVP000000000	SAMN36730304
5	Cm_MX11-E9	JAVCVO000000000	SAMN36730305
6	Cm_MX13-E93	JAVCVN000000000	SAMN36730306
7	Cm_MX15-G23H	JAVCVM000000000	SAMN36730307
8	Csp_MX14-G9D	JAVCVL000000000	SAMN36730308
9	Cm_MX15-E130I	JAVDKJ000000000	SAMN36730309
10	Cm_MX15-L2B	JAVCVK000000000	SAMN36730310
11	Cm_MX15-M3A	JAVDKI000000000	SAMN36730311
12	Cm_MX15-M3B	JAVDKH000000000	SAMN36730312
13	Cm_MX15-M3C	JAVDKG000000000	SAMN36730313
14	Cm_MX15-M3C2	JAVDKF000000000	SAMN36730314
15	Cm_MX15-M3D	JAVDKE000000000	SAMN36730315
16	Cm_MX15-S3C	JAVDKD000000000	SAMN36730316
17	Cm_MX15-S3F	JAVDKC000000000	SAMN36730317
18	Cm_MX15-L2A	JAVCVJ000000000	SAMN36730318
19	Cm_MX15-C3D	JAVCVI000000000	SAMN36730319
20	Cm_MX15-S3E	JAVCVH000000000	SAMN36730320
21	Cm_MX11-E43	JAVDKB000000000	SAMN36730321
22	Cm_MX14-E111	JAVDKA000000000	SAMN36730322
23	Cm_MX14-E112	JAVCVG000000000	SAMN36730323
24	Cm_MX15-E125H	JAVDJZ000000000	SAMN36730324
25	Cm_MX13-E96-1	JAVDJY000000000	SAMN36730325
26	Cm_MX13-E99-1	JAVDJX000000000	SAMN36730326
27	Cm_MX15-G23Q	JAVDJW000000000	SAMN36730327
28	Cm_MX15-113	JAVCVF000000000	SAMN36730328
29	Cm_MX15-212	JAVDJV000000000	SAMN36730329
30	Cm_MX13-E80B	JAVCVE000000000	SAMN36730330
31	Cm_MX13-E79	JAVCVD000000000	SAMN36730331
32	Cm_MX16-I10C	JAVCVC000000000	SAMN36730332
33	Cm_MX16-I10B	JAVCVB000000000	SAMN36730333
34	Cm_MX16-H6C	JAVCVA000000000	SAMN36730334
35	Cm_MX16-H6A	JAVCUZ000000000	SAMN36730335
36	Cm_MX16-V9C	JAVCUY000000000	SAMN36730336
37	Cm_MX16-N32B	JAVCUX000000000	SAMN36730337
38	Cm_MX16-N32A	JAVCUW000000000	SAMN36730338
39	Cm_MX13-E94	JAVCUV000000000	SAMN36730339
40	Cm_MX16-V9A	JAVCUU000000000	SAMN36730340
41	Cm_MX16-I12B	JAVCUT000000000	SAMN36730341
42	Cm_MX16-I12C	JAVCUS000000000	SAMN36730342
43	Cm_MX16-I12D	JAVCUR000000000	SAMN36730343
44	Cm_MX16-A2A	JAVCUQ000000000	SAMN36730344
45	Cm_MX16-A2B	JAVCUP000000000	SAMN36730345
46	Cm_MX16-A3A	JAVCUO000000000	SAMN36730346
47	Cm_MX16-N26B	JAVCUN000000000	SAMN36730347
48	Cm_MX15-G23M	JAVDJU000000000	SAMN36730348
49	Cm_MX15-G23O	JAVCUM000000000	SAMN36730349
50	Cm_MX15-G23P	JAVCUL000000000	SAMN36730350
51	Cm_MX15-L3D	JAVCUK000000000	SAMN36730351
52	Cm_MX16-O5B	JAVCUJ000000000	SAMN36730352
53	Cm_MX16-O5C	JAVDJT000000000	SAMN36730353
54	Cm_MX13-E97-2	JAVCUI000000000	SAMN36730354
55	Cm_MX15-E125E	JAVCUH000000000	SAMN36730355
56	Cm_MX13-E95-2	JAVCUG000000000	SAMN36730356
57	Cm_MX14-E106-9	JAVCUF000000000	SAMN36730357
58	Cm_MX14-E106-12	JAVCUE000000000	SAMN36730358
59	Cm_MX14-E119B	JAVCUD000000000	SAMN36730359
60	Cm_MX14-E119C	JAVCUC000000000	SAMN36730360
61	Cm_MX14-E119D	JAVCUB000000000	SAMN36730361
62	Cm_MX15-E127E	JAVCUA000000000	SAMN36730362
63	Cm_MX16-E137-4	JAVCTZ000000000	SAMN36730363
64	Cm_MX15-117	JAVCTY000000000	SAMN36730364
65	Cm_MX16-216	JAVCTX000000000	SAMN36730365
66	Cm_MX15-E129B	JAVCTW000000000	SAMN36730366
67	Cm_MX16-S2D	JAVCTV000000000	SAMN36730367
68	Cm_MX16-W	JAVDJS000000000	SAMN36730368
69	Cm_MX15-112	JAVDJR000000000	SAMN36730369
70	Cm_MX16-P35C	JAVDJQ000000000	SAMN36730370
71	Cm_MX13-E87-6	JAVCTU000000000	SAMN36730371
72	Cm_MX17-E154D	JAVDJP000000000	SAMN36730372
73	Cm_MX17-E160A	JAVCTT000000000	SAMN36730373
74	Cm_MX18-R6C	JAVCTS000000000	SAMN36730374
75	Cm_MX17-R165D	JAVDJO000000000	SAMN36730375
76	Cm_MX17-R2C	JAVCTR000000000	SAMN36730376
77	Cm_MX18-R6E	JAVDJN000000000	SAMN36730377
78	Cm_MX17-R4D	JAVDJM000000000	SAMN36730378
79	Cm_MX17-R1C	JAVDJL000000000	SAMN36730379
80	Cm_MX16-A2C	JAVCTQ000000000	SAMN36730380
81	Cm_MX16-H6B	JAVCTP000000000	SAMN36730381
82	Cm_MX13-E85-3	JAVCTO000000000	SAMN36730382
83	Cm_MX13-E86-1	JAVCTN000000000	SAMN36730383
84	Cm_MX13-E86-2	JAVCTM000000000	SAMN36730384
85	Cm_MX13-E87-1	JAVCTL000000000	SAMN36730385
86	Cm_MX13-E87-2	JAVCTK000000000	SAMN36730386
87	Cm_MX13-E87-3	JAVCTJ000000000	SAMN36730387
88	Cm_MX13-E87-4	JAVCTI000000000	SAMN36730388
89	Cm_MX13-E87-5	JAVCTH000000000	SAMN36730389
90	Cm_MX13-E91-1	JAVCTG000000000	SAMN36730390
91	Cm_MX13-E91-2	JAVCTF000000000	SAMN36730391
92	Cm_MX13-E91-3	JAVCTE000000000	SAMN36730392
93	Cm_MX13-E95-1	JAVCTD000000000	SAMN36730393
94	Cm_MX13-E99-2	JAVCTC000000000	SAMN36730394
95	Cm_MX13-E100-2	JAVCTB000000000	SAMN36730395
96	Cm_MX14-E106-4	JAVCTA000000000	SAMN36730396
97	Cm_MX14-E106-5	JAVCSZ000000000	SAMN36730397
98	Cm_MX14-E106-6	JAVCSY000000000	SAMN36730398
99	Cm_MX14-E106-7	JAVCSX000000000	SAMN36730399
100	Cm_MX15-E125C	JAVCSW000000000	SAMN36730400
101	Cm_MX16-E137-2	JAVCSV000000000	SAMN36730401
102	Cm_MX16-A3B	JAVCSU000000000	SAMN36730402
103	Cm_MX16-A4B	JAVCST000000000	SAMN36730403
104	Cm_MX16-A4C	JAVCSS000000000	SAMN36730404
105	Cm_MX16-A5C	JAVCSR000000000	SAMN36730405
106	Cm_MX16-V9B	JAVCSQ000000000	SAMN36730406
107	Ccap_RA1B	JAVFKG000000000	SAMN36730407
108	Cm_MX19-P87B	JAVCSP000000000	SAMN36730408
109	Cm_MX19-P88B	JAVCSO000000000	SAMN36730409
110	Cm_MX18-E14A	JAVCSN000000000	SAMN36730410
111	Cm_MX18-E18A	JAVCSM000000000	SAMN36730411
112	Cm_MX16-H6D	JAVCSL000000000	SAMN36730413
113	Cm_MX16-I5R	JAVCSK000000000	SAMN36730414
114	Cm_MX17-V20A	JAVCSJ000000000	SAMN36730415
115	Cm_MX17-V20C	JAVCSI000000000	SAMN36730416
116	Cm_MX17-V56C	JAVCSH000000000	SAMN36730417
117	Cm_MX19-Z2A	JAVCSG000000000	SAMN36730418
118	Cm_MX19-Z5B	JAVCSF000000000	SAMN36730419
119	Cm_MX19-Z5C	JAVDJK000000000	SAMN36730420
120	Cm_MX19-Z6C	JAVCSE000000000	SAMN36730421
121	Cm_MX19-Z11C	JAVCSD000000000	SAMN36730422
122	Cm_MX19-Z14B	JAVCSC000000000	SAMN36730423
123	Cm_MX19-Z17A	JAVCSB000000000	SAMN36730424
124	Cm_MX19-Z18C	JAVCSA000000000	SAMN36730425
125	Cm_MX19-Z18D	JAVCRZ000000000	SAMN36730426
126	Cm_MX19-Z19C	JAVCRY000000000	SAMN36730427
127	Cm_MX18-E3A	JAVCRX000000000	SAMN36730428
128	Cm_MX18-E3B	JAVCRW000000000	SAMN36730429
129	Cm_MX18-E4B	JAVCRV000000000	SAMN36730430
130	Cm_MX19-I16D	JAVCRU000000000	SAMN36730431
131	Cm_MX19-I18A	JAVCRT000000000	SAMN36730432
132	Cm_MX19-I22A	JAVCRS000000000	SAMN36730433
133	Cm_MX19-I22B	JAVCRR000000000	SAMN36730434
134	Cm_MX19-J12A	JAVCRQ000000000	SAMN36730435
135	Cm_MX16-S2P	JAVCRP000000000	SAMN36730436
136	Cm_MX16-A5B	JAVCRO000000000	SAMN36730437
137	Cm_MX19-Z1A	JAVCRN000000000	SAMN36730438
138	Cm_MX19-Z2B	JAVCRM000000000	SAMN36730439
139	Cm_MX19-Z2C	JAVCRL000000000	SAMN36730440
140	Cm_MX19-Z19B	JAVCRK000000000	SAMN36730441
141	Cm_MX19-Z17C	JAVCRJ000000000	SAMN36730442
142	Cm_NT20-5-15	JAVCRI000000000	SAMN36730443
143	Cm_NT20-10	JAVCRH000000000	SAMN36730444
144	Cm_N20-13	JAVCRG000000000	SAMN36730445
145	Cm_N20-16	JAVCRF000000000	SAMN36730446
146	Cm_N20-17	JAVDJJ000000000	SAMN36730447
147	Cm_NT20-18	JAVCRE000000000	SAMN36730448
148	Cm_NT20-20	JAVDJI000000000	SAMN36730449
149	Cm_NT20-2	JAVCRD000000000	SAMN36730450
150	Cm_NT20-8	JAVCRC000000000	SAMN36730451
151	Cm_NT20-11	JAVCRB000000000	SAMN36730452
152	Cm_NT20-V1	JAVCRA000000000	SAMN36730453
153	Cm_NT20-V3-1	JAVCQZ000000000	SAMN36730454
154	Cm_NT20-V5-1	JAVCQY000000000	SAMN36730455
155	Cm_NT20-V6-1	JAVCQX000000000	SAMN36730456
156	Cm_NT20-V8	JAVCQW000000000	SAMN36730457
157	Cm_NT20-V10	JAVCQV000000000	SAMN36730458
158	Cm_NT20-V12	JAVCQU000000000	SAMN36730459
159	Cm_MX14-E106-3	JAVDJH000000000	SAMN36730460
160	Cm_MX13-E96-2	JAVCQT000000000	SAMN36730461
161	Cm_MX16-H8B	JAVCQS000000000	SAMN36730462
162	Cm_MX16-I12A	JAVCQR000000000	SAMN36730463
163	Cm_MX15-L3A	JAVCQQ000000000	SAMN36730464
164	Cm_MX15-115	JAVDJG000000000	SAMN36730465
165	Cm_MX13-E97-1	JAVDJF000000000	SAMN36730466
166	Cm_MAI1001	JAVCQP000000000	SAMN36730467
167	Cm_MAI1009	JAVCQO000000000	SAMN36730468
168	Cm_MAI1050	JAVCQN000000000	SAMN36730469
169	Cm_MX19-Z16A	JAVCQM000000000	SAMN36730470
170	Cm_MX19-I16C	JAVCQL000000000	SAMN36730471

## supplementary material

10.1099/mgen.0.001309 Uncited Supplementary Material 1.

## Abbreviations

ANI: average nucleotide identity
AUDPC: area under the disease progress curve
BCm: broad *C. michiganensis* clade
BGCs: biosynthetic gene clusters
CAZymes: carbohydrate-active enzymes
NMTC: not-michiganensis tomato *Clavibacter*
PAI: pathogenicity island
RiPP: ribosomally synthesized and post-translationally modified peptide
SSL: single-strain lineages
