# Supplementary material for: A host shift as the origin of tomato bacterial canker caused by Clavibacter michiganensis
Source: Microb Genom. 2024 Oct 29;10(10):001309. doi: 10.1099/mgen.0.001309 (PMC11521342; doi:10.1099/mgen.0.001309)
Supplement: Uncited Supplementary Material 1. [file mgen-10-01309-s001.pdf]

Supplementary material

Supplementary Tables

Supplementary table S1. High-tech greenhouse samples metadata.

Supplementary table S2. Wild tomato varieties samples and sites metadata.

Supplementary table S3. *Clavibacter michinganensis* species genome database information.

Supplementary table S4. *Clavibacter* genus genome database information.

Supplementary table S5. *Clavibacter michiganensis* genes from gene families identified by the phylogenetic and pangenomic analyses.

Supplementary table S6. Isolation data of organisms with confirmed presence of *C. michiganensis* loci homolog.

Supplementary methods

Supplementary methods S1. Details of the scoring algorithm used for Mexican *Clavibacter michiganensis* strains selection.

Supplementary tables

Supplementary table S1. High-tech greenhouse samples metadata.

Sample No.	Sampling location	Latitude[a]	Longitude[a]	Sampled plant health status[b]	Sampling date
1	Guanajuato	20.4179286	-102.0495558	Symptomatic	november 2010
2	Guanajuato	20.4179286	-102.0495558	Symptomatic	november 2010
3	Guanajuato	20.4179286	-102.0495558	Symptomatic	december 2010
4	Guanajuato	ND	ND	Symptomatic	december 2010
5	Guanajuato	20.4179286	-102.0495558	Symptomatic	january 2011
6	Guanajuato	20.4179286	-102.0495558	Symptomatic	february 2011
7	Guanajuato	20.4179286	-102.0495558	Symptomatic	may 2011
8	Guanajuato	20.4179286	-102.0495558	Symptomatic	august 2013
9	Guanajuato	20.4179286	-102.0495558	Symptomatic	august 2013
10	Guanajuato	20.4179286	-102.0495558	Symptomatic	november 2013
11	Guanajuato	20.4179286	-102.0495558	Symptomatic	november 2013
12	Guanajuato	20.4179286	-102.0495558	Symptomatic	november 2013
13	Guanajuato	20.4179286	-102.0495558	Symptomatic	november 2013
14	Guanajuato	20.4179286	-102.0495558	Symptomatic	november 2013
15	Guanajuato	20.4179286	-102.0495558	Symptomatic	november 2013
16	Guanajuato	20.4179286	-102.0495558	Symptomatic	november 2013

Sample No.	Sampling location	Latitude[a]	Longitude[a]	Sampled plant health status[b]	Sampling date
17	Guanajuato	20.4179286	-102.0495558	Symptomatic	november 2013
18	Guanajuato	20.4179286	-102.0495558	Symptomatic	november 2013
19	Guanajuato	20.4179286	-102.0495558	Symptomatic	november 2013
20	Guanajuato	20.4179286	-102.0495558	Symptomatic	november 2013
21	Guanajuato	20.4179286	-102.0495558	Symptomatic	november 2013
22	Guanajuato	20.4179286	-102.0495558	Symptomatic	december 2013
23	Guanajuato	20.4179286	-102.0495558	Symptomatic	march 2014
24	Nuevo Leon	24.9149464	-100.4152776	Symptomatic	september 2014
25	Nuevo Leon	24.9149464	-100.4152776	Symptomatic	september 2014
26	Nuevo Leon	24.9149464	-100.4152776	Symptomatic	september 2014
27	Nuevo Leon	24.9149464	-100.4152776	Symptomatic	september 2014
28	Nuevo Leon	24.9149464	-100.4152776	Symptomatic	september 2014
29	Nuevo Leon	24.9149464	-100.4152776	Symptomatic	september 2014
30	Nuevo Leon	24.9149464	-100.4152776	Symptomatic	september 2014
31	Nuevo Leon	24.9149464	-100.4152776	Symptomatic	september 2014
32	Nuevo Leon	24.9149464	-100.4152776	Symptomatic	september 2014
33	Nuevo Leon	24.9149464	-100.4152776	Symptomatic	september 2014
34	Nuevo Leon	24.9149464	-100.4152776	Symptomatic	september 2014
35	Nuevo Leon	24.9149464	-100.4152776	Symptomatic	september 2014

Sample No.	Sampling location	Latitude[a]	Longitude[a]	Sampled plant health status[b]	Sampling date
36	Guanajuato	20.4082232	-101.6769241	Asymptomatic	september 2014
37	Guanajuato	20.4082232	-101.6769241	Asymptomatic	september 2014
38	Guanajuato	20.4082232	-101.6769241	Asymptomatic	september 2014
39	Guanajuato	20.4082232	-101.6769241	Asymptomatic	september 2014
40	Guanajuato	20.4082232	-101.6769241	Asymptomatic	september 2014
41	Guanajuato	20.4082232	-101.6769241	Asymptomatic	september 2014
42	Guanajuato	20.4082232	-101.6769241	Asymptomatic	september 2014
43	Guanajuato	20.4082232	-101.6769241	Asymptomatic	september 2014
44	Guanajuato	20.4179286	-102.0495558	Symptomatic	september 2014
45	Guanajuato	20.4179286	-102.0495558	Symptomatic	september 2014
46	Guanajuato	20.4179286	-102.0495558	Symptomatic	september 2014
47	Guanajuato	20.4179286	-102.0495558	Symptomatic	september 2014
48	Guanajuato	20.4179286	-102.0495558	Symptomatic	september 2014
49	Guanajuato	20.4179286	-102.0495558	Symptomatic	september 2014
50	Guanajuato	20.4179286	-102.0495558	Symptomatic	october 2014
51	Nuevo Leon	24.9149464	-100.4152776	Symptomatic	april 2015
52	Nuevo Leon	24.9149464	-100.4152776	Symptomatic	april 2015
53	Nuevo Leon	24.9149464	-100.4152776	Symptomatic	april 2015
54	Guanajuato	20.4179286	-102.0495558	Symptomatic	july 2015

Sample No.	Sampling location	Latitude[a]	Longitude[a]	Sampled plant health status[b]	Sampling date
55	Guanajuato	20.4179286	-102.0495558	Symptomatic	july 2015
56	Guanajuato	20.4179286	-102.0495558	Symptomatic	july 2015
57	Guanajuato	20.4179286	-102.0495558	Symptomatic	july 2015
58	Guanajuato	20.4179286	-102.0495558	Symptomatic	september 2015
59	Guanajuato	20.4082232	-101.6769241	Asymptomatic	september 2015
60	Guanajuato	20.4082232	-101.6769241	Asymptomatic	september 2015
61	Guanajuato	20.4082232	-101.6769241	Asymptomatic	september 2015
62	Guanajuato	20.4082232	-101.6769241	Asymptomatic	september 2015
63	Guanajuato	20.4082232	-101.6769241	Asymptomatic	september 2015
64	Guanajuato	20.4082232	-101.6769241	Asymptomatic	september 2015
65	Guanajuato	20.4082232	-101.6769241	Asymptomatic	september 2015
66	Guanajuato	20.4082232	-101.6769241	Asymptomatic	september 2015
67	Guanajuato	20.4082232	-101.6769241	Asymptomatic	september 2015
68	Guanajuato	20.4082232	-101.6769241	Asymptomatic	september 2015
69	Guanajuato	20.4082232	-101.6769241	Asymptomatic	september 2015
70	Guanajuato	20.4082232	-101.6769241	Asymptomatic	september 2015
71	Guanajuato	20.4082232	-101.6769241	Asymptomatic	september 2015
72	Guanajuato	20.4082232	-101.6769241	Asymptomatic	september 2015
73	Guanajuato	20.4082232	-101.6769241	Symptomatic	september 2015

Sample No.	Sampling location	Latitude[a]	Longitude[a]	Sampled plant health status[b]	Sampling date
74	Guanajuato	20.4082232	-101.6769241	Symptomatic	september 2015
75	Guanajuato	20.4082232	-101.6769241	Symptomatic	september 2015
76	Guanajuato	20.4082232	-101.6769241	Symptomatic	september 2015
77	Guanajuato	20.4082232	-101.6769241	Asymptomatic	september 2015
78	Guanajuato	20.4082232	-101.6769241	Asymptomatic	september 2015
79	Guanajuato	20.4082232	-101.6769241	Asymptomatic	september 2015
80	Guanajuato	20.4082232	-101.6769241	Asymptomatic	september 2015
81	Guanajuato	20.4082232	-101.6769241	Asymptomatic	september 2015
82	Guanajuato	20.4082232	-101.6769241	Asymptomatic	september 2015
83	Guanajuato	20.4082232	-101.6769241	Asymptomatic	september 2015
84	Guanajuato	20.4082232	-101.6769241	Asymptomatic	september 2015
85	Guanajuato	20.4082232	-101.6769241	Asymptomatic	september 2015
86	Guanajuato	20.4082232	-101.6769241	Asymptomatic	september 2015
87	Guanajuato	20.4082232	-101.6769241	Asymptomatic	september 2015
88	Guanajuato	20.4082232	-101.6769241	Asymptomatic	september 2015
89	Guanajuato	20.4082232	-101.6769241	Asymptomatic	september 2015
90	Guanajuato	20.4082232	-101.6769241	Asymptomatic	september 2015
91	Guanajuato	20.4082232	-101.6769241	Asymptomatic	september 2015
92	Guanajuato	20.4082232	-101.6769241	Asymptomatic	september 2015

Sample No.	Sampling location	Latitude[a]	Longitude[a]	Sampled plant health status[b]	Sampling date
93	Guanajuato	20.4082232	-101.6769241	Asymptomatic	september 2015
94	Guanajuato	20.4082232	-101.6769241	Asymptomatic	september 2015
95	Guanajuato	20.4082232	-101.6769241	Asymptomatic	september 2015
96	Guanajuato	20.4082232	-101.6769241	Asymptomatic	september 2015
97	Guanajuato	20.4082232	-101.6769241	Asymptomatic	september 2015
98	Guanajuato	20.4082232	-101.6769241	Asymptomatic	september 2015
99	Guanajuato	20.4082232	-101.6769241	Asymptomatic	september 2015
100	Guanajuato	20.4082232	-101.6769241	Asymptomatic	september 2015
101	Guanajuato	20.4082232	-101.6769241	Asymptomatic	september 2015
102	Guanajuato	20.4082232	-101.6769241	Asymptomatic	september 2015
103	Guanajuato	20.4082232	-101.6769241	Asymptomatic	september 2015
104	Guanajuato	20.4082232	-101.6769241	Asymptomatic	september 2015
105	Guanajuato	20.4082232	-101.6769241	Asymptomatic	september 2015
106	Guanajuato	20.4082232	-101.6769241	Asymptomatic	september 2015
107	Guanajuato	20.4082232	-101.6769241	Asymptomatic	september 2015
108	Guanajuato	20.4082232	-101.6769241	Asymptomatic	september 2015
109	Guanajuato	20.4082232	-101.6769241	Asymptomatic	september 2015
110	Guanajuato	20.4082232	-101.6769241	Asymptomatic	september 2015
111	Guanajuato	20.4082232	-101.6769241	Asymptomatic	september 2015

Sample No.	Sampling location	Latitude[a]	Longitude[a]	Sampled plant health status[b]	Sampling date
112	Guanajuato	20.4082232	-101.6769241	Asymptomatic	september 2015
113	Guanajuato	20.4082232	-101.6769241	Asymptomatic	september 2015
114	Guanajuato	20.4082232	-101.6769241	Asymptomatic	september 2015
115	Guanajuato	20.4082232	-101.6769241	Symptomatic	september 2015
116	Guanajuato	20.4082232	-101.6769241	Symptomatic	september 2015
117	Guanajuato	20.4082232	-101.6769241	Symptomatic	september 2015
118	Guanajuato	20.4082232	-101.6769241	Symptomatic	september 2015
119	Guanajuato	20.4082232	-101.6769241	Symptomatic	september 2015
120	Guanajuato	20.4082232	-101.6769241	Symptomatic	september 2015
121	Guanajuato	20.4082232	-101.6769241	Symptomatic	september 2015
122	Guanajuato	20.4082232	-101.6769241	Symptomatic	september 2015
123	Guanajuato	20.4082232	-101.6769241	Symptomatic	september 2015
124	Guanajuato	20.4082232	-101.6769241	Symptomatic	september 2015
125	Guanajuato	20.4082232	-101.6769241	Symptomatic	september 2015
126	Guanajuato	20.4082232	-101.6769241	Symptomatic	september 2015
127	Guanajuato	20.4082232	-101.6769241	Symptomatic	september 2015
128	Guanajuato	20.4082232	-101.6769241	Symptomatic	september 2015
129	Guanajuato	20.4082232	-101.6769241	Symptomatic	september 2015
130	Guanajuato	20.4082232	-101.6769241	Symptomatic	september 2015

Sample No.	Sampling location	Latitude[a]	Longitude[a]	Sampled plant health status[b]	Sampling date
131	Guanajuato	20.4082232	-101.6769241	Symptomatic	september 2015
132	Guanajuato	20.4082232	-101.6769241	Symptomatic	september 2015
133	Guanajuato	20.4179286	-102.0495558	Symptomatic	october 2015
134	Guanajuato	20.4082232	-101.6769241	Symptomatic	october 2015
135	Guanajuato	20.4082232	-101.6769241	Symptomatic	october 2015
136	Guanajuato	20.4082232	-101.6769241	Symptomatic	october 2015
137	Guanajuato	20.4082232	-101.6769241	Symptomatic	october 2015
138	Guanajuato	20.4082232	-101.6769241	Symptomatic	october 2015
139	Guanajuato	20.4082232	-101.6769241	Symptomatic	october 2015
140	Guanajuato	20.4082232	-101.6769241	Symptomatic	october 2015
141	Guanajuato	20.4082232	-101.6769241	Symptomatic	october 2015
142	Guanajuato	20.4082232	-101.6769241	Symptomatic	october 2015
143	Guanajuato	20.4082232	-101.6769241	Symptomatic	october 2015
144	Guanajuato	20.4082232	-101.6769241	Symptomatic	october 2015
145	Guanajuato	20.4082232	-101.6769241	Symptomatic	october 2015
146	Guanajuato	20.4082232	-101.6769241	Symptomatic	october 2015
147	Guanajuato	20.4082232	-101.6769241	Symptomatic	october 2015
148	Guanajuato	20.4082232	-101.6769241	Symptomatic	october 2015
149	Guanajuato	20.4082232	-101.6769241	Symptomatic	october 2015

Sample No.	Sampling location	Latitude[a]	Longitude[a]	Sampled plant health status[b]	Sampling date
150	Guanajuato	20.4082232	-101.6769241	Symptomatic	october 2015
151	Guanajuato	20.4082232	-101.6769241	Symptomatic	october 2015
152	Guanajuato	20.4082232	-101.6769241	Symptomatic	october 2015
153	Guanajuato	20.4082232	-101.6769241	Symptomatic	october 2015
154	Guanajuato	20.4082232	-101.6769241	Symptomatic	october 2015
155	Guanajuato	20.4082232	-101.6769241	Symptomatic	october 2015
156	Guanajuato	20.4082232	-101.6769241	Symptomatic	october 2015
157	Guanajuato	20.4082232	-101.6769241	Symptomatic	october 2015
158	Guanajuato	20.4082232	-101.6769241	Symptomatic	october 2015
159	Guanajuato	20.4082232	-101.6769241	Symptomatic	october 2015
160	Guanajuato	20.4082232	-101.6769241	Symptomatic	october 2015
161	Guanajuato	20.4082232	-101.6769241	Symptomatic	october 2015
162	Guanajuato	20.4082232	-101.6769241	Symptomatic	october 2015
163	Guanajuato	20.4082232	-101.6769241	Symptomatic	october 2015
164	Guanajuato	20.4082232	-101.6769241	Symptomatic	october 2015
165	Guanajuato	20.4179286	-102.0495558	Symptomatic	november 2015
166	Queretaro	20.7060935	-99.943661	Asymptomatic	november 2015
167	Queretaro	20.7060935	-99.943661	Asymptomatic	november 2015
168	Queretaro	20.7060935	-99.943661	Asymptomatic	november 2015

Sample No.	Sampling location	Latitude[a]	Longitude[a]	Sampled plant health status[b]	Sampling date
169	Queretaro	20.7060935	-99.943661	Asymptomatic	november 2015
170	Queretaro	20.7060935	-99.943661	Asymptomatic	november 2015
171	Queretaro	20.7060935	-99.943661	Symptomatic	november 2015
172	Queretaro	20.7060935	-99.943661	Symptomatic	november 2015
173	Queretaro	20.7060935	-99.943661	Symptomatic	november 2015
174	Queretaro	20.7060935	-99.943661	Symptomatic	november 2015
175	Queretaro	20.7060935	-99.943661	Symptomatic	november 2015
176	Queretaro	20.7060935	-99.943661	Symptomatic	november 2015
177	Queretaro	20.7060935	-99.943661	Symptomatic	november 2015
178	Queretaro	20.7060935	-99.943661	Symptomatic	november 2015
179	Queretaro	20.7060935	-99.943661	Asymptomatic	november 2015
180	Queretaro	20.7060935	-99.943661	Asymptomatic	november 2015
181	Queretaro	20.7060935	-99.943661	Asymptomatic	november 2015
182	Queretaro	20.7060935	-99.943661	Symptomatic	november 2015
183	Queretaro	20.7060935	-99.943661	Symptomatic	november 2015
184	Queretaro	20.7060935	-99.943661	Symptomatic	november 2015
185	Queretaro	20.7060935	-99.943661	Symptomatic	november 2015
186	Queretaro	20.7060935	-99.943661	Symptomatic	november 2015
187	Aguascalientes	21.9962532	-102.265797	Symptomatic	december 2015

Sample No.	Sampling location	Latitude[a]	Longitude[a]	Sampled plant health status[b]	Sampling date
188	Aguascalientes	21.9962532	-102.265797	Symptomatic	december 2015
189	Aguascalientes	21.9962532	-102.265797	Symptomatic	december 2015
190	Aguascalientes	21.9962532	-102.265797	Asymptomatic	december 2015
191	Jalisco	20.3863663	-102.1548925	Symptomatic	december 2015
192	Jalisco	20.3863663	-102.1548925	Asymptomatic	december 2015
193	Jalisco	20.3863663	-102.1548925	Symptomatic	december 2015
194	Jalisco	20.3863663	-102.1548925	Asymptomatic	december 2015
195	Jalisco	20.3863663	-102.1548925	Symptomatic	december 2015
196	Queretaro	20.7060935	-99.943661	Asymptomatic	december 2015
197	Queretaro	20.7060935	-99.943661	Symptomatic	december 2015
198	Queretaro	20.7060935	-99.943661	Symptomatic	december 2015
199	Queretaro	20.7060935	-99.943661	Symptomatic	december 2015
200	Zacatecas	22.7377245	-102.5561985	Symptomatic	december 2015
201	Zacatecas	22.7377245	-102.5561985	Symptomatic	december 2015
202	Zacatecas	22.7377245	-102.5561985	Asymptomatic	december 2015
203	Guanajuato	20.4179286	-102.0495558	Symptomatic	december 2015
204	Guanajuato	20.4179286	-102.0495558	Symptomatic	december 2015
205	Guanajuato	20.4179286	-102.0495558	Symptomatic	december 2015
206	Guanajuato	20.4179286	-102.0495558	Symptomatic	december 2015

Sample No.	Sampling location	Latitude[a]	Longitude[a]	Sampled plant health status[b]	Sampling date
207	Jalisco	20.3863663	-102.1548925	Asymptomatic	december 2015
208	Jalisco	20.3863663	-102.1548925	Asymptomatic	december 2015
209	Jalisco	20.3863663	-102.1548925	Asymptomatic	december 2015
210	Jalisco	20.3863663	-102.1548925	Asymptomatic	december 2015
211	Jalisco	20.3863663	-102.1548925	Asymptomatic	december 2015
212	Jalisco	20.3863663	-102.1548925	Asymptomatic	december 2015
213	Jalisco	20.3863663	-102.1548925	Asymptomatic	december 2015
214	Jalisco	20.3863663	-102.1548925	Asymptomatic	december 2015
215	Jalisco	20.3863663	-102.1548925	Asymptomatic	december 2015
216	Jalisco	20.3863663	-102.1548925	Asymptomatic	december 2015
217	Jalisco	20.3863663	-102.1548925	Asymptomatic	december 2015
218	Jalisco	20.3863663	-102.1548925	Asymptomatic	december 2015
219	Jalisco	20.3863663	-102.1548925	Asymptomatic	december 2015
220	Jalisco	20.3863663	-102.1548925	Asymptomatic	december 2015
221	Jalisco	20.3863663	-102.1548925	Asymptomatic	december 2015
222	Jalisco	20.3863663	-102.1548925	Asymptomatic	december 2015
223	Jalisco	20.3863663	-102.1548925	Asymptomatic	december 2015
224	Guanajuato	20.719025	-101.329102	Symptomatic	january 2016
225	Guanajuato	20.719025	-101.329102	Symptomatic	january 2016

Sample No.	Sampling location	Latitude[a]	Longitude[a]	Sampled plant health status[b]	Sampling date
226	Guanajuato	20.719025	-101.329102	Symptomatic	january 2016
227	Guanajuato	20.719025	-101.329102	Symptomatic	january 2016
228	Guanajuato	20.719025	-101.329102	Symptomatic	january 2016
229	Guanajuato	20.719025	-101.329102	Symptomatic	january 2016
230	Guanajuato	20.719025	-101.329102	Symptomatic	january 2016
231	Guanajuato	20.719025	-101.329102	Symptomatic	january 2016
232	Guanajuato	20.719025	-101.329102	Symptomatic	january 2016
233	Queretaro	ND	ND	Symptomatic	april 2016
234	Guanajuato	20.4179286	-102.0495558	Symptomatic	june 2016
235	Guanajuato	20.4179286	-102.0495558	Symptomatic	july 2016
236	Jalisco	20.5364923	-103.925692	Asymptomatic	october 2016
237	Jalisco	20.5364923	-103.925692	Asymptomatic	october 2016
238	Jalisco	20.5364923	-103.925692	Asymptomatic	october 2016
239	Jalisco	20.5364923	-103.925692	Asymptomatic	october 2016
240	Jalisco	20.5364923	-103.925692	Asymptomatic	october 2016
241	Jalisco	20.5364923	-103.925692	Asymptomatic	october 2016
242	Jalisco	20.5364923	-103.925692	Asymptomatic	october 2016
243	Jalisco	20.5364923	-103.925692	Asymptomatic	october 2016
244	Jalisco	20.5364923	-103.925692	Asymptomatic	october 2016

Sample No.	Sampling location	Latitude[a]	Longitude[a]	Sampled plant health status[b]	Sampling date
245	Jalisco	20.5364923	-103.925692	Asymptomatic	october 2016
246	Jalisco	20.5364923	-103.925692	Asymptomatic	october 2016
247	Jalisco	20.5364923	-103.925692	Asymptomatic	october 2016
248	Jalisco	20.5364923	-103.925692	Asymptomatic	october 2016
249	Jalisco	20.5364923	-103.925692	Asymptomatic	october 2016
250	Jalisco	20.5364923	-103.925692	Asymptomatic	october 2016
251	Jalisco	20.5364923	-103.925692	Asymptomatic	october 2016
252	Jalisco	20.5364923	-103.925692	Asymptomatic	october 2016
253	Jalisco	20.5364923	-103.925692	Symptomatic	october 2016
254	Jalisco	20.5364923	-103.925692	Symptomatic	october 2016
255	Jalisco	20.5364923	-103.925692	Symptomatic	october 2016
256	Jalisco	20.5364923	-103.925692	Symptomatic	october 2016
257	Jalisco	20.5364923	-103.925692	Symptomatic	october 2016
258	Guanajuato	20.4179286	-102.0495558	Symptomatic	october 2016
259	Guanajuato	20.4179286	-102.0495558	Symptomatic	october 2016
260	Guanajuato	20.4179286	-102.0495558	Symptomatic	november 2016
261	Guanajuato	20.4179286	-102.0495558	Symptomatic	november 2016
262	Guanajuato	20.3726666	-101.2084539	Asymptomatic	november 2016
263	Guanajuato	20.3726666	-101.2084539	Asymptomatic	november 2016

Sample No.	Sampling location	Latitude[a]	Longitude[a]	Sampled plant health status[b]	Sampling date
264	Guanajuato	20.3726666	-101.2084539	Asymptomatic	november 2016
265	Guanajuato	20.3726666	-101.2084539	Symptomatic	november 2016
266	Guanajuato	20.3726666	-101.2084539	Symptomatic	november 2016
267	Guanajuato	20.3726666	-101.2084539	Symptomatic	november 2016
268	Guanajuato	20.3726666	-101.2084539	Asymptomatic	november 2016
269	Guanajuato	20.3726666	-101.2084539	Symptomatic	november 2016
270	Guanajuato	20.3726666	-101.2084539	Symptomatic	november 2016
271	Guanajuato	20.3726666	-101.2084539	Symptomatic	november 2016
272	Guanajuato	20.3726666	-101.2084539	Symptomatic	november 2016
273	Guanajuato	20.3726666	-101.2084539	Symptomatic	november 2016
274	Queretaro	20.4762259	-100.1460428	Symptomatic	december 2016
275	Queretaro	20.4762259	-100.1460428	Symptomatic	december 2016
276	Queretaro	20.4762259	-100.1460428	Symptomatic	december 2016
277	Guanajuato	20.6020938	-100.8564073	Asymptomatic	december 2016
278	Guanajuato	20.6020938	-100.8564073	Symptomatic	december 2016
279	Guanajuato	20.6020938	-100.8564073	Symptomatic	december 2016
280	Guanajuato	20.6020938	-100.8564073	Asymptomatic	december 2016
281	Guanajuato	20.6020938	-100.8564073	Asymptomatic	december 2016
282	Michoacan	20.4090519	-101.8884114	Symptomatic	december 2016

Sample No.	Sampling location	Latitude[a]	Longitude[a]	Sampled plant health status[b]	Sampling date
283	Michoacan	20.4090519	-101.8884114	Symptomatic	december 2016
284	Michoacan	20.4090519	-101.8884114	Symptomatic	december 2016
285	Michoacan	20.4090519	-101.8884114	Symptomatic	december 2016
286	Michoacan	20.4090519	-101.8884114	Symptomatic	december 2016
287	Queretaro	20.6897089	-100.006017	Symptomatic	december 2016
288	Queretaro	20.6897089	-100.006017	Symptomatic	december 2016
289	Queretaro	20.6897089	-100.006017	Symptomatic	december 2016
290	Queretaro	20.6897089	-100.006017	Symptomatic	december 2016
291	Queretaro	20.6897089	-100.006017	Symptomatic	december 2016
292	Queretaro	20.6897089	-100.006017	Symptomatic	december 2016
293	Queretaro	20.6820006	-100.0124288	Symptomatic	december 2016
294	Queretaro	20.6820006	-100.0124288	Symptomatic	december 2016
295	Queretaro	20.6820006	-100.0124288	Symptomatic	december 2016
296	Queretaro	20.6820006	-100.0124288	Symptomatic	december 2016
297	Queretaro	20.6820006	-100.0124288	Symptomatic	december 2016
298	Queretaro	20.6820006	-100.0124288	Symptomatic	december 2016
299	Colima	19.3377402	-103.8058543	Symptomatic	december 2016
300	Colima	19.3377402	-103.8058543	Symptomatic	december 2016
301	Colima	19.3377402	-103.8058543	Symptomatic	december 2016

Sample No.	Sampling location	Latitude[a]	Longitude[a]	Sampled plant health status[b]	Sampling date
302	Colima	19.3377402	-103.8058543	Symptomatic	december 2016
303	Queretaro	20.6820006	-100.0124288	Asymptomatic	january 2017
304	Queretaro	20.6820006	-100.0124288	Symptomatic	january 2017
305	Queretaro	20.6820006	-100.0124288	Symptomatic	january 2017
306	Queretaro	20.6820006	-100.0124288	Symptomatic	january 2017
307	Queretaro	20.6820006	-100.0124288	Symptomatic	january 2017
308	Queretaro	20.6820006	-100.0124288	Symptomatic	january 2017
309	Queretaro	20.6820006	-100.0124288	Symptomatic	january 2017
310	Queretaro	20.6820006	-100.0124288	Symptomatic	january 2017
311	Queretaro	20.6820006	-100.0124288	Symptomatic	january 2017
312	Queretaro	20.6820006	-100.0124288	Symptomatic	january 2017
313	Queretaro	20.6820006	-100.0124288	Symptomatic	january 2017
314	Queretaro	20.6820006	-100.0124288	Symptomatic	january 2017
315	Queretaro	20.6820006	-100.0124288	Symptomatic	january 2017
316	Queretaro	20.6820006	-100.0124288	Symptomatic	january 2017
317	Queretaro	20.6820006	-100.0124288	Symptomatic	january 2017
318	Queretaro	20.6820006	-100.0124288	Symptomatic	january 2017
319	Queretaro	20.6820006	-100.0124288	Symptomatic	january 2017
320	Queretaro	20.6820006	-100.0124288	Symptomatic	january 2017

Sample No.	Sampling location	Latitude[a]	Longitude[a]	Sampled plant health status[b]	Sampling date
321	Queretaro	20.6820006	-100.0124288	Symptomatic	january 2017
322	Queretaro	20.6820006	-100.0124288	Symptomatic	january 2017
323	Queretaro	20.6820006	-100.0124288	Symptomatic	january 2017
324	Queretaro	20.6820006	-100.0124288	Symptomatic	january 2017
325	Guanajuato	20.4179286	-102.0495558	Symptomatic	january 2017
326	Guanajuato	20.4179286	-102.0495558	Symptomatic	january 2017
327	Guanajuato	20.4179286	-102.0495558	Symptomatic	january 2017
328	Colima	19.3377402	-103.8058543	Asymptomatic	may 2017
329	Colima	19.3377402	-103.8058543	Symptomatic	may 2017
330	Guanajuato	20.4179286	-102.0495558	Symptomatic	september 2017
331	Guanajuato	20.4179286	-102.0495558	Symptomatic	september 2017
332	Guanajuato	20.4179286	-102.0495558	Symptomatic	september 2017
333	Guanajuato	20.4179286	-102.0495558	Symptomatic	september 2017
334	Guanajuato	20.4179286	-102.0495558	Symptomatic	september 2017
335	Guanajuato	20.4179286	-102.0495558	Symptomatic	september 2017
336	Guanajuato	21.0532978	-100.5592285	Symptomatic	september 2017
337	Guanajuato	21.0532978	-100.5592285	Symptomatic	september 2017
338	Jalisco	20.3823711	-102.9600625	Symptomatic	september 2017
339	Jalisco	20.3823711	-102.9600625	Symptomatic	september 2017

Sample No.	Sampling location	Latitude[a]	Longitude[a]	Sampled plant health status[b]	Sampling date
340	Guanajuato	20.4179286	-102.0495558	Symptomatic	september 2017
341	Guanajuato	21.0532978	-100.5592285	Symptomatic	october 2017
342	Guanajuato	21.0532978	-100.5592285	Symptomatic	october 2017
343	Guanajuato	20.4179286	-102.0495558	Symptomatic	october 2017
344	Guanajuato	ND	ND	Symptomatic	november 2017
345	Guanajuato	ND	ND	Symptomatic	november 2017
346	Guanajuato	20.4082232	-101.6769241	Asymptomatic	january 2018
347	Guanajuato	20.4082232	-101.6769241	Asymptomatic	january 2018
348	Guanajuato	20.4082232	-101.6769241	Asymptomatic	january 2018
349	Guanajuato	20.4082232	-101.6769241	Asymptomatic	january 2018
350	Guanajuato	20.4082232	-101.6769241	Asymptomatic	january 2018
351	Guanajuato	20.4082232	-101.6769241	Asymptomatic	january 2018
352	Guanajuato	20.4082232	-101.6769241	Asymptomatic	january 2018
353	Guanajuato	20.4082232	-101.6769241	Asymptomatic	january 2018
354	Guanajuato	20.4082232	-101.6769241	Asymptomatic	january 2018
355	Guanajuato	20.4082232	-101.6769241	Asymptomatic	january 2018
356	Guanajuato	20.4082232	-101.6769241	Asymptomatic	january 2018
357	Guanajuato	20.4082232	-101.6769241	Asymptomatic	january 2018
358	Guanajuato	20.4082232	-101.6769241	Asymptomatic	january 2018

Sample No.	Sampling location	Latitude[a]	Longitude[a]	Sampled plant health status[b]	Sampling date
359	Guanajuato	20.4082232	-101.6769241	Asymptomatic	january 2018
360	Guanajuato	20.4082232	-101.6769241	Asymptomatic	january 2018
361	Guanajuato	20.4082232	-101.6769241	Asymptomatic	january 2018
362	Guanajuato	20.4082232	-101.6769241	Asymptomatic	january 2018
363	Guanajuato	20.4082232	-101.6769241	Asymptomatic	january 2018
364	Guanajuato	21.0532978	-100.5592285	Symptomatic	january 2018
365	Guanajuato	21.0532978	-100.5592285	Symptomatic	february 2018
366	Guanajuato	20.9435202	-100.6750294	Symptomatic	march 2018
367	Guanajuato	20.9435202	-100.6750294	Symptomatic	march 2018
368	Guanajuato	20.9435202	-100.6750294	Symptomatic	march 2018
369	Guanajuato	20.4179286	-102.0495558	Symptomatic	april 2018
370	Guanajuato	20.4179286	-102.0495558	Symptomatic	april 2018
371	Guanajuato	20.4179286	-102.0495558	Symptomatic	april 2018
372	Guanajuato	20.4179286	-102.0495558	Symptomatic	april 2018
373	Michoacan	20.2783102	-102.3290048	Symptomatic	june 2018
374	Michoacan	20.2783102	-102.3290048	Symptomatic	june 2018
375	Michoacan	20.2783102	-102.3290048	Symptomatic	june 2018
376	Michoacan	20.2783102	-102.3290048	Symptomatic	june 2018
377	Michoacan	20.2783102	-102.3290048	Symptomatic	june 2018

Sample No.	Sampling location	Latitude[a]	Longitude[a]	Sampled plant health status[b]	Sampling date
378	Michoacan	20.2783102	-102.3290048	Asymptomatic	june 2018
379	Michoacan	20.2783102	-102.3290048	Asymptomatic	june 2018
380	Michoacan	20.2783102	-102.3290048	Asymptomatic	june 2018
381	Michoacan	20.2783102	-102.3290048	Asymptomatic	june 2018
382	Michoacan	20.2783102	-102.3290048	Symptomatic	june 2018
383	Michoacan	20.2783102	-102.3290048	Asymptomatic	june 2018
384	Michoacan	20.2783102	-102.3290048	Symptomatic	june 2018
385	Michoacan	20.2783102	-102.3290048	Asymptomatic	june 2018
386	Michoacan	20.2783102	-102.3290048	Symptomatic	june 2018
387	Michoacan	20.2783102	-102.3290048	Asymptomatic	june 2018
388	Michoacan	20.2783102	-102.3290048	Symptomatic	june 2018
389	Michoacan	20.2783102	-102.3290048	Symptomatic	june 2018
390	Michoacan	20.2783102	-102.3290048	Asymptomatic	june 2018
391	Michoacan	20.2783102	-102.3290048	Asymptomatic	june 2018
392	Michoacan	20.2783102	-102.3290048	Asymptomatic	june 2018
393	Michoacan	20.2783102	-102.3290048	Symptomatic	june 2018
394	Guanajuato	20.4179286	-102.0495558	Symptomatic	july 2018
395	Guanajuato	20.4179286	-102.0495558	Symptomatic	july 2018
396	Guanajuato	20.4179286	-102.0495558	Symptomatic	july 2018

Sample No.	Sampling location	Latitude[a]	Longitude[a]	Sampled plant health status[b]	Sampling date
397	Guanajuato	20.4082232	-101.6769241	Asymptomatic	august 2018
398	Guanajuato	20.4082232	-101.6769241	Asymptomatic	august 2018
399	Guanajuato	20.4082232	-101.6769241	Asymptomatic	august 2018
400	Guanajuato	20.4082232	-101.6769241	Asymptomatic	august 2018
401	Guanajuato	20.4082232	-101.6769241	Asymptomatic	august 2018
402	Guanajuato	20.4082232	-101.6769241	Asymptomatic	august 2018
403	Guanajuato	20.4082232	-101.6769241	Asymptomatic	august 2018
404	Guanajuato	20.4082232	-101.6769241	Asymptomatic	august 2018
405	Guanajuato	20.4082232	-101.6769241	Asymptomatic	august 2018
406	Guanajuato	20.4082232	-101.6769241	Asymptomatic	august 2018
407	Guanajuato	20.4082232	-101.6769241	Asymptomatic	august 2018
408	Guanajuato	20.4082232	-101.6769241	Asymptomatic	august 2018
409	Guanajuato	20.4082232	-101.6769241	Asymptomatic	august 2018
410	Guanajuato	20.4082232	-101.6769241	Asymptomatic	august 2018
411	Guanajuato	20.4082232	-101.6769241	Asymptomatic	august 2018
412	Guanajuato	20.4082232	-101.6769241	Asymptomatic	august 2018
413	Guanajuato	20.4082232	-101.6769241	Asymptomatic	august 2018
414	Guanajuato	20.4179286	-102.0495558	Symptomatic	september 2018
415	Guanajuato	21.0532978	-100.5592285	Symptomatic	october 2018

Sample No.	Sampling location	Latitude[a]	Longitude[a]	Sampled plant health status[b]	Sampling date
416	Guanajuato	21.0532978	-100.5592285	Symptomatic	october 2018
417	Guanajuato	20.4179286	-102.0495558	Symptomatic	october 2018
418	Colima	19.3377402	-103.8058543	Symptomatic	december 2018
419	Colima	19.3377402	-103.8058543	Symptomatic	december 2018
420	Colima	19.3377402	-103.8058543	Asymptomatic	december 2018
421	Colima	19.3377402	-103.8058543	Asymptomatic	december 2018
422	Colima	19.3377402	-103.8058543	Symptomatic	december 2018
423	Colima	19.3377402	-103.8058543	Symptomatic	december 2018
424	Colima	19.3377402	-103.8058543	Symptomatic	december 2018
425	Colima	19.3377402	-103.8058543	Symptomatic	december 2018
426	Guanajuato	21.0532978	-100.5592285	Symptomatic	december 2018
427	Guanajuato	20.4082232	-101.6769241	Asymptomatic	march 2019
428	Guanajuato	20.4082232	-101.6769241	Asymptomatic	march 2019
429	Guanajuato	20.4082232	-101.6769241	Asymptomatic	march 2019
430	Guanajuato	20.4082232	-101.6769241	Asymptomatic	march 2019
431	Guanajuato	20.4082232	-101.6769241	Asymptomatic	march 2019
432	Guanajuato	20.4082232	-101.6769241	Asymptomatic	march 2019
433	Guanajuato	20.4082232	-101.6769241	Asymptomatic	march 2019
434	Guanajuato	20.4082232	-101.6769241	Asymptomatic	march 2019

Sample No.	Sampling location	Latitude[a]	Longitude[a]	Sampled plant health status[b]	Sampling date
435	Guanajuato	20.4082232	-101.6769241	Asymptomatic	march 2019
436	Guanajuato	20.4082232	-101.6769241	Symptomatic	march 2019
437	Guanajuato	20.4082232	-101.6769241	Symptomatic	march 2019
438	Guanajuato	20.4082232	-101.6769241	Asymptomatic	march 2019
439	Guanajuato	20.4082232	-101.6769241	Asymptomatic	march 2019
440	Guanajuato	20.4082232	-101.6769241	Symptomatic	march 2019
441	Guanajuato	20.4082232	-101.6769241	Symptomatic	march 2019
442	Guanajuato	20.4082232	-101.6769241	Symptomatic	march 2019
443	Guanajuato	20.4082232	-101.6769241	Symptomatic	march 2019
444	Guanajuato	20.4082232	-101.6769241	Symptomatic	march 2019
445	Guanajuato	20.4082232	-101.6769241	Asymptomatic	march 2019
446	Guanajuato	20.4082232	-101.6769241	Asymptomatic	march 2019
447	Guanajuato	20.4082232	-101.6769241	Asymptomatic	march 2019
448	Michoacan	ND	ND	Asymptomatic	march 2019
449	Michoacan	ND	ND	Asymptomatic	march 2019
450	Michoacan	ND	ND	Asymptomatic	march 2019
451	Michoacan	ND	ND	Asymptomatic	march 2019
452	Michoacan	ND	ND	Asymptomatic	march 2019
453	Michoacan	ND	ND	Asymptomatic	march 2019

Sample No.	Sampling location	Latitude[a]	Longitude[a]	Sampled plant health status[b]	Sampling date
454	Michoacan	ND	ND	Asymptomatic	march 2019
455	Michoacan	ND	ND	Asymptomatic	march 2019
456	Michoacan	ND	ND	Asymptomatic	march 2019
457	Michoacan	ND	ND	Asymptomatic	march 2019
458	Michoacan	ND	ND	Asymptomatic	march 2019
459	Michoacan	ND	ND	Asymptomatic	march 2019
460	Guanajuato	20.8687967	-101.3824085	Symptomatic	april 2019
461	Guanajuato	20.8687967	-101.3824085	Symptomatic	april 2019
462	Guanajuato	20.8687967	-101.3824085	Symptomatic	april 2019
463	Guanajuato	20.8687967	-101.3824085	Symptomatic	may 2019
464	Guanajuato	20.8687967	-101.3824085	Symptomatic	may 2019
465	Guanajuato	20.8687967	-101.3824085	Symptomatic	may 2019
466	Guanajuato	20.8687967	-101.3824085	Symptomatic	may 2019
467	Guanajuato	20.8687967	-101.3824085	Symptomatic	may 2019
468	Guanajuato	20.8687967	-101.3824085	Symptomatic	may 2019
469	Guanajuato	20.8687967	-101.3824085	Symptomatic	may 2019
470	Guanajuato	20.8687967	-101.3824085	Symptomatic	may 2019
471	Guanajuato	20.8687967	-101.3824085	Symptomatic	may 2019
472	Guanajuato	20.8687967	-101.3824085	Symptomatic	may 2019

Sample No.	Sampling location	Latitude[a]	Longitude[a]	Sampled plant health status[b]	Sampling date
473	Michoacan	20.2807836	-102.3695811	Asymptomatic	august 2019
474	Michoacan	20.2807836	-102.3695811	Asymptomatic	august 2019
475	Michoacan	20.2807836	-102.3695811	Asymptomatic	august 2019
476	Michoacan	20.2807836	-102.3695811	Asymptomatic	august 2019
477	Michoacan	20.2807836	-102.3695811	Symptomatic	august 2019
478	Michoacan	20.2807836	-102.3695811	Asymptomatic	august 2019
479	Michoacan	20.2807836	-102.3695811	Symptomatic	august 2019
480	Michoacan	20.2807836	-102.3695811	Symptomatic	august 2019
481	Michoacan	20.2807836	-102.3695811	Symptomatic	august 2019
482	Guanajuato	20.3726666	-101.2084539	Asymptomatic	october 2019
483	Guanajuato	20.3726666	-101.2084539	Asymptomatic	october 2019
484	Guanajuato	20.3726666	-101.2084539	Symptomatic	october 2019
485	Guanajuato	20.3726666	-101.2084539	Asymptomatic	october 2019
486	Guanajuato	20.3726666	-101.2084539	Asymptomatic	october 2019
487	Guanajuato	20.3726666	-101.2084539	Asymptomatic	october 2019
488	Guanajuato	20.3726666	-101.2084539	Asymptomatic	october 2019
489	Guanajuato	20.3726666	-101.2084539	Asymptomatic	october 2019
490	Guanajuato	20.3726666	-101.2084539	Symptomatic	october 2019
491	Guanajuato	20.3726666	-101.2084539	Asymptomatic	october 2019

Sample No.	Sampling location	Latitude[a]	Longitude[a]	Sampled plant health status[b]	Sampling date
492	Guanajuato	20.3726666	-101.2084539	Asymptomatic	october 2019
493	Guanajuato	20.3726666	-101.2084539	Asymptomatic	october 2019
494	Jalisco	20.3823711	-102.9600625	Asymptomatic	october 2019
495	Jalisco	20.3823711	-102.9600625	Asymptomatic	october 2019
496	Guanajuato	20.544026	-100.577302	Symptomatic	december 2019
497	Guanajuato	20.544026	-100.577302	Symptomatic	december 2019
498	Guanajuato	20.544026	-100.577302	Symptomatic	december 2019
499	Guanajuato	20.544026	-100.577302	Symptomatic	december 2019
500	Guanajuato	20.544026	-100.577302	Symptomatic	december 2019
501	Guanajuato	20.544026	-100.577302	Symptomatic	december 2019
502	Guanajuato	20.544026	-100.577302	Symptomatic	december 2019
503	Guanajuato	20.544026	-100.577302	Symptomatic	december 2019
504	Guanajuato	20.544026	-100.577302	Symptomatic	december 2019
505	Queretaro	20.6918424	-100.014606	Symptomatic	july 2020
506	Queretaro	20.6918424	-100.014606	Symptomatic	july 2020
507	Queretaro	20.6918424	-100.014606	Symptomatic	july 2020
508	Queretaro	20.6918424	-100.014606	Symptomatic	july 2020
509	Queretaro	20.6918424	-100.014606	Symptomatic	july 2020
510	Queretaro	20.6918424	-100.014606	Symptomatic	july 2020

Sample No.	Sampling location	Latitude[a]	Longitude[a]	Sampled plant health status[b]	Sampling date
511	Queretaro	20.6918424	-100.014606	Symptomatic	july 2020

[a]Exact sampling site coordinates were not registered.

[b]Sampled plants were registered as symptomatic when they presented symptoms resembling those caused by *C. michiganensis*' tomato bacterial canker.

Supplementary table S2. Wild tomato varieties samples and sites metadata.

Sample No.	Location name	Latitude	Longitude	Environment	Sampled plant health status	Sampling date
1	Comonfort	20.738847	-100.78811	Border of cultivated field	Healthy	7 august 2017
2	Comonfort	20.738971	-100.78818	Border of cultivated field	Healthy	7 august 2017
3	Comonfort	20.739249	-100.78317	Border of cultivated field	Healthy	7 august 2017
4	Comonfort	20.73925	-100.78314	Border of cultivated field	Healthy	7 august 2017
5	La Huerta	19.52086	-104.53456	Border of cultivated field	Healthy	9 august 2017
6	Autlán	19.80285	-104.37653	Border of cultivated field	Healthy	10 august 2017
7	Autlán	19.80403	-104.36251	Border of cultivated field	Healthy	10 august 2017
8	Autlán	19.80285	-104.37653	Border of cultivated field	Healthy	10 august 2017
9	Cruz del Palmar	20.96488	-100.83816	Border of cultivated field	Healthy	16 november 2017
10	Cruz del Palmar	20.96488	-100.83816	Border of cultivated field	Healthy	16 november 2017
11	Comonfort	20.7204	-100.78339	Border of cultivated field	Healthy	16 november 2017
12	Comonfort	20.718319	-100.78309	Border of cultivated field	Healthy	16 november 2017
13	Comonfort	20.718891	-100.78301	Border of cultivated field	Healthy	16 november 2017
14	Comonfort	20.719025	-100.78294	Border of cultivated field	Healthy	16 november 2017
15	Comonfort	20.719026	-100.78291	Border of cultivated field	Healthy	16 november 2017
16	Comonfort	20.719344	-100.78292	Border of cultivated field	Healthy	16 november 2017

Sample No.	Location name	Latitude	Longitude	Environment	Sampled plant health status	Sampling date
17	La Huerta	19.5214	-104.53553	Border of cultivated field	Healthy	28 november 2017
18	La Huerta	19.5214	-104.53553	Border of cultivated field	Healthy	28 november 2017
19	La Huerta	19.5214	-104.53553	Border of cultivated field	Healthy	28 november 2017
20	La Concha	19.49062	-104.56561	Rural community	Healthy	28 november 2017
21	La Concha	19.49062	-104.56561	Rural community	Healthy	28 november 2017
22	La Concha	19.49062	-104.56561	Rural community	Healthy	28 november 2017
23	La Concha	19.49062	-104.56561	Rural community	Healthy	28 november 2017
24	Aguacaliente	19.51084	-104.56929	Border of cultivated field	Healthy	28 november 2017
25	Aguacaliente	19.51084	-104.56929	Border of cultivated field	Healthy	28 november 2017
26	Aguacaliente	19.51084	-104.56929	Border of cultivated field	Healthy	28 november 2017
27	Aguacaliente	19.51084	-104.56929	Border of cultivated field	Healthy	28 november 2017
28	El Corcovado	19.83623	-104.30056	Close to irrigation canal	Healthy	29 november 2017
29	El Corcovado	19.83623	-104.30056	Close to irrigation canal	Healthy	29 november 2017
30	El Corcovado	19.83623	-104.30056	Close to irrigation canal	Healthy	29 november 2017
31	El Corcovado	19.83623	-104.30056	Close to irrigation canal	Healthy	29 november 2017
32	El Corcovado	19.83623	-104.30056	Close to irrigation canal	Healthy	29 november 2017
33	El Corcovado	19.83574	-104.29393	Close to irrigation canal	Healthy	29 november 2017
34	El Corcovado	19.83574	-104.29393	Close to irrigation canal	Healthy	29 november 2017

Sample No.	Location name	Latitude	Longitude	Environment	Sampled plant health status	Sampling date
35	El Corcovado	19.83574	-104.29393	Close to irrigation canal	Healthy	29 november 2017
36	El Corcovado	19.83574	-104.29393	Close to irrigation canal	Healthy	29 november 2017
37	El Corcovado	19.83574	-104.29393	Close to irrigation canal	Healthy	29 november 2017
38	Autlán	19.80285	-104.37653	Border of cultivated field	Healthy	10 august 2017
39	Comonfort	20.716455	-100.78643	Border of cultivated field	Healthy	16 november 2017

Supplementary table S3. *Clavibacter michiganensis* species genome database information.

No.	Strain	Country of origin	Isolation year
**1**	317	USA	2003
**2**	1217	Russia	2006
**3**	MX19-J12A	Mexico (Michoacan)	2019
**4**	MX10-E1	Mexico (Guanajuato)	2010
**5**	MX14-E106-3	Mexico (Guanajuato)	2014
**6**	MX14-E111	Mexico (Guanajuato)	2014
**7**	MX14-E112	Mexico (Guanajuato)	2014
**8**	MX15-E125H	Mexico (Guanajuato)	2015
**9**	MX15-E129B	Mexico (Guanajuato)	2015
**10**	MX15-E130I	Mexico (Guanajuato)	2015
**11**	MX10-E2	Mexico (Guanajuato)	2010
**12**	MX10-E4	Mexico (Guanajuato)	2010
**13**	MX11-E43	Mexico (Guanajuato)	2011
**14**	MX13-E79	Mexico (Guanajuato)	2013
**15**	MX11-E8	Mexico (Guanajuato)	2011
**16**	MX13-E87-6	Mexico (Guanajuato)	2013
**17**	MX11-E9	Mexico (Guanajuato)	2011
**18**	MX13-E93	Mexico (Guanajuato)	2013
**19**	MX13-E96-2	Mexico (Guanajuato)	2013
**20**	MX13-E96-1	Mexico (Guanajuato)	2013
**21**	MX13-E97-1	Mexico (Guanajuato)	2013
**22**	MX13-E99-1	Mexico (Guanajuato)	2013
**23**	MX15-L2A	Mexico (Zacatecas)	2015
**24**	MX15-L2B	Mexico (Zacatecas)	2015
**25**	MX15-L3A	Mexico (Zacatecas)	2015

No.	Strain	Country of origin	Isolation year
**26**	MX15-L3D	Mexico (Zacatecas)	2015
**27**	MX16-A2A	Mexico (Michoacan)	2016
**28**	MX16-A2B	Mexico (Michoacan)	2016
**29**	MX16-A3A	Mexico (Michoacan)	2016
**30**	MX16-A3B	Mexico (Michoacan)	2016
**31**	MX16-A4B	Mexico (Michoacan)	2016
**32**	MX16-A4C	Mexico (Michoacan)	2016
**33**	MX16-A5C	Mexico (Michoacan)	2016
**34**	ATCC_14456	Italy	1961
**35**	MX19-Z14B	Mexico (Guanajuato)	2019
**36**	CA00001	USA	2000
**37**	CA00002	USA	2000
**38**	CASJ002	USA	1999
**39**	CASJ006	USA	2002
**40**	CASJ008	USA	2002
**41**	CAYO001	USA	2001
**42**	CFBP_4999	Hungary	1957
**43**	CFBP1465	France	1975
**44**	CFBP1940	Spain	1979
**45**	CFBP2494	Algeria	1985
**46**	CFBP5842	Brazil	1993
**47**	CFBP6885	France	2004
**48**	CFBP7158	New Zealand	1968
**49**	CFBP7311	Morocco	1989
**50**	CFBP7312	China	1998
**51**	CFBP7314	USA	2002

No.	Strain	Country of origin	Isolation year
**52**	CFBP7315	USA	1998
**53**	CFBP7316	USA	1998
**54**	CFBP7488	France	2008
**55**	CFBP7568	USA	2000
**56**	CFBP7589	Belgium	1984
**57**	CMM09	Mexico (Michoacan)	2015
**58**	CMM84	Mexico (Sinaloa)	2018
**59**	MX15-G23M	Mexico (Nuevo Leon)	2015
**60**	MX15-G23O	Mexico (Nuevo Leon)	2015
**61**	MX15-G23P	Mexico (Nuevo Leon)	2015
**62**	MX15-G23Q	Mexico (Nuevo Leon)	2015
**63**	MX16-H6A	Mexico (Queretaro)	2016
**64**	MX16-H8B	Mexico (Queretaro)	2016
**65**	MX16-I10B	Mexico (Guanajuato)	2016
**66**	MX16-I10C	Mexico (Guanajuato)	2016
**67**	MX16-I12A	Mexico (Guanajuato)	2016
**68**	MX16-I12B	Mexico (Guanajuato)	2016
**69**	MX16-I12C	Mexico (Guanajuato)	2016
**70**	MX19-I18A	Mexico (Guanajuato)	2019
**71**	MX19-I22A	Mexico (Guanajuato)	2019
**72**	MX19-I22B	Mexico (Guanajuato)	2019
**73**	MX16-I12D	Mexico (Guanajuato)	2016
**74**	MX15-112	Mexico (Guanajuato)	2015
**75**	MX15-115	Mexico (Guanajuato)	2015
**76**	MX15-113	Mexico (Guanajuato)	2015
**77**	MX15-212	Mexico (Guanajuato)	2015

No.	Strain	Country of origin	Isolation year
**78**	MAI1001	Uruguay	2012
**79**	MAI1009	Uruguay	2012
**80**	MAI1050	Uruguay	2014
**81**	MX15-M3A	Mexico (Aguascalientes)	2015
**82**	MX15-M3B	Mexico (Aguascalientes)	2015
**83**	MX15-M3C	Mexico (Aguascalientes)	2015
**84**	MX15-M3C2	Mexico (Aguascalientes)	2015
**85**	MX15-M3D	Mexico (Aguascalientes)	2015
**86**	MSF322	Chile	2005
**87**	NCPPB_382	UK	1956
**88**	NT20-18	the Netherlands	2020
**89**	NT20-20	the Netherlands	2020
**90**	NT20-5-15	the Netherlands	2020
**91**	MX16-N32A	Mexico (Colima)	2016
**92**	MX16-N32B	Mexico (Colima)	2016
**93**	MX16-O5B	Mexico (Guanajuato)	2016
**94**	MX16-O5C	Mexico (Guanajuato)	2016
**95**	OP3	Chile	2015
**96**	MX16-P35C	Mexico (Guanajuato)	2016
**97**	MX17-R2C	Mexico (Jalisco)	2017
**98**	MX18-R6C	Mexico (Guanajuato)	2018
**99**	MX15-S3C	Mexico (Michoacan)	2015
**100**	MX15-S3F	Mexico (Michoacan)	2015
**101**	MX16-S2D	Mexico (Guanajuato)	2016
**102**	UF1	USA	2012
**103**	NT20-V1	the Netherlands	2020

No.	Strain	Country of origin	Isolation year
**104**	NT20-V10	the Netherlands	2020
**105**	NT20-V12	the Netherlands	2020
**106**	NT20-V3-1	the Netherlands	2020
**107**	NT20-V5-1	the Netherlands	2020
**108**	NT20-V8	the Netherlands	2020
**109**	VKM_Ac_1790	Russia	1993
**110**	VL527	Chile	2012
**111**	MX17-V20C	Mexico (Queretaro)	2017
**112**	MX17-V56C	Mexico (Queretaro)	2017
**113**	MX16-V9A	Mexico (Queretaro)	2016
**114**	MX16-V9C	Mexico (Queretaro)	2016
**115**	VQ143	Chile	2000
**116**	VQ28	Chile	1996
**117**	MX16-W	Mexico (Guanajuato)	2016
**118**	Z001	USA	2012
**119**	Z002	USA	2012

Supplementary table S4. *Clavibacter* genus genome database information. *Cm, C. michiganensis; C sp., Clavibacter* sp.; *Ccal, C. californiensis*; *Csep*, *C. sepedonicus*; *Cneb*, *C. nebraskensis*; *Cins, C. insidiosus*; *Cphas*, *C. phaseoli*; *Czhang, C. zhanzhiyongii*; *Ccap, C. capsici*; *Ctes, C. tessellarius*.

No.	id	Isolation place	Isolation year	Host	Associated publication	BioSample ID
**1**	Ccap RA1B	Mexico	2017	tomato (*Solanum lycopersicum*)	this study	SAMN36730407
**2**	Cins CFBP 2404	USA	1955	alfalfa (*Medicago sativa*)	-	SAMN10256267
**3**	Cneb 61-1	USA	2006	maize (*Zea mays*)	-	SAMN10388288
**4**	Cneb 7580	USA	2006	maize (*Zea mays*)	-	SAMN10388289
**5**	Cm CFBP7158	New Zealand	1968	tomato (*Solanum lycopersicum*)	(1)	SAMN09428057
**6**	Cm CFBP5842	Brazil	1993	pepper (*Capsicum annum*)	(1)	SAMN09428033
**7**	Cm CFBP7568	USA	2000	tomato (*Solanum lycopersicum*)	(1)	SAMN09427737
**8**	Cm CFBP7314	USA	2002	tomato (*Solanum lycopersicum*)	(1)	SAMN09427551
**9**	Cm CFBP7315	USA	1998	tomato (*Solanum lycopersicum*)	(1)	SAMN09427636
**10**	Cm ATCC 14456	Italy	1961	tomato (*Solanum lycopersicum*)	(1)	SAMN09427889
**11**	Cm NZ2541	UK	1962	tomato (*Solanum lycopersicum*)	(1)	SAMN09427815
**12**	Cm NT20-18	the Netherlands	2020	tomato (*Solanum lycopersicum*)	this study	SAMN36730448
**13**	Ccap 1207	South Korea	1997	pepper (*Capsicum annum*)	-	SAMN13899376
**14**	Cm OP3	Chile	2015	tomato (*Solanum lycopersicum*)	(2)	SAMN13567269
**15**	Cm VL527	Chile	2012	tomato (*Solanum lycopersicum*)	(2)	SAMN13567270
**16**	Cm MSF322	Chile	2005	tomato (*Solanum lycopersicum*)	(2)	SAMN13567268
**17**	Czhang DM1	Australia	2017	barley (*Hordeum vulgare*)	(3)	SAMN15913044
**18**	C sp LMG 26808	the Netherlands	unknown	tomato (*Solanum lycopersicum*)	(4)	SAMN02951915
**19**	Cm MX14-E106-3	Mexico	2014	tomato (*Solanum lycopersicum*)	this study	SAMN36730460
**20**	Cm MX13-E96-2	Mexico	2013	tomato (*Solanum lycopersicum*)	this study	SAMN36730461
**21**	Cm MX16-H8B	Mexico	2016	tomato (*Solanum lycopersicum*)	this study	SAMN36730462
**22**	Cm MX16-I12A	Mexico	2016	tomato (*Solanum lycopersicum*)	this study	SAMN36730463

No.	id	Isolation place	Isolation year	Host	Associated publication	BioSample ID
**23**	Cm MX15-L3A	Mexico	2015	tomato (*Solanum lycopersicum*)	this study	SAMN36730464
**24**	Cm MX15-115	Mexico	2015	tomato (*Solanum lycopersicum*)	this study	SAMN36730465
**25**	Cm CASJ006	USA	2002	tomato (*Solanum lycopersicum*)	(5)	SAMN05439583
**26**	Cm CASJ009	USA	2011	tomato (*Solanum lycopersicum*)	(5)	SAMN05439586
**27**	Cm CFBP8019	the Netherlands	unknown	tomato (*Solanum lycopersicum*)	(5)	SAMN05559657
**28**	Cm CFBP7494	Chile	1999	tomato (*Solanum lycopersicum*)	(5)	SAMN05559655
**29**	Cm CFBP8017	the Netherlands	2006	tomato (*Solanum lycopersicum*)	(5)	SAMN05559658
**30**	Cm CASJ008	USA	2002	tomato (*Solanum lycopersicum*)	(5)	SAMN05439587
**31**	Cm Z002	USA	2012	orchard grass (*Dactylis glomerata*)	(6)	SAMN08438829
**32**	Cm AY1B3	USA	2014	perennial ryegrass (*Lolium perenne*)	(6)	SAMN08438834
**33**	Cm AY1A6	USA	2014	perennial ryegrass (*Lolium perenne*)	(6)	SAMN08438832
**34**	Cm AY1B2	USA	2013	perennial ryegrass (*Lolium perenne*)	(6)	SAMN08438833
**35**	Cm Z001	USA	2012	orchard grass (*Dactylis glomerata*)	(6)	SAMN08438828
**36**	Cins R1-1	USA	2009	barrel medic (*Medicago truncatula*)	(7)	SAMN03397809
**37**	Csep ATCC33113	Canada	1968	potato (*Solanum tuberosum*)	(8)	SAMEA1705948
**38**	Ccap PF008	South Korea	1999	pepper (*Capsicum annum*)	(9)	SAMN03896004
**39**	Cneb NCPPB 2581	USA	1971	maize (*Zea mays*)	-	SAMEA2271949
**40**	Cneb DOAB 395	Canada	2014	maize (*Zea mays*)	-	SAMN04488295
**41**	Ctes DOAB 609	USA	1976	wheat (*Triticum aestivum*)	-	SAMN04390115
**42**	Ctes ATCC 33566	USA	1978	wheat (*Triticum aestivum*)	(10)	SAMN11854497
**43**	Cins LMG 3663	USA	1955	alfalfa (*Medicago sativa*)	(10)	SAMN06579200
**44**	Cins R1-3	USA	2009	barrel medic (*Medicago truncatula*)	-	SAMN05441568
**45**	Csep CFIA-Cs3N	Canada	1976	potato (*Solanum tuberosum*)	(10)	SAMN06579179
**46**	Csep CFIA-CsR14	Canada	1991	potato (*Solanum tuberosum*)	(10)	SAMN06579198
**47**	Cm NCPPB 382	UK	1956	tomato (*Solanum lycopersicum*)	(11)	SAMEA3138263
**48**	Cm MAI1009	Uruguay	2012	tomato (*Solanum lycopersicum*)	this study	SAMN36730468

No.	id	Isolation place	Isolation year	Host	Associated publication	BioSample ID
**49**	Cm 1217	Russia	2006	potato (*Solanum tuberosum*)	-	SAMN14380869
**50**	Cm VKM Ac-1790	Russia	1993	Agrostis sp.	(12)	SAMN16617044
**51**	Cphas VKM Ac-2886	Russia	2017	red elderberry (*Sambucus racemosa*)	(12)	SAMN16617045
**52**	C sp VKM Ac-2542	Russia	1993	quackgrass (*Elymus repens*)	(12)	SAMN16617046
**53**	C sp VKM Ac-2872	USA	2020	annual bluegrass (*Poa annua*)	(12)	SAMN16617047
**54**	C sp VKM Ac-2873	USA	2020	common bent (*Agrostis capillaris*)	(12)	SAMN16617048
**55**	C sp PvP097	USA	unknown	switchgrass (*Panicum virgatum*)	-	SAMN17620029
**56**	C sp PvP098	USA	unknown	switchgrass (*Panicum virgatum*)	-	SAMN17620062
**57**	C sp PvP036	USA	unknown	switchgrass (*Panicum virgatum*)	-	SAMN18251569
**58**	C sp CFBP 3399	the Netherlands	1987	*Tulipa* sp.	-	SAMN19287682
**59**	Cm VQ143	Chile	2000	tomato (*Solanum lycopersicum*)	-	SAMN19515693
**60**	Cm VQ28	Chile	1996	tomato (*Solanum lycopersicum*)	-	SAMN19515692
**61**	Cm 0317	USA	2003	tomato (*Solanum lycopersicum*)	-	SAMN10686905
**62**	Cm CMM84	Mexico	2018	tomato (*Solanum lycopersicum*)	-	SAMN22209808
**63**	C sp LMG7333	Hungary	1957	tomato (*Solanum lycopersicum*)	-	SAMN20474849
**64**	Cchil CFBP 8217	Chile	2007	tomato (*Solanum lycopersicum*)	-	SAMN11834755
**65**	Cphas LPPA 982	Spain	2009	bean (*Phaseolus vulgaris*)	-	SAMN11855613
**66**	C sp A6099	India	2013	tomato (*Solanum lycopersicum*)	-	SAMN20285765
**67**	Ccal CFBP 8216	USA	2000	tomato (*Solanum lycopersicum*)	-	SAMN11842393
**68**	Cm MAI1001	Uruguay	2012	tomato (*Solanum lycopersicum*)	this study	SAMN36730467
**69**	Cm MAI1050	Uruguay	2014	tomato (*Solanum lycopersicum*)	this study	SAMN36730469

Supplementary table S5. *Clavibacter michiganensis* genes from gene families identified by the phylogenetic and pangenomic analyses. Genes are ordered according to their occurrence in *C. michiganensis* strain NCPPB 382's genome.

Gene No. [a]	Gene family	Present in	Genomic location	Loci name or number	Pfam accession number	Function (Pfam)	Function (RASTtk) [b]	Accession number
**1**	1	Cm subclade	chromosome	PAI	-	-	-	WP_246930142.1
**2**	2	Bcm clade	chromosome	PAI	-	-	-	WP_128517022.1
**3**	3	Cm subclade	chromosome	PAI	-	-	ORF19	WP_011931265.1
**4**	4	Cm subclade	chromosome	PAI	-	-	-	WP_143330934.1
**5**	5	Cm subclade	chromosome	PAI	-	-	-	WP_043560329.1
**6**	6	Cm subclade	chromosome	PAI	-	-	ParB domain-containing protein nuclease	KAF0257781.1
**7**	7	Bcm clade	chromosome	PAI	PF08843.14	Nucleotidyl transferase AbiEii toxin, Type IV TA system	-	WP_011931273.1
**8**	8	Bcm clade	chromosome	PAI	PF13338.9	Transcriptional regulator, AbiEi antitoxin	-	WP_174239385.1
**9**	9	Cm subclade	chromosome	PAI	-	-	putative secreted protein	WP_011931290.1
**10**	10	Cm subclade	chromosome	PAI	-	-	-	WP_043560372.1
**11**	11	Cm subclade	chromosome	PAI	-	-	-	WP_086506682.1
**12**	12	Cm subclade	chromosome	PAI	PF00106.28	short chain dehydrogenase	Short-chain dehydrogenase	WP_011931300.1
**13****	13	Cm subclade	chromosome	PAI	PF00933.24	Glycosyl hydrolase family 3 N terminal domain	beta-glucosidase (EC 3.2.1.21)	WP_011931301.1
**14**	14	Cm subclade	chromosome	PAI	PF00528.25	Binding-protein-dependent transport system inner membrane component	Multiple sugar transport system permease protein	WP_011931303.1
**15**	15	Cm subclade	chromosome	PAI	PF13416.9	Bacterial extracellular solute-binding protein	ABC transporter, substrate-binding protein (cluster 1, maltose/g3p/polyamine/iron)	WP_011931305.1
**16**	16	Cm subclade	chromosome	PAI	PF00440.26	Bacterial regulatory proteins, tetR family	Transcriptional regulator, AcrR family	WP_011931306.1
**17**	17	Cm subclade	chromosome	PAI	PF00232.21	Glycosyl hydrolase family 1	beta-glucosidase (EC 3.2.1.21)	WP_172405778.1
**18**	18	Cm subclade	chromosome	PAI	PF13377.9	Periplasmic binding protein-like domain	Transcriptional regulator, LacI family	KAF0259824.1
**19**	19	Cm subclade	chromosome	PAI	PF00331.23	Glycosyl hydrolase family 10	Endo-1,4-beta-xylanase (EC 3.2.1.8)	WP_011931309.1
**20****	20	Cm subclade	chromosome	PAI	PF00072.27	Response regulator receiver domain	Two-component transcriptional response regulator, LuxR family	WP_011931310.1
**21**	21	Cm subclade	chromosome	PAI	PF07730.16	Histidine kinase	Two-component system sensor histidine kinase	OUE17732.1
**22**	22	Cm subclade	chromosome	PAI	PF03176.18	MMPL family	Integral membrane protein	WP_011931312.1

Gene No. [a]	Gene family	Present in	Genomic location	Loci name or number	Pfam accession number	Function (Pfam)	Function (RASTtk) [b]	Accession number
**23**	23	Cm subclade	chromosome	PAI	PF00067.25	Cytochrome P450	Putative cytochrome P450 hydroxylase	WP_197535362.1
**24**	24	Cm subclade	chromosome	PAI	PF13370.9	4Fe-4S single cluster domain of Ferredoxin I	Ferredoxin-like protein SCO7676	WP_011931314.1
**25****	25	Cm subclade	chromosome	PAI	PF07992.17	Pyridine nucleotide-disulphide oxidoreductase	Ferredoxin reductase	WP_050976273.1
**26**	26	Cm subclade	chromosome	PAI	PF18120.4	Domain of unknown function (DUF5597)	beta-galactosidase (EC 3.2.1.23)	WP_011931321.1
**27**	27	Cm subclade	chromosome	PAI	PF04616.17	Glycosyl hydrolases family 43	Xylan 1,4-beta-xylosidase (EC 3.2.1.37)	WP_206601424.1
**28****	28	Cm subclade	chromosome	PAI	PF07690.19	Major Facilitator Superfamily	Uncharacterized MFS-type transporter	OUD87863.1
**29**	29	Cm subclade	chromosome	PAI	PF17389.5	Bacterial alpha-L-rhamnosidase 6 hairpin glycosidase domain	alpha-L-rhamnosidase (EC 3.2.1.40)	WP_011931324.1
**30**	30	Outside Bcm	chromosome	-	PF01613.21	Flavin reductase like domain	-	WP_011931381.1
**31**	31	Bcm clade	chromosome	-	-	-	-	-
**32**	32	Bcm clade	chromosome	-	-	-	-	WP_158206943.1
**33**	33	Outside Bcm	chromosome	1	PF07690.19	Major Facilitator Superfamily	Uncharacterized MFS-type transporter	WP_011931475.1
**34****	34	Cm subclade	chromosome	1	-	-	-	WP_011931476.1
**35**	35	Outside Bcm	chromosome	1	PF13411.9	MerR HTH family regulatory protein	Regulatory protein MerR	WP_011931477.1
**36**	36	Outside Bcm	chromosome	2	PF13377.9	Periplasmic binding protein-like domain	Transcriptional regulator, LacI family	WP_011931578.1
**37****	37	Outside Bcm	chromosome	2	PF13416.9	Bacterial extracellular solute-binding protein	ABC transporter, substrate-binding protein (cluster 1, maltose/g3p/polyamine/iron)	WP_011931579.1
**38**	38	Outside Bcm	chromosome	2	PF00528.25	Binding-protein-dependent transport system inner membrane component	Possible alpha-xyloside ABC transporter, permease component	WP_011931580.1
**39**	39	Outside Bcm	chromosome	2	PF01055.29	Glycosyl hydrolases family 31	alpha-xylosidase (EC 3.2.1.177)	WP_011931582.1
**40**	40	Outside Bcm	chromosome	-	PF13620.9	Carboxypeptidase regulatory-like domain	Probable hemagglutinin/hemolysin-related protein	WP_011931651.1
**41**	41	Outside Bcm	chromosome	-	-	-	-	WP_011931662.1
**42**	42	Outside Bcm	chromosome	-	-	-	-	WP_011931676.1
**43**	43	Outside Bcm	chromosome	-	-	-	-	WP_011931729.1
**44**	44	Cm subclade	chromosome	-	-	-	-	WP_011931730.1
**45**	45	Cm subclade	chromosome	3	PF11706.11	CGNR zinc finger	-	WP_011931760.1
**46****	46	Cm subclade	chromosome	3	PF02627.23	Carboxymuconolactone decarboxylase family	4-carboxymuconolactone decarboxylase (EC 4.1.1.44)	WP_011931761.1

Gene No. [a]	Gene family	Present in	Genomic location	Loci name or number	Pfam accession number	Function (Pfam)	Function (RASTtk) [b]	Accession number
**47**	47	Cm subclade	chromosome	3	PF02126.21	Phosphotriesterase family	-	WP_011931762.1
**48**	48	Cm subclade	chromosome	-	-	-	-	WP_011931779.1
**49**	49	Outside Bcm	chromosome	-	PF00107.29	Zinc-binding dehydrogenase	Putative oxidoreductase	WP_011931798.1
**50**	50	Cm subclade	chromosome	-	-	-	-	CAN00658.1
**51**	51	Cm subclade	chromosome	-	-	-	-	WP_043560529.1
**52****	52	Cm subclade	chromosome	4	PF01636.26	Phosphotransferase enzyme family	-	WP_043560553.1
**53**	53	Cm subclade	chromosome	4	PF13302.10	Acetyltransferase (GNAT) domain	-	WP_011931899.1
**54**	52	Cm subclade	chromosome	4	PF00501.31	AMP-binding enzyme	Long-chain-fatty-acid--CoA ligase (EC 6.2.1.3)	WP_094106685.1
**55**	54	Cm subclade	chromosome	4	PF00109.29	Beta-ketoacyl synthase, N-terminal domain	3-oxoacyl-[acyl-carrier-protein] synthase, KASII (EC 2.3.1.179)	WP_011931901.1
**56**	55	Cm subclade	chromosome	4	PF02776.21	Thiamine pyrophosphate enzyme, N-terminal TPP binding domain	-	WP_011931902.1
**57**	56	Cm subclade	chromosome	4	PF02441.22	Flavoprotein	Phosphopantothenoylcysteine decarboxylase (EC 4.1.1.36) homolog	WP_011931903.1
**58**	56	Cm subclade	chromosome	4	PF00005.30	ABC transporter	ABC-type antimicrobial peptide transport system, ATPase component	WP_011931904.1
**59**	57	Cm subclade	chromosome	4	PF02687.24	FtsX-like permease family	ABC transporter, permease protein	WP_011931905.1
**60**	58	Cm subclade	chromosome	4	-	-	-	WP_158309428.1
**61**	59	Cm subclade	chromosome	4	PF11139.11	Sap, sulfolipid-1-addressing protein	-	WP_011931906.1
**62**	60	Bcm clade	chromosome	-	-	-	-	WP_011931913.1
**63**	61	Bcm clade	chromosome	-	-	-	-	-
**64**	62	Outside Bcm	chromosome	-	-	-	-	WP_043560623.1
**65**	63	Bcm clade	chromosome	-	-	-	-	KAF0258211.1
**66**	64	Cm subclade	chromosome	-	-	-	-	WP_153259845.1
**67**	65	Bcm clade	chromosome	-	-	-	-	WP_227267107.1
**68**	66	Outside Bcm	chromosome	-	-	-	-	WP_012037897.1
**69**	67	Outside Bcm	chromosome	-	-	-	-	CAN01270.1
**70**	68	Bcm clade	chromosome	-	-	-	-	WP_128516978.1

Gene No. [a]	Gene family	Present in	Genomic location	Loci name or number	Pfam accession number	Function (Pfam)	Function (RASTtk) [b]	Accession number
**71**	69	Outside Bcm	chromosome	-	PF08378.14	Nuclease-related domain	-	WP_043560772.1
**72**	70	Cm subclade	chromosome	-	-	-	-	OUD82947.1
**73**	71	Cm subclade	chromosome	-	-	-	-	CAN01728.1
**74**	72	Bcm clade	chromosome	-	-	-	-	MWJ34252.1
**75**	73	Cm subclade	chromosome	-	-	-	-	WP_043560818.1
**76**	74	Cm subclade	chromosome	-	-	-	-	MWJ79166.1
**77**	75	Outside Bcm	chromosome	5	PF12730.10	ABC-2 family transporter protein	-	WP_012038649.1
**78**	76	Outside Bcm	chromosome	5	PF12730.10	ABC-2 family transporter protein	putative transporter, trans-membrane domain bacteriocin immunity protein	WP_231840595.1
**79**	77	Outside Bcm	chromosome	5	PF00072.27	Response regulator receiver domain	Two-component transcriptional response regulator, LuxR family	WP_012038652.1
**80**	78	Outside Bcm	chromosome	5	PF07730.16	Histidine kinase	two-component sensor	WP_012038653.1
**81**	79	Outside Bcm	chromosome	5	-	-	-	WP_012038654.1
**82****	80	Outside Bcm	chromosome	5	PF13575.9	Domain of unknown function (DUF4135)	Lanthionine biosynthesis protein LanM	WP_012038655.1
**83**	81	Bcm clade	chromosome	-	-	-	Uncharacterized 29.3 kDa protein (ORF92)	WP_012038733.1
**84**	82	Outside Bcm	chromosome	-	-	-	-	OUD85637.1
**85**	83	Outside Bcm	chromosome	6	-	-	-	CAN02169.1
**86**	84	Cm subclade	chromosome	6	-	-	-	OUD81445.1
**87**	85	Bcm clade	chromosome	6	-	-	-	OUD86580.1
**88**	86	Cm subclade	chromosome	-	PF01823.22	MAC/Perforin domain	-	WP_012039071.1
**89**	87	Outside Bcm	chromosome	-	-	-	-	WP_012039113.1
**90**	88	Outside Bcm	chromosome	-	PF13310.9	Virulence protein RhuM family	Putative DNA-binding protein in cluster with Type I restriction-modification system	WP_012039383.1
**91**	89	Outside Bcm	chromosome	-	-	-	-	WP_012039438.1
**92**	90	Outside Bcm	chromosome	7	PF00144.27	Beta-lactamase	Putative esterase	WP_012039468.1
**93****	91	Outside Bcm	chromosome	7	PF00251.23	Glycosyl hydrolases family 32 N-terminal domain	Sucrose-6-phosphate hydrolase (EC 3.2.1.26)	WP_012039469.1
**94**	92	Outside Bcm	chromosome	7	PF00528.25	Binding-protein-dependent transport system inner membrane component	ABC transporter, permease protein 2 (cluster 1, maltose/g3p/polyamine/iron)	WP_012039470.1

Gene No. [a]	Gene family	Present in	Genomic location	Loci name or number	Pfam accession number	Function (Pfam)	Function (RASTtk) [b]	Accession number
**95**	93	Outside Bcm	chromosome	7	PF00528.25	Binding-protein-dependent transport system inner membrane component	ABC transporter, permease protein 1 (cluster 1, maltose/g3p/polyamine/iron)	WP_012039471.1
**96**	94	Outside Bcm	chromosome	7	PF01547.28	Bacterial extracellular solute-binding protein	ABC transporter, substrate-binding protein (cluster 1, maltose/g3p/polyamine/iron)	WP_012039472.1
**97**	95	Outside Bcm	chromosome	7	PF13377.9	Periplasmic binding protein-like domain	Transcriptional regulator, LacI family	WP_012039473.1
**98**	96	Outside Bcm	chromosome	-	-	-	-	OUD86960.1
**99**	97	Outside Bcm	chromosome	-	PF01425.24	Amidase	Allophanate hydrolase (EC 3.5.1.54)	WP_012039512.1
**100**	98	Bcm clade	chromosome	-	-	-	-	WP_012039545.1
**101**	99	Cm subclade	chromosome	8	-	-	GO:0010954: positive regulation of protein processing, GO:1901565: organonitrogen compound catabolic process	WP_012039590.1
**102**	100	Cm subclade	chromosome	8	PF00005.30	ABC transporter	GO:0005524: ATP binding GO:0005886: plasma membrane	WP_012039591.1
**103****	101	Cm subclade	chromosome	8	-	-	GO:0022889: serine transmembrane transporter activity GO:0005886: plasma membrane	WP_012039592.1
**104**	102	Cm subclade	chromosome	8	-	-	GO:0016021: integral component of membrane GO:0030416: methylamine metabolic process	WP_012039593.1
**105**	103	Bcm clade	chromosome	-	-	-	-	OUD90117.1
**106**	1	Cm subclade	pCM2	-	-	-	-	CAM98530.1
**107**	7	Bcm clade	pCM2	-	PF08843.14	Nucleotidyl transferase AbiEii toxin, Type IV TA system	-	WP_011931205.1
**108**	8	Bcm clade	pCM2	-	PF13338.9	Transcriptional regulator, AbiEi antitoxin	-	WP_174239385.1

[a]Genes with ****** were used as a query in CORASON for homologous loci search.

[b]Underlined text indicates that RASTtk predicted function for these genes was substituted with ProteInfer function prediction.

Suppplementary table S6. Isolation data of organisms with confirmed presence of *C. michiganensis* loci homolog.

No.	Strain	Isolation year	Country of origin	Isolation source	BioSample ID	Locus found
**1**	*Actinomyces gerencseriae DSM 6844*	unknown	USA	*Homo sapiens*	SAMN02441199	5 (michiganin)
**2**	*Agreia bicolorata VKM Ac-1804*	2001	Russia	Soil	SAMN03333328	7
**3**	*Agreia bicolorata VKM Ac-2052*	unknown	Russia	*Calamagrostis neglecta*	SAMN06295879	8
**4**	*Agreia pratensis VKM Ac-2510*	unknown	Germany	Grasses phyllosphere	SAMN06296010	2, 3
**5**	*Agreia pratensis VKM Ac-2874*	2020	USA	*Poa annua*	SAMN16617043	2, 3, 8
**6**	*Agreia sp. COWG*	unknown	France	Acid mine drainage	SAMEA7322744	7
**7**	*Amnibacterium flavum M8JJ-5*	2016	China	*Nerium indicum* Mill.	SAMN08984915	7
**8**	*Arthrobacter sp. 24S4-2*	2017	Antarctica	Soil	SAMN11525741	3
**9**	*Arthrobacter sp. B2I5*	unknown	USA	Wheat field	SAMN34353127	PAI
**10**	*Arthrobacter sp. ISL-69*	2017	Chile	Soil	SAMN18298984	4 (michivionin)
**11**	*Arthrobacter sp. NtRootA1*	2019	Japan	*Nicotiana tabacum*	SAMD00283280	PAI
**12**	*Arthrobacter sp. Soil761*	2013	Germany	*Arabidopsis thaliana*	SAMN04155665	PAI
**14**	*Candidatus Microbacterium colombiense MAG_1911*	unknown	Colombia	Decomposition metagenome	SAMN33481873	7
**15**	*Curtobacterium oceanosedimentum PL-347*	2019	Namibia	Nest material from social spider *Stegodyphus dumicola*	SAMN21907557	8
**16**	*Curtobacterium sp. JUb34*	2007	France	Rotting hawthorn fruits (*Crataegus* sp.)	SAMN10361602	8
**17**	*Curtobacterium sp. KBS0715*	2013	USA	Soil	SAMN11620951	8
**18**	*Curtobacterium sp. MCJR17_020*	2016	USA	Pine-oak forest leaf litter	SAMN09009058	8
**19**	*Curtobacterium sp. MCLR17_031*	2016	USA	Grassland leaf litter	SAMN09009040	8
**20**	*Curtobacterium sp. MCLR17_045*	2016	USA	Grassland leaf litter	SAMN09009049	8
**21**	*Curtobacterium sp. MCSS17_006*	2016	USA	Coastal saline lake leaf litter	SAMN09009065	8
**22**	*Curtobacterium sp. ME12*	2017	India	*Terminalia paniculata*	SAMEA7026319	8

No.	Strain	Isolation year	Country of origin	Isolation source	BioSample ID	Locus found
**23**	*Curtobacterium sp. MWU13-2055*	2013	USA	Cranberry bog	SAMN27103189	8
**24**	*Curtobacterium sp. VKM Ac-1393*	1993	Russia	*Calamagrostis* sp.	SAMN16617052	8
**25**	*Herbiconiux ginsengi CGMCC 4.3491*	unknown	China	*Panax ginseng*	SAMN05216554	7
**26**	*Herbiconiux sp. GW715_bin.41*	2018	USA	Groundwater	SAMN10720236	7
**27**	*Kineococcus radiotolerans SRS30216 ATCC BAA-149*	unknown	USA	contaminated river	SAMN02598259	PAI
**28**	*Leifsonia poae BS71*	2018	USA	Forest soil-inoculated soil microcosm	SAMN20129593	PAI
**29**	*Leucobacter massiliensis 122RC15*	2014	France	*Homo sapiens*	SAMN06481177	PAI
**30**	*Leucobacter musarum subsp. musarum CBX152*	2009	Cape Verde	*Caenorhabditis* sp.	SAMN02673065	PAI
**31**	*Luteimicrobium sp. SMAG_U13463*	unknown	USA	Soil	SAMN37942866	3
**32**	*Microbacterium arborescens*	unknown	unknown	unknown	NA	5 (michiganin)
**33**	*Microbacterium fluvii IDR2*	2019	Spain	38 m deep groundwater	SAMN15677150	PAI
**34**	*Microbacterium oleivorans F-B2*	2016	USA	Silty clay	SAMN11093716	PAI
**35**	*Microbacterium oxydans BEL4b*	2010	Belgium	*Brassica napus*	SAMN03266142	PAI
**36**	*Microbacterium sp. JZ37*	2014	Saudi Arabia	*Tribulus terrestris*	SAMN13011529	PAI
**37**	*Microbacterium sp. Mcb102*	2012	Canada	*Alnus incana* subsp. *rugosa*	SAMN26547295	PAI
**38**	*Microbacterium sp. Root166*	2013	Germany	*Arabidopsis thaliana*	SAMN04155747	PAI
**39**	*Microbacterium sp. Yaish 1*	2015	Oman	Soil	SAMN07453917	5 (michiganin)
**40**	*Micrococcus luteus R17*	2016	China	Gamma ray irradiated soil	SAMN08014137	4 (michivionin)
**41**	*Nocardia carnea 740803004*	unknown	unknown	unknown	SAMN34231285	PAI
**42**	*Paenarthrobacter histidinolovorans JCM 2520*	unknown	unknown	Soil	SAMD00245287	PAI
**43**	*Planomonospora alba JCM 9373*	1996	Sudan	Soil	SAMD00645412	PAI
**44**	*Plantibacter cousiniae Bi02*	2017	United Kingdom	Soil	SAMEA7892333	3

No.	Strain	Isolation year	Country of origin	Isolation source	BioSample ID	Locus found
**45**	*Plantibacter cousiniae VKM Ac-1787*	unknown	Russia	*Cousinia onopordioides*	SAMN06295973	3
**46**	*Plantibacter flavus AKF*	2019	Poland	Contaminated LB plates	SAMN33294072	3
**47**	*Plantibacter flavus CFBP13513*	2014	France	*Raphanus sativus* var. Flamboyant 5	SAMN09063410	3
**48**	*Plantibacter flavus DSM 14012*	unknown	Germany	Grasses phyllosphere	SAMN10363204	3
**49**	*Plantibacter flavus VKM Ac-2504*	unknown	Germany	Grasses phyllosphere	SAMN06295974	3
**50**	*Plantibacter sp. CFBP 8798*	2016	France	*Raphanus sativus*	SAMN16238397	3
**51**	*Plantibacter sp. Leaf314*	2013	Switzerland	*Arabidopsis thaliana*	SAMN04151723	3
**52**	*Plantibacter sp. ME-Dv--P-095*	unknown	USA	*Arabidopsis thaliana*	SAMN34123785	3
**53**	*Plantibacter sp. PA-3-X8*	2013	Antarctica	Marine sediments	SAMN10255631	3
**54**	*Plantibacter sp. T3*	unknown	unknown	*Ectocarpus subulatus*	SAMEA6080509	3
**55**	*Plantibacter sp. VKM Ac-1784*	unknown	Russia	*Elymus repens*	SAMN06295909	3
**56**	*Plantibacter sp. VKM Ac-2876*	2020	USA	*Poa annua*	SAMN16617064	3
**57**	*Plantibacter sp. VKM Ac-2885*	2017	Russia	*Festuca rubra*	SAMN16617066	3
**58**	*Pseudoclavibacter sp. VKM Ac-2888*	2000	Russia	*Tanacetum vulgare*	SAMN16617068	3
**59**	*Pseudoclavibacter terrae THG-MD12*	2012	China	Soil	SAMN12810922	PAI
**60**	*Pseudolysinimonas kribbensis JCM 16015*	2006	South Korea	Soil	SAMD00645300	7
**61**	*Pseudolysinimonas kribbensis NBRC 108894*	unknown	unknown	Soil	SAMD00582136	7
**62**	*Rathayibacter iranicus DSM 7484*	1966	Iran	*Triticum aestivum*	SAMN08438724	5 (michiganin)
**63**	*Rathayibacter iranicus FH154*	2003	Turkey	*Triticum aestivum*	SAMN08438722	5 (michiganin)
**64**	*Rathayibacter iranicus NCPPB 2253 VKM Ac-1602*	1966	Iran	*Triticum aestivum*	SAMN12724560	5 (michiganin)
**65**	*Rathayibacter iranicus TRS5*	2003	Turkey	*Triticum aestivum*	SAMN08438723	5 (michiganin)
**66**	*Rathayibacter sp. AY1A1*	2014	USA	*Lolium perenne*	SAMN08321535	2

No.	Strain	Isolation year	Country of origin	Isolation source	BioSample ID	Locus found
**67**	*Rathayibacter sp. AY1A2*	2013	USA	*Lolium perenne*	SAMN08321 536	2
**68**	*Rathayibacter sp. AY1A3*	2013	USA	*Lolium perenne*	SAMN08321 537	2
**69**	*Rathayibacter sp. AY1A4*	2015	USA	*Lolium perenne*	SAMN08322 058	2
**70**	*Rathayibacter sp. AY1A5*	2013	USA	*Lolium perenne*	SAMN08322 059	2
**71**	*Rathayibacter sp. AY1A7*	2013	USA	*Lolium perenne*	SAMN08322 068	2
**72**	*Rathayibacter sp. AY1B1*	2013	USA	*Dactylis glomerata*	SAMN08438 754	2
**73**	*Rathayibacter sp. AY1B4*	2015	USA	*Lolium multiflorum*	SAMN08438 755	2
**74**	*Rathayibacter sp. AY1B5*	2015	USA	*Lolium perenne*	SAMN08438 756	2
**75**	*Rathayibacter sp. AY1B6*	2014	USA	*Lolium perenne*	SAMN08438 757	2
**76**	*Rathayibacter sp. AY1B7*	2013	USA	*Lolium perenne*	SAMN08438 758	2
**77**	*Rathayibacter sp. AY1B8*	2014	USA	*Lolium perenne*	SAMN08438 759	2
**78**	*Rathayibacter sp. AY1C1*	2013	USA	*Lolium perenne*	SAMN08438 761	2
**79**	*Rathayibacter sp. AY1C2*	2015	USA	*Lolium perenne*	SAMN08438 762	2
**80**	*Rathayibacter sp. AY1C3*	2013	USA	*Lolium perenne*	SAMN08438 763	2
**81**	*Rathayibacter sp. AY1C4*	2013	USA	*Lolium perenne*	SAMN08438 764	2
**82**	*Rathayibacter sp. AY1C5*	2013	USA	*Lolium perenne*	SAMN08438 765	2
**83**	*Rathayibacter sp. AY1C6*	2013	USA	*Lolium perenne*	SAMN08438 766	2
**84**	*Rathayibacter sp. AY1C7*	2013	USA	*Lolium perenne*	SAMN08438 767	2
**85**	*Rathayibacter sp. AY1C9*	2013	USA	*Lolium perenne*	SAMN08438 769	2
**86**	*Rathayibacter sp. AY1D1*	2013	USA	*Lolium perenne*	SAMN08438 770	2
**87**	*Rathayibacter sp. AY1D2*	2013	USA	*Lolium perenne*	SAMN08438 771	2
**88**	*Rathayibacter sp. AY1D3*	2013	USA	*Lolium perenne*	SAMN08438 772	2

No.	Strain	Isolation year	Country of origin	Isolation source	BioSample ID	Locus found
**89**	*Rathayibacter sp. AY1D4*	2013	USA	*Lolium perenne*	SAMN08438773	2
**90**	*Rathayibacter sp. AY1D5*	2013	USA	*Lolium perenne*	SAMN08438774	2
**91**	*Rathayibacter sp. AY1D7*	2014	USA	*Lolium multiflorum*	SAMN08438776	2
**92**	*Rathayibacter sp. AY1D9*	2013	USA	*Lolium perenne*	SAMN08438778	2
**93**	*Rathayibacter sp. AY1E1*	2013	USA	*Lolium perenne*	SAMN08438779	2
**94**	*Rathayibacter sp. AY1E2*	2014	USA	*Lolium perenne*	SAMN08438780	2
**95**	*Rathayibacter sp. AY1E3*	2014	USA	*Lolium perenne*	SAMN08438781	2
**96**	*Rathayibacter sp. AY1E4*	2013	USA	*Lolium perenne*	SAMN08438782	2
**97**	*Rathayibacter sp. AY1E5*	2015	USA	*Lolium multiflorum*	SAMN08438783	2
**98**	*Rathayibacter sp. AY1E6*	2014	USA	*Lolium multiflorum*	SAMN08438784	2
**99**	*Rathayibacter sp. AY1E8*	2015	USA	*Lolium perenne*	SAMN08438786	2
**100**	*Rathayibacter sp. AY1E9*	2013	USA	*Lolium perenne*	SAMN08438787	2
**101**	*Rathayibacter sp. AY1F2*	2013	USA	*Lolium perenne*	SAMN08438788	2
**102**	*Rathayibacter sp. AY1F3*	2013	USA	*Lolium perenne*	SAMN08438789	2
**103**	*Rathayibacter sp. AY1F4*	2014	USA	*Agrostis stolonifera*	SAMN08438790	2
**104**	*Rathayibacter sp. AY1F6*	2013	USA	*Lolium perenne*	SAMN08438791	2
**105**	*Rathayibacter sp. AY1F7*	2013	USA	*Lolium perenne*	SAMN08438792	2
**106**	*Rathayibacter sp. AY1F8*	2014	USA	*Lolium perenne*	SAMN08438793	2
**107**	*Rathayibacter sp. AY1F9*	2013	USA	*Lolium perenne*	SAMN08438794	2
**108**	*Rathayibacter sp. AY1G1*	2013	USA	*Lolium perenne*	SAMN08438795	2
**109**	*Rathayibacter sp. AY1G9*	2013	USA	*Lolium perenne*	SAMN08438798	2
**110**	*Rathayibacter sp. AY1H2*	2014	USA	*Agrostis stolonifera*	SAMN08438799	2

No.	Strain	Isolation year	Country of origin	Isolation source	BioSample ID	Locus found
**111**	*Rathayibacter sp. AY1H3*	2014	USA	*Dactylis glomerata*	SAMN08438 800	2
**112**	*Rathayibacter sp. AY2B1*	2014	USA	*Agrostis stolonifera*	SAMN08438 813	2
**113**	*Rathayibacter sp. AY2B3*	2013	USA	*Lolium perenne*	SAMN08438 814	2
**114**	*Rathayibacter sp. AY2B5*	2013	USA	*Lolium perenne*	SAMN08438 816	2
**115**	*Rathayibacter sp. AY2B7*	2015	USA	*Agrostis stolonifera*	SAMN08438 818	2
**116**	*Rathayibacter sp. AY2B9*	2014	USA	*Agrostis stolonifera*	SAMN08438 819	2
**117**	*Rathayibacter sp. Leaf248*	2013	Switzerland	*Arabidopsis thaliana*	SAMEA9963 710	2
**118**	*Rathayibacter sp. Leaf299*	2013	Switzerland	*Arabidopsis thaliana*	SAMN04151 718	PAI
**119**	*Rathayibacter sp. PhB127*	unknown	unknown	unknown	SAMN10361 286	2
**120**	*Rathayibacter sp. PhB152*	unknown	unknown	unknown	SAMN10361 294	2
**121**	*Rathayibacter sp. RFBD1*	unknown	unknown	unknown	SAMN08438 752	2
**122**	*Rathayibacter sp. VKM Ac-2759*	2000	Russia	*Tanacetum vulgare*	SAMN12724 562	2
**123**	*Rathayibacter sp. VKM Ac-2762*	2001	Russia	*Limonium* sp.	SAMN12724 565	2
**124**	*Rathayibacter sp. VKM Ac-2801*	2017	Russia	*Androsace villosa* subsp. *koso-poljanskii*	SAMN12724 566	2
**125**	*Rathayibacter sp. VKM Ac-2805*	2017	Russia	*Gypsophila altissima*	SAMN12724 570	2
**126**	*Rathayibacter tritici ATCC 11402*	unknown	India	*Triticum aestivum*	SAMN08438 808	5 (michiganin)
**127**	*Rathayibacter tritici DSM 7486*	unknown	Egypt	*Triticum aestivum*	SAMN08438 750	5 (michiganin)
**128**	*Rathayibacter tritici GSPB 2748*	unknown	Pakistan	unknown	SAMN08438 806	5 (michiganin)
**129**	*Rathayibacter tritici GSPB 2752*	unknown	Pakistan	unknown	SAMN08438 807	5 (michiganin)
**130**	*Rathayibacter tritici ICMP 2628*	1966	Iran	*Triticum aestivum*	SAMN08438 749	5 (michiganin)
**131**	*Rathayibacter tritici NCPPB 1953*	1966	Iran	*Triticum aestivum*	SAMN04910 036	5 (michiganin)
**132**	*Sanguibacter inulinus DSM100099.1*	1996	Spain	*Bos taurus*	SAMN16540 220	PAI

No.	Strain	Isolation year	Country of origin	Isolation source	BioSample ID	Locus found
**133**	*Sanguibacter sp. 4.1*	2022	Germany	Irregular necrotic lesions of mature leaves from different deciduous trees	SAMN33443760	PAI
**134**	*Sanguibacter sp. Leaf3*	2013	Switzerland	*Arabidopsis thaliana*	SAMN04151574	PAI
**135**	*Sinomonas humi MUSC 117*	2012	Malaysia	Mangrove	SAMN03070057	5 (michiganin)
**136**	*Streptomyces brasiliscabiei IBSBF2867*	2018	Brazil	*Solanum tuberosum*	SAMN14916383	PAI
**137**	*Streptomyces brasiliscabiei ZRIMU1320*	2021	China	*Solanum tuberosum*	SAMN40272286	PAI
**138**	*Streptomyces brasiliscabiei ZRIMU1321*	2021	China	*Solanum tuberosum*	SAMN40272288	PAI
**139**	*Streptomyces brasiliscabiei ZRIMU1502*	2021	China	*Solanum tuberosum*	SAMN40272232	PAI
**140**	*Streptomyces brasiliscabiei ZRIMU1503*	2021	China	*Solanum tuberosum*	SAMN40272285	PAI
**141**	*Streptomyces brasiliscabiei ZRIMU791*	2021	China	*Solanum tuberosum*	SAMN40272289	PAI
**142**	*Streptomyces caniscabiei ID01-6.2a*	2001	USA	*Solanum tuberosum*	SAMN16083671	PAI
**143**	*Streptomyces caniscabiei ID03-3A*	2003	USA	*Solanum tuberosum*	SAMN16083672	PAI
**144**	*Streptomyces caniscabiei ID05-11E*	2005	USA	*Solanum tuberosum*	SAMN33287981	PAI
**145**	*Streptomyces caniscabiei ID05-16B*	2005	USA	*Solanum tuberosum*	SAMN33287982	PAI
**146**	*Streptomyces caniscabiei ID05-8D*	2005	USA	*Solanum tuberosum*	SAMN33287990	PAI
**147**	*Streptomyces caniscabiei NRRL B-2801*	1961	USA	*Solanum tuberosum*	SAMN06346025	PAI
**148**	*Streptomyces sp. B21-083*	2020	Canada	*Solanum tuberosum*	SAMN34051380	PAI
**149**	*Streptomyces sp. FxanaA7*	unknown	unknown	unknown	SAMN02745829	PAI
**150**	*Streptomyces sp. ID05-04B*	2005	USA	*Solanum tuberosum*	SAMN33287980	PAI
**151**	*Streptomyces sp. ME02-8801-2C*	2002	USA	*Solanum tuberosum*	SAMN33288025	PAI
**152**	*Streptomyces sp. NY05-11A*	2005	USA	*Solanum tuberosum*	SAMN33288078	PAI
**153**	*Streptomyces sp. P3*	2017	South Korea	*Solanum tuberosum*	SAMN08660902	PAI
**154**	*Streptomyces turgidiscabies Car8*	unknown	Japan	*Daucus carota* subsp. *sativus*	SAMN02436476	PAI

Supplementary methods

Supplementary methods S1. Details of the scoring algorithm used for Mexican *Clavibacter michiganensis* strains selection.

The script with the algorithm used for the selection of Mexican representative strains used three steps for the score awarding process. On the first step the script examined the gene families present in the genomes and scored each family according to its average occurrence. The algorithm assigns families a base value ranging from 0 to 1 based on their average prevalence in the genomes: 0 if the gene family was not present in the analyzed genomes and 1 if it was present in all of them. The gene family base value was assigned according to formula 1.

$$a_i = \frac{k_i}{k} \qquad (1)$$

Where a_i is the gene family base value, k_i is the number of genomes where a given gene family is present and k is the total number of genomes analyzed. The purpose of the gene family base value (1) was to grant a higher value to a strain that harbors gene families that are more prevalent in all the strains being analyzed at the same time.

However, since the number of genes a genome has for a given gene family, *i.e.* the gene copy number, is also variable a second score that measures in each family the deviation from the most popular (the mode) copy number value was used. Thus, in the second step a modifier value for each observed copy number in the gene families was calculated using the formula 2.

$$b_{ji} = 1 - \frac{\left|c_j - c_i\right|}{\frac{c_j + c_i}{2}} \qquad (2)$$

Where b_{ji} is the gene family modifier value for a given gene copy number j in a gene family i, c_j is the frequency of a given gene copy number j and c_i is the copy number mode value for the gene family i. The gene family modifier value (2) ensured that a strain received a full gene family base value for a given gene family only if it had the most popular gene copy number in said gene family at the script's final step.

The final score for a strain's genome was given by total score formula (3), which combined the gene family base value (1) and modifier value (2) for each gene family in a strain.

$$S_m = \sum_{i=1}^{n} a_i b_{mi} \qquad (3)$$

Where S_m is the total score of a genome *m*; n is the number of gene families in the analyzed dataset; a_i is the base value of a gene family *i* and b_{mi} is the modifier value of the gene family based on the copy number the genome *m* has for the gene family *i*.

The script was used to analyze each clade of the Mexican *Clavibacter michiganensis* strains separately. The strain or strains with the highest score in each analysis were selected. The script can be found at https://github.com/a-yanez/Clavibacter_project.

Supplementary material references

1. Thapa SP, O'Leary M, Jacques M-AA, Gilbertson RL, Coaker G, O'Leary M, et al. Comparative genomics to develop a specific multiplex PCR assay for detection of clavibacter michiganensis. Phytopathology [Internet]. 2020 Mar;110(3):556–66. Available from: https://doi.org/10.1094/PHYTO-10-19-0405-R

2. Valenzuela M, González M, Velásquez A, Dorta F, Montenegro I, Besoain X, et al. Analyses of virulence genes of clavibacter michiganensis subsp. Michiganensis strains reveal heterogeneity and deletions that correlate with pathogenicity. Microorganisms [Internet]. 2021 Jul 18 [cited 2021 Dec 13];9(7):1530. Available from: https://www.mdpi.com/2076-2607/9/7/1530/htm

3. Tian Q, Chuan J, Sun X, Zhou A, Wang L, Zou J, et al. Description of Clavibacter zhangzhiyongii sp. nov., a phytopathogenic actinobacterium isolated from barley seeds, causing leaf brown spot and decline. Int J Syst Evol Microbiol [Internet]. 2021 May 13;71(5). Available from: https://www.microbiologyresearch.org/content/journal/ijsem/10.1099/ijsem.0.004786

4. Załuga J, Stragier P, Baeyen S, Haegeman A, Van Vaerenbergh J, Maes M, et al. Comparative genome analysis of pathogenic and non-pathogenic Clavibacter strains reveals adaptations to their lifestyle. BMC Genomics. 2014;15(1):392.

5. Thapa SP, Pattathil S, Hahn MG, Jacques M-A, Gilbertson RL, Coaker G. Genomic Analysis of Clavibacter michiganensis Reveals Insight Into Virulence Strategies and Genetic Diversity of a Gram-Positive Bacterial Pathogen. Mol Plant-Microbe Interact [Internet]. 2017 Oct;30(10):786–802. Available from: https://apsjournals.apsnet.org/doi/10.1094/MPMI-06-17-0146-R

6. Davis EW, Tabima JF, Weisberg AJ, Lopes LD, Wiseman MSMS, Wiseman MSMS, et al. Evolution of the U.S. biological select agent Rathayibacter toxicus. Vidaver AK, editor. MBio [Internet]. 2018 Aug 28;9(4):1–16. Available from: http://mbio.asm.org/lookup/doi/10.1128/mBio.01280-18

7. Lu Y, Samac DA, Glazebrook J, Ishimaru CA. Complete genome sequence of Clavibacter michiganensis subsp. insidiosus R1-1 using PacBio single-molecule realtime technology. Genome Announc [Internet]. 2015 Jun 25;3(3):80. Available from: http://genomea.asm.org/lookup/doi/10.1128/genomeA.00396-15

8. Bragard C, Dehnen-Schmutz K, Di Serio F, Gonthier P, Jaques Miret JA, Justesen AF, et al. Pest categorisation of Clavibacter sepedonicus. EFSA J. 2019;17(4).

9. Oh EJ, Bae C, Lee HIHB, Hwang IS, Lee HIHB, Yea MC, et al. Clavibacter michiganensis subsp. Capsici subsp. nov., causing bacterial canker disease in pepper. Int J Syst Evol Microbiol. 2016;66(10):4065–70.

10. Li XS, Yuan XK. Genome Sequences for Multiple Clavibacter Strains from Different Subspecies. Genome Announc [Internet]. 2017 Sep 21;5(38):2–3. Available from: http://genomea.asm.org/lookup/doi/10.1128/genomeA.00721-17

11. Gartemann K-H, Abt B, Bekel T, Burger A, Engemann J, Flügel M, et al. The Genome Sequence of the Tomato-Pathogenic Actinomycete Clavibacter michiganensis subsp. michiganensis NCPPB382 Reveals a Large Island Involved in Pathogenicity. J Bacteriol [Internet]. 2008 Mar 15;190(6):2138–49. Available from:

https://journals.asm.org/doi/10.1128/JB.01595-07

12. Tarlachkov S V., Starodumova IP, Dorofeeva L V., Prisyazhnaya N V., Roubtsova T V., Chizhov VN, et al. Draft Genome Sequences of 28 Actinobacteria of the Family Microbacteriaceae Associated with Nematode-Infected Plants . Microbiol Resour Announc. 2021;10(9):28–30.

Supplementary figures

Supplementary figure S1. Tomato sampling sites in Mexico.

Supplementary figure S2. Isolates obtained from wild tomato variety populations.

Supplementary figure S3. Average Nucleotide Identity analysis of *Clavibacter* genus.

Supplementary figure S4. Phylogeny of *Clavibacter michiganensis*.

Supplementary figure S5. Phylogeny of the genus *Clavibacter* and rooted reference-phylogeny.

Supplementary figure S6. Blastp hits for the *C. michiganensis* conserved gene families in the NCBI non-redundant database.

Supplementary figure S7. *C. michiganensis* locus 2 homologs found outside the *Clavibacter* genus.

Supplementary figure S8. *C. michiganensis* loci homologs found outside the *Clavibacter* genus.

Supplementary figure S9. *C. michiganensis* PAI tomA subregion partial homologs loci found outside the *Clavibacter* genus.

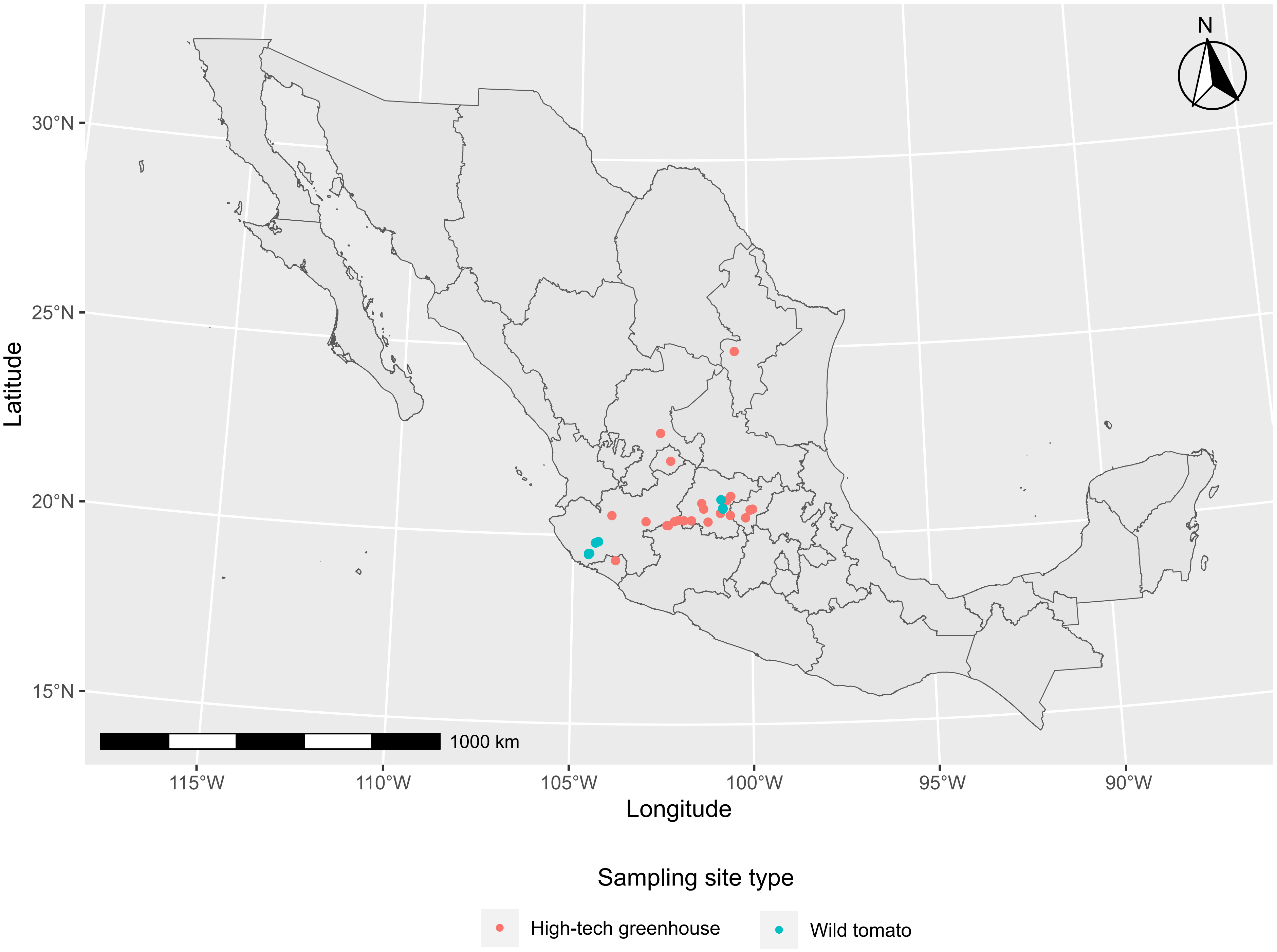

Supplementary figure S1. Tomato sampling sites in Mexico. Samples metadata can be found in **Supp. tables S1** and **S2.**

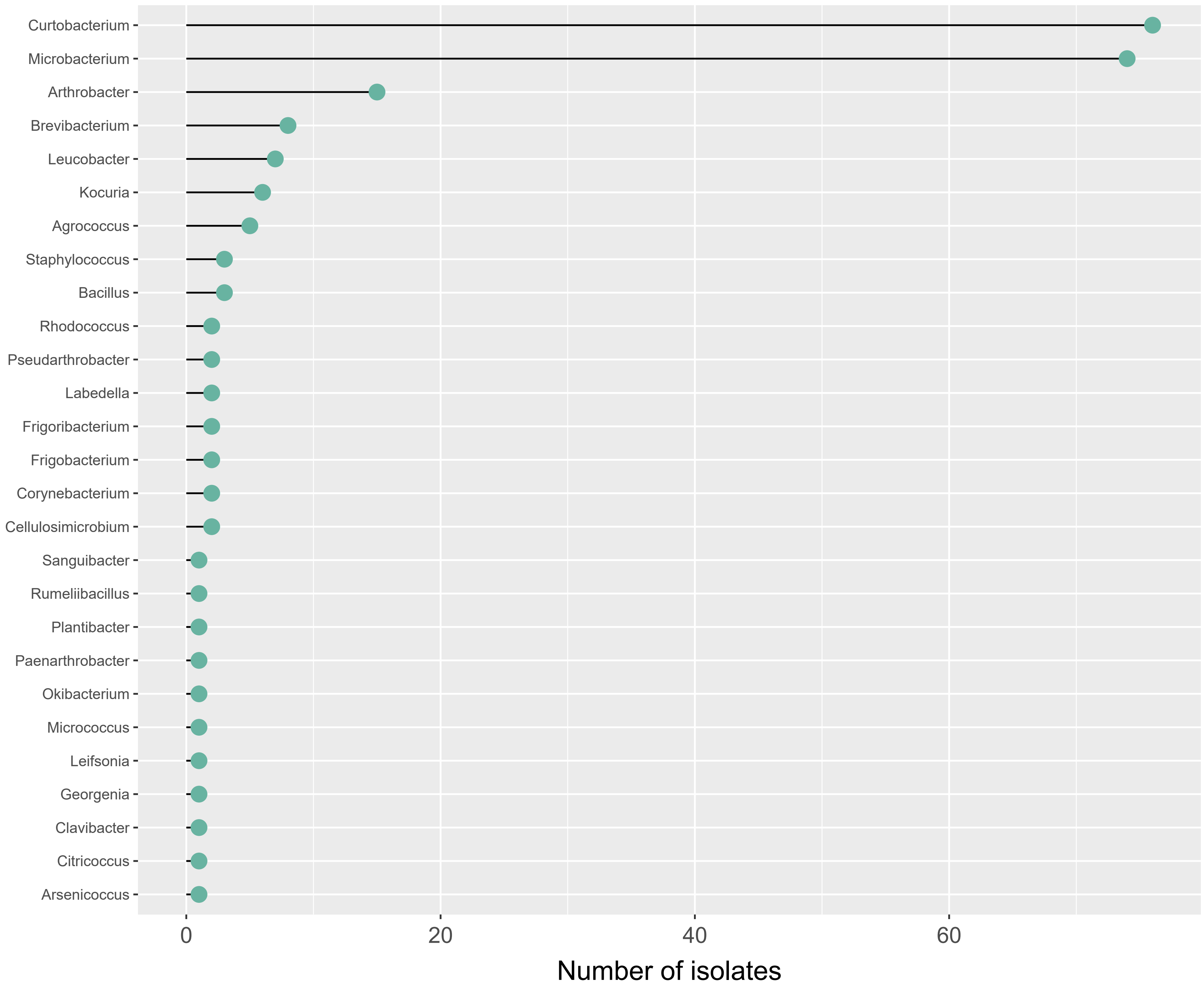

Supplementary figure S2. Isolates obtained from wild tomato variety populations. Only 1 Clavibacter strain (RA1B) could be retrieved from the sampling.

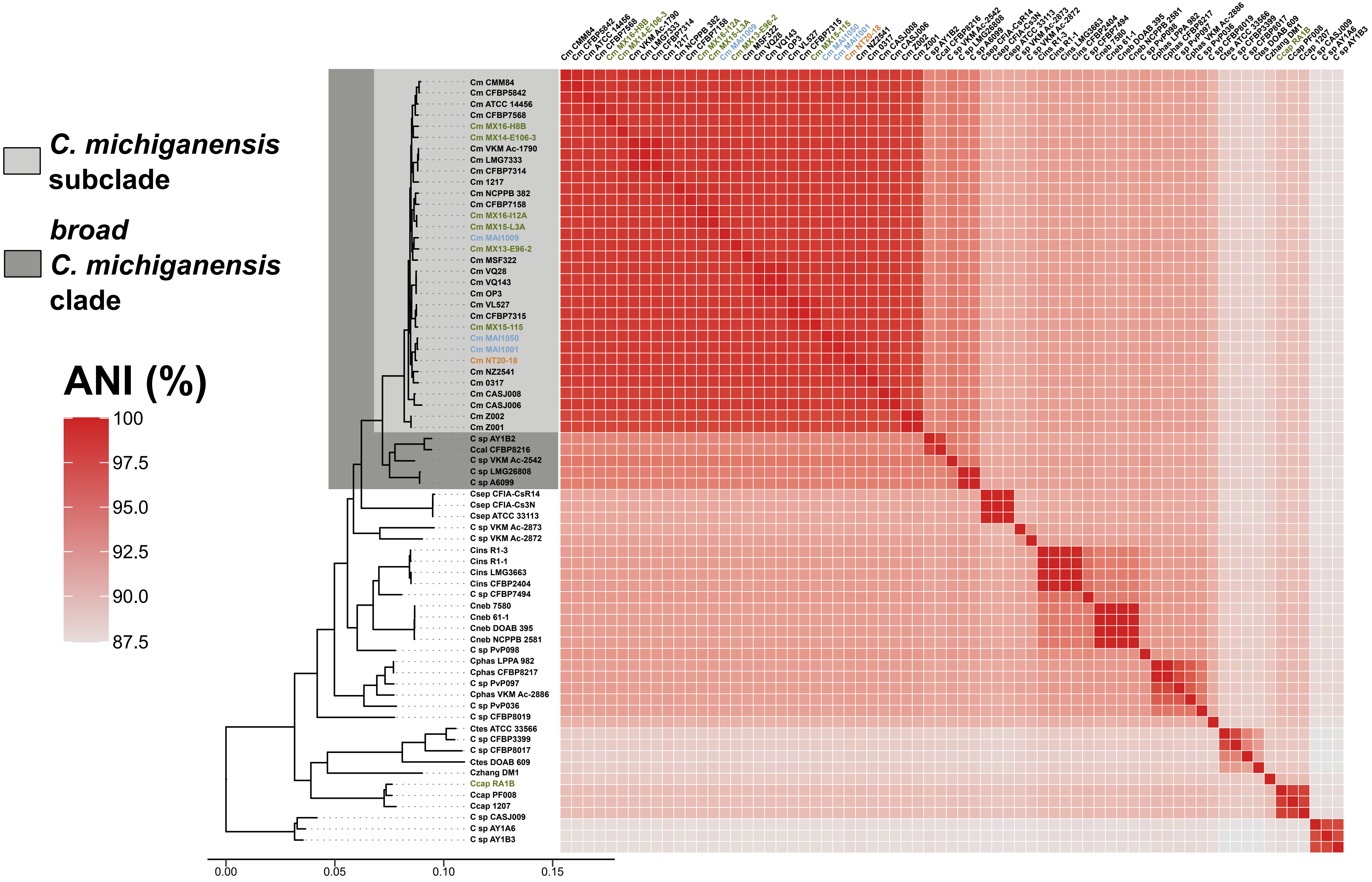

Supplementary figure S3. Average Nucleotide Identity analysis of Clavibacter genus. Heatmap showing an ANI pairwise comparison using PyANI. Rows and columns are ordered according to the genus phylogeny. Names colored other than black indicate the genomes obtained by us and published as part of this study. The colors in the names correspond to the strains' place of origin: orange - the Netherlands, blue – Uruguay, and green - Mexico.

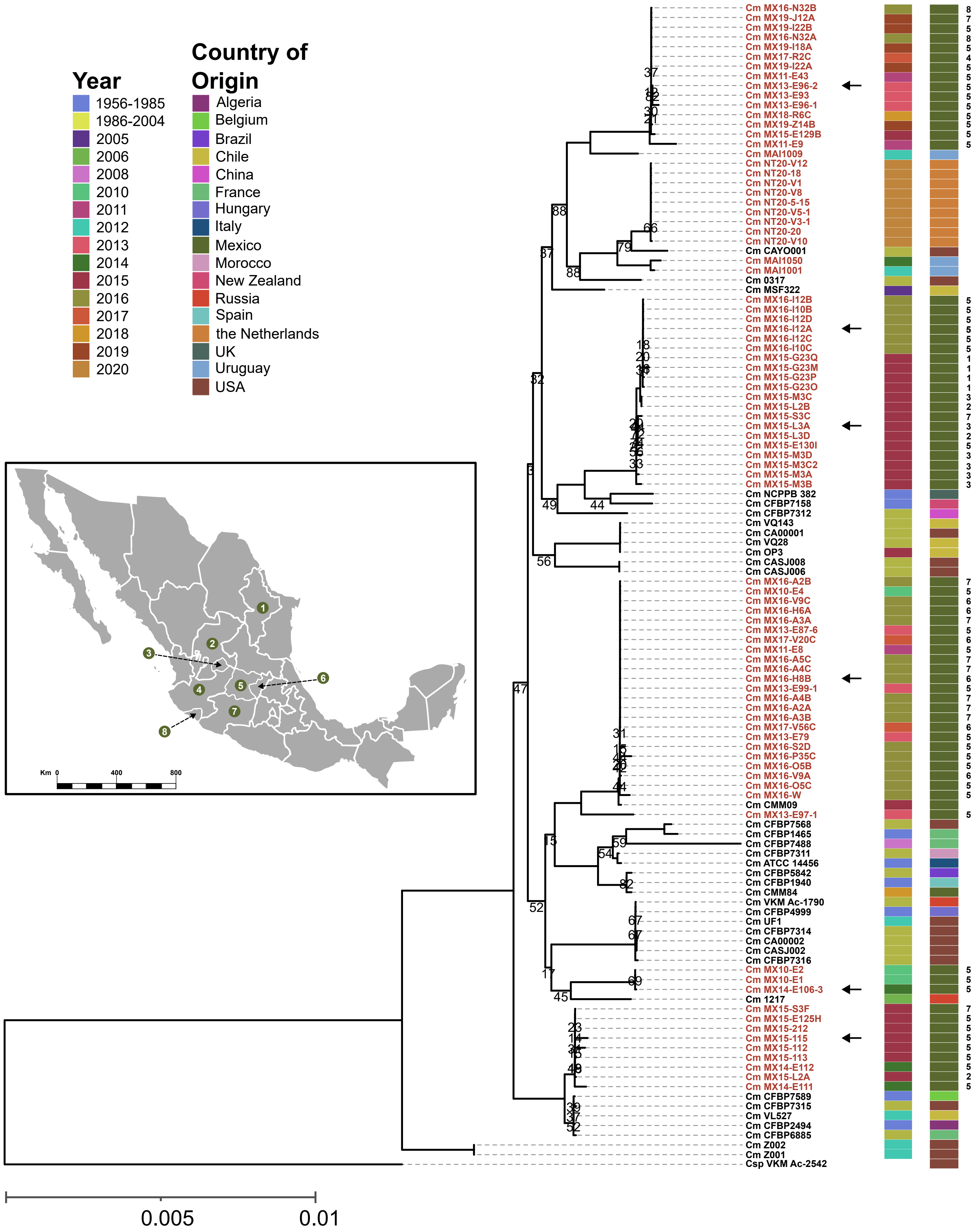

Supplementary figure S4. Phylogeny of *Clavibacter michiganensis*. *Clavibacter* strain VKM Ac-2542 was used as root. Only boostrap values <90% are displayed. Mexican strains selected for the genus-level analysis as indicated with arrows. Names of the genomes released as part of this manuscript are highlighted in red. Color-coded rectangles correspond to the strains' metadata regarding their year of isolation and country of origin. Numbers to the right of the metadata rectangles indicate the specific origin for the Mexican strains accorrding to the map on the left: 1 = Nuevo Leon, 2 = Zacatecas, 3 = Aguascalientes, 4 = Jalisco, 5 = Guanajuato, 6 = Queretaro, 7 = Michoacan, 8 = Colima.

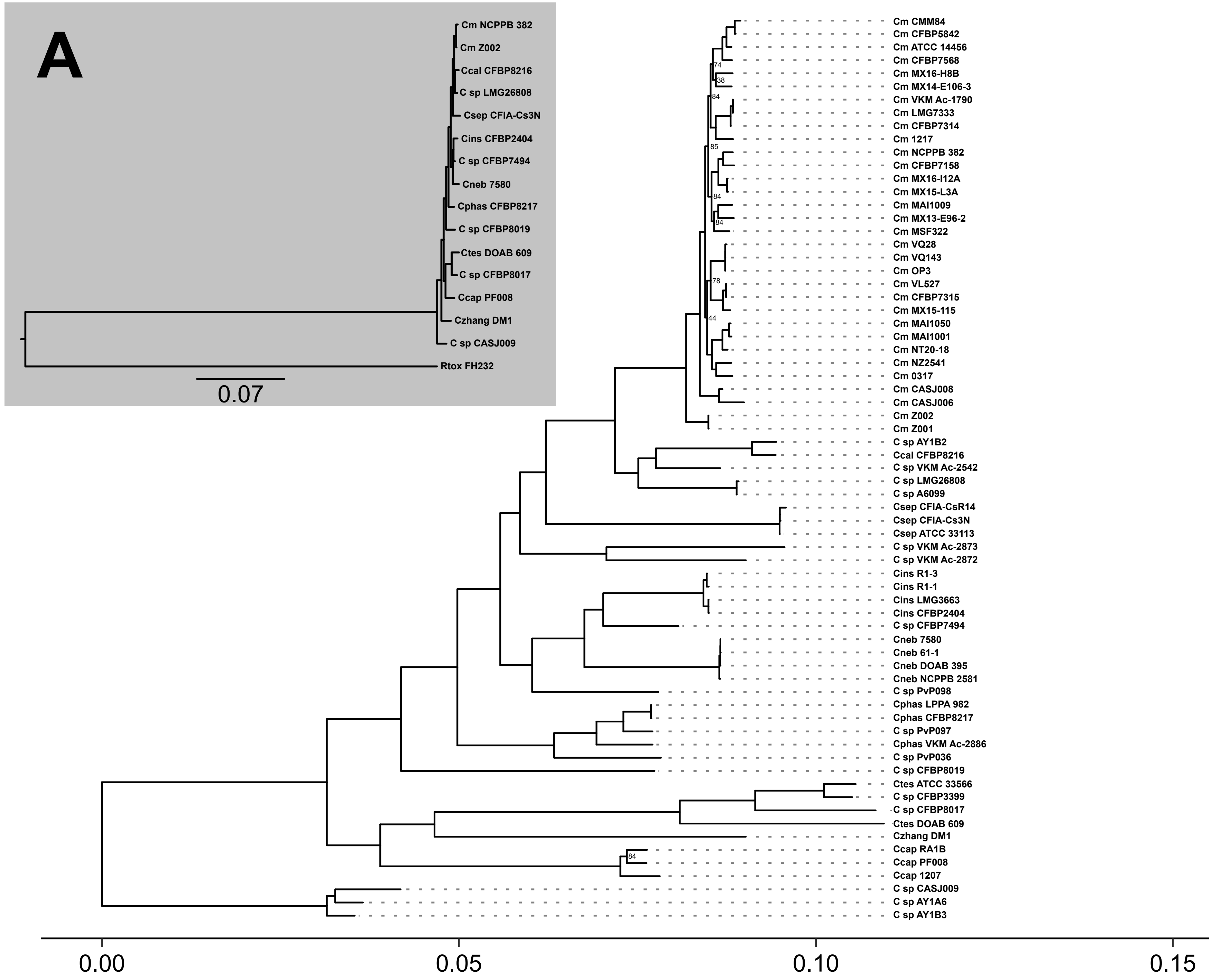

Supplementary figure S5. Phylogeny of the genus *Clavibacter*. Family-level reference phylogeny with *Rathayibacter toxicus* FH232 (Rtox FH232) in Subpanel A was used to order the *Clavibacter* genus phylogeny comprised of 69 genomes. Only bootstrap values <90% are displayed. Abbreviations: Cm - *C. michiganensis,* C sp. - *Clavibacter* sp., Ccal - *C. californiensis,* Csep - *C. sepedonicus,* Cneb - *C. nebraskensis,* Cins - *C. insidiosus,* Cphas - *C. phaseoli,* Czhang - *C. zhanzhiyongii,* Ccap - *C. capsici,* Ctes - *C. tessellarius*.

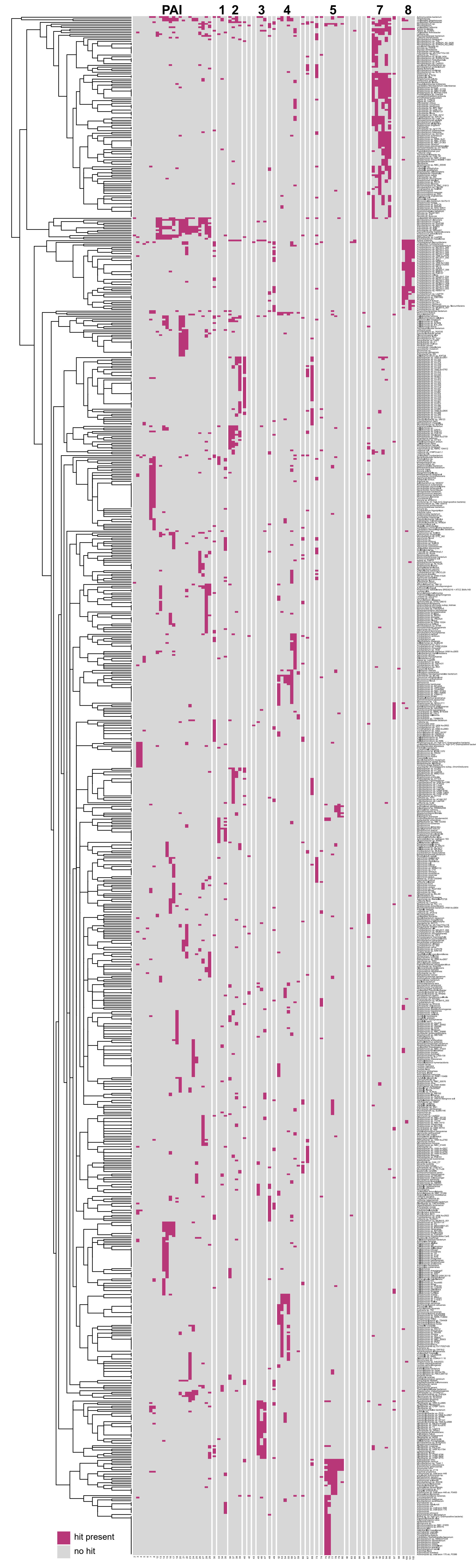

Supplementary figure S6. Hits for the *C. michiganensis* conserved gene families in the NCBI non-redundant database. Each row represents a different OTU and each column a different gene family. Columns are clustered if the gene families belong to the same loci. Hits were found for only 76 out of 103 gene families. Hierarchical clustering was used to group the taxa by their hit similarity. Less than 15% hits for the 103 gene families were found in almost every OTU except for *Actinomycetota* bacterium (24.27%), *Curtobacterium flaccumfaciens* (16.5%), *Microbacterium* sp. (18.44%), *Streptomyces* (21.35%), Unclassified *Microbacterium* (17.47%) and Unclassified *Streptomyces* (20.38%). No hits were found for loci 6.

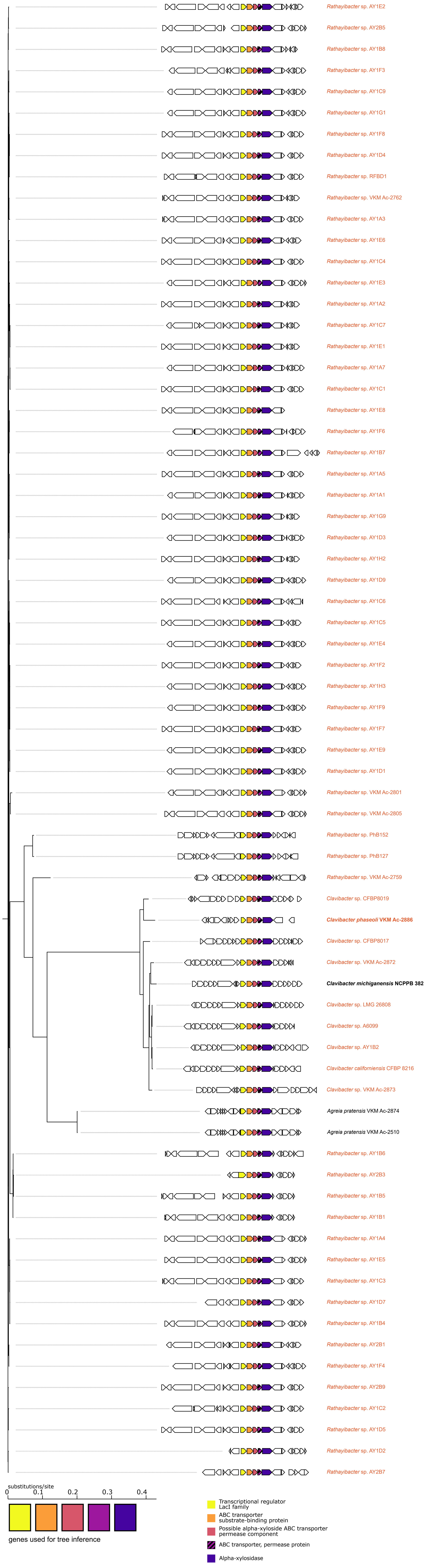

Supplementary figure S7. *C. michiganensis* locus 2 homologs found outside the *Clavibacter* genus. The original colors of the CORASON output were modified to show the conserved genes in each loci. The color of the genes employed by CORASON to infer the phylogenetic tree to the left is shown in the bottom left corner. The stripped pattern over some genes indicates that these and their homologs are not part of the *C. michiganensis* conserved genes found in the pangenomic analysis. Names of plant pathogenic strains are shown in bold while strains from genera with known plant pathogenic members are shown in orange, except for *C. michiganensis* NCPPB 382 (bold and black)

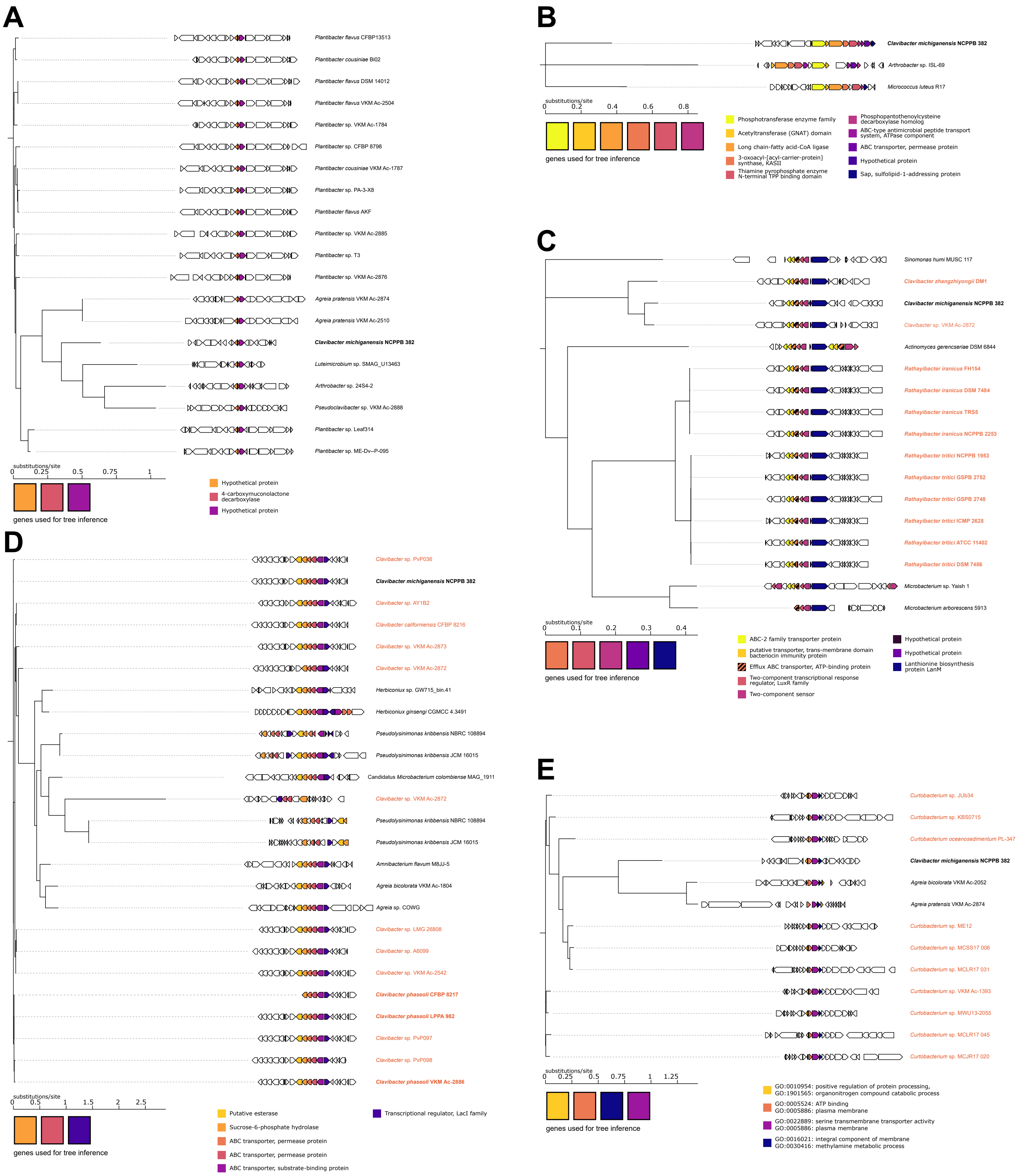

Supplementary figure S8. *C. michiganensis* loci homologs found outside the *Clavibacter* genus. The original colors of the CORASON output were modified to show the conserved genes in each loci. The color of the genes employed by CORASON to infer the phylogenetic tree to the left is shown in the bottom left corner. The stripped pattern over some genes indicates that these and their homologs are not part of the *C. michiganensis* conserved genes found in the pangenomic analysis. Names of plant pathogenic strains are shown in bold while strains from genera with known plant pathogenic members are shown in orange, except for *C. michiganensis* NCPPB 382 (bold and black). **A.** Locus 3 homologs. **B.** Locus 4 (michivionin) homologs. **C.** Locus 5 (michiganin) homologs. **D.** Locus 7 homologs. **E.** Locus 8 homologs.

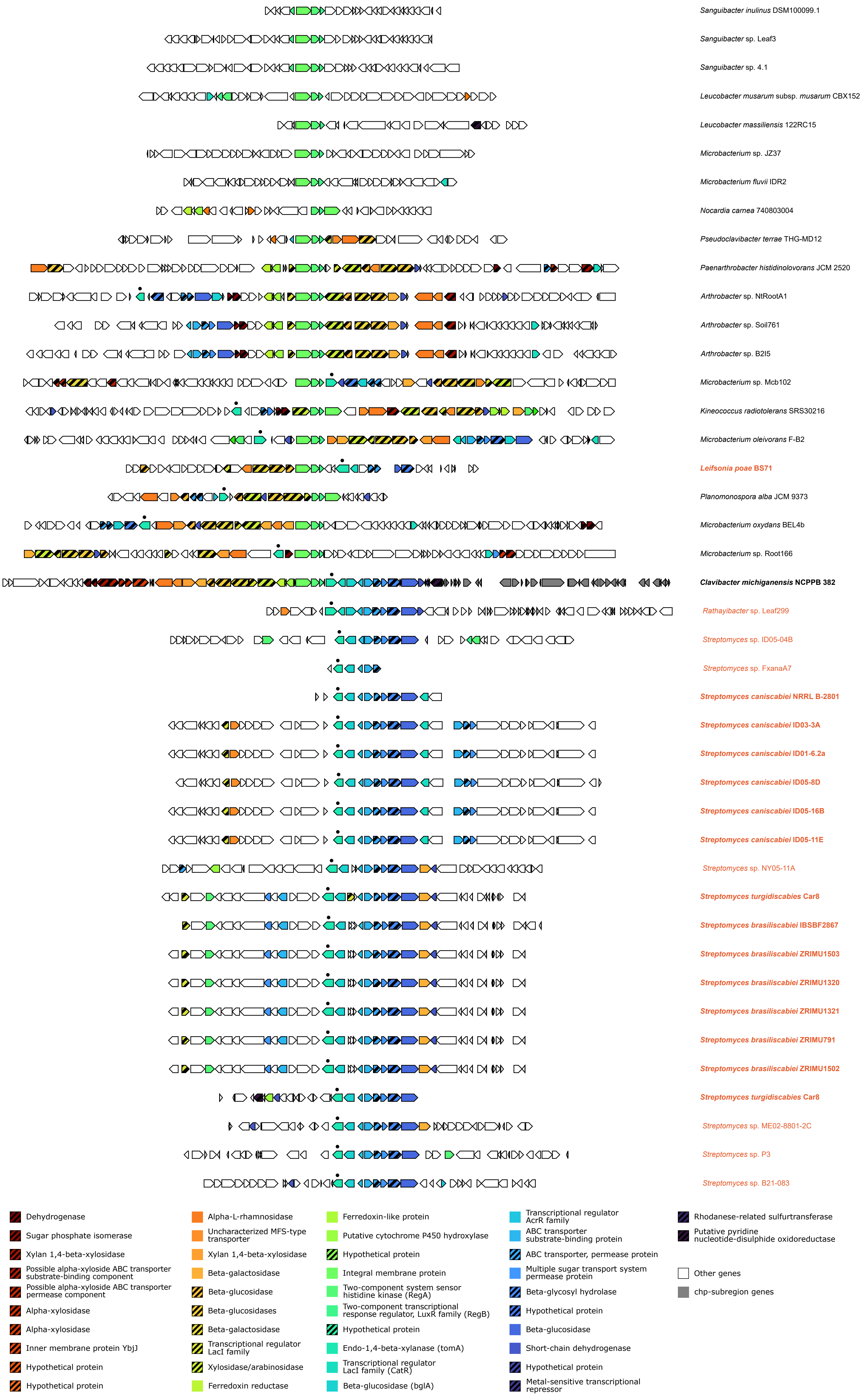

Supplementary figure S9. *C. michiganensis* PAI tomA subregion partial homologs loci found outside the *Clavibacter* genus. The original colors of the CORASON output were modified to show the conserved genes in each loci. tomA gene homologs are indicated with a black circle. The stripped pattern over some genes indicates that these and their homologs are not part of the C. michiganensis conserved genes found in the pangenomic analysis. Names of plant pathogenic strains are shown in bold while strains from genera with known plant pathogenic members are shown in orange, except for *C. michiganensis* NCPPB 382 (bold and black).
